# Fast and Accurate Taxonomic Assignments of Metagenomic Sequences Using MetaBin

**DOI:** 10.1371/journal.pone.0034030

**Published:** 2012-04-04

**Authors:** Vineet K. Sharma, Naveen Kumar, Tulika Prakash, Todd D. Taylor

**Affiliations:** 1 Laboratory for MetaSystems Research, Quantitative Biology Center, RIKEN, Yokohama, Kanagawa, Japan; 2 Department of Biological Sciences, Indian Institute of Science Education and Research (IISER), Bhopal, Madhya Pradesh, India; Wayne State University, United States of America

## Abstract

Taxonomic assignment of sequence reads is a challenging task in metagenomic data analysis, for which the present methods mainly use either composition- or homology-based approaches. Though the homology-based methods are more sensitive and accurate, they suffer primarily due to the time needed to generate the Blast alignments. We developed the MetaBin program and web server for better homology-based taxonomic assignments using an ORF-based approach. By implementing Blat as the faster alignment method in place of Blastx, the analysis time has been reduced by severalfold. It is benchmarked using both simulated and real metagenomic datasets, and can be used for both single and paired-end sequence reads of varying lengths (≥45 bp). To our knowledge, MetaBin is the only available program that can be used for the taxonomic binning of short reads (<100 bp) with high accuracy and high sensitivity using a homology-based approach. The MetaBin web server can be used to carry out the taxonomic analysis, by either submitting reads or Blastx output. It provides several options including construction of taxonomic trees, creation of a composition chart, functional analysis using COGs, and comparative analysis of multiple metagenomic datasets. MetaBin web server and a standalone version for high-throughput analysis are available freely at http://metabin.riken.jp/.

## Introduction

Metagenomics has emerged as a powerful culture-independent approach for exploring the complexity and diversity of microbial genomes in their natural environments [1]. Globally, several hundred metagenomic projects are either ongoing or are in the planning stages. These projects generate huge amounts of sequence reads of various lengths depending upon the methodology used. Though the primary aim of these studies is usually to capture a snapshot of the entire microbial community that exists in an environment, current methodologies commonly only generate a complex mixture of short genomic sequences derived from several different genomes found within that environment. The situation becomes more complicated when many of the sequences come from novel or yet uncultured species, for which the genomes are not well represented in the reference databases. Therefore, one of the first and most crucial tasks is to ascertain the genomic origin of these sequences, and to make appropriate taxonomic assignments.

There are two main approaches currently used for the taxonomic assignments of metagenomic reads. The first approach, employed by classification algorithms such as PhyloPythia, TETRA, NBC and TACOA, exploits sequence composition for taxonomic classification of metagenomic sequences [2]–[5]. The second approach assesses the taxonomic identity of a read from the results of a homology-based search against the known reference sequence database (usually NCBI non redundant (NR) database) [6]. Of these, the most commonly used tool, MEGAN, carries out taxonomic binning based on the NCBI BLAST [6] bit-score using Lowest Common Ancestor (LCA) based approach [7]. It assigns a read to the common taxonomic ancestor (higher taxonomic level) of the hits if the read shows hits with multiple genomes. Because the consideration of hits is only based on bit-scores, this may lead to higher number of non-specific taxonomic assignment due to consideration of both expected (correct) and unexpected (higher taxonomic level) hits. Another similar algorithm, SOrt-ITEMS, applies a sequence orthology-based approach in addition to the LCA method [8]. It uses only the aligned regions, which in the case of short reads containing only partial ORFs are incomplete and are insufficient to accurately deduce sequence orthology, available in the Blastx output. WebCARMA is another method that looks for conserved Pfam domains and protein families in the metagenomic reads using a homology-based search [9].

Though faster in execution, the composition-based methods generally suffer from several limitations. For example, prior training sequence sets are needed, the classifications are only applicable for longer sequences (>800 bp in length) in most cases, and the classifications are limited to higher taxonomic levels. Even for recent methods like Phymm, which uses a hybrid approach using interpolated Markov models (IMMs) followed by BLAST search, taxonomic classification is limited to read lengths of ≥100 bp, with few correct assignments at the genus level [10]. Likewise, the major limitation for using most homology-based methods is that the total analysis time is exceedingly long since they require the alignment results as input, and it takes a long time to align query sequences by BLAST against the ever-increasing NCBI NR reference database. To some extent, performance is also limited by dependence on the representation of genomes and their respective sequences in the reference databases. However, even when a genus is absent, but other genomes from the same family of the same genus or a higher taxonomic level are present, classification to the correct taxonomic lineage can still be made. Dependence on available reference genomes is also a drawback for composition-based methods, since they are needed for prior training. Overall, homology-based methods are able to carry out classification at deeper taxonomic levels (family, genus or species) and are not limited by sequence read length, as opposed to composition-based methods.

In this study we present the ‘MetaBin’ program and web server, which uses a significantly improved homology-based algorithm for taxonomic analysis. It employs a unique ORF (Open Reading Frame)-based approach for carrying out taxonomic assignments and it implements Blat [11] (or if the user prefers, Blastx) for generating the alignments, together resulting in several folds faster, accurate, specific and highly sensitive taxonomic classification. It can be used for various read lengths (≥45 bp) obtained from commonly used sequencing technologies such as Sanger, Roche 454, Illumina Solexa, or others, including paired-end reads.

## Methods

### Test sequences and database construction

The Non-Redundant (NR) sequence database (ftp://ftp.ncbi.nih.gov/blast/db/FASTA/, August, 2010) and sequences of 25 completed bacterial genomes and two archaea genomes belonging to different taxonomic lineages (Table S1) (ftp.ncbi.nih.gov/genomes/Bacteria) were retrieved from NCBI [6]. To test the performance of MetaBin, local versions of the NR database were created by removing all sequences belonging to the associated genus and the associated family (Text S2). These are referred to as NRminusGenus and NRminusFamily, respectively, in the subsequent text. The NRminusGenus and NRminusFamily databases mimic the situation where the genus or family of the considered microbial genome is unknown. Therefore, the reads derived from these genomes can be considered as reads of novel or yet unknown genomes because the NRminusGenus and NRminusFamily databases do not contain any genome of that genus or family, respectively. This provides us with a test scenario for assigning taxonomy to such reads for which no genome of that genus or family is present in the NR database, and helps us to examine the performance of MetaBin on novel genomes.

### Construction of simulated read datasets

To test the performance of MetaBin, we constructed simulated reads datasets from 27 microbial (25 bacterial and two archaeal) genomes belonging to diverse taxonomic groups (NCBI Taxonomy Browser, http://www.ncbi.nlm.nih.gov/Taxonomy/). Of these genomes, RSD17 and CFP2 are unculturable endosymbiotic bacteria found in termite gut [12], [13]. RSD17 belongs to the phylum Elusimicrobia and, since it is the first and only sequenced genome from this community, its genus is yet unidentified; and other closely related genomes from this phylum are underrepresented in the NR database. CFP2 belongs to a known genus (Candidatus Azobacteroides), but it is the only known genome from this genus; however, its phylum (Bacteroidetes) contains several known genomes. Similarly, CAPH, DITH, and GEAU are the only sequenced genomes available from their respective genus. These genomes provide additional test scenarios for us to examine the performance of MetaBin on novel genomes. We created a set of 702,000 simulated synthetic reads of various lengths ranging from 45–800 bp from the 27 microbial genomes. The MetaSim program was used to generate reads to represent Sanger (read length ∼800 bp) and 454 (read lengths of ∼400 and ∼250 bp) sequences [14]. For each of the 27 microbial genomes, 1,000 reads of 800 bp, 2,000 reads of 400 bp, 3,000 reads of 250 bp, and 10,000 reads each of length 75 and 45 bp were generated. Since there is no available option to generate Illumina-like reads in MetaSim, we developed our own Perl script for generating simulated reads of length ∼75 bp and ∼45 bp.

### Retrieval of published metagenomic data

The human gut metagenomic data obtained by Illumina sequencing from a single Spanish male individual (V1.CD-2, age 49, BMI 27.76, 20,707,369 high quality reads, library 090107) was retrieved (ftp://public.genomics.org.cn/BGI/gutmeta/High_quality_reads/) and is referred to as ‘V1CD2’ in the subsequent text [15]. The metagenomic sequences (Sanger reads) for human gut samples F1-S (Adult male, age 30 yo) and F1-T (Adult female, age 28 yo), members of the same family, were downloaded from the DDBJ database (ftp://ftp.ddbj.nig.ac.jp/ddbj_database/dta/UTCOB/) [16]. A total of 5,000 paired-end reads from each of these samples were used for comparative analysis of MetaBin with the other programs. These datasets are referred to as HGF1S and HGF1T in the subsequent text. The sample data sequences (Sargasso Sea Subsample 1, Sanger reads) for Sargasso Sea, as described and analyzed in the MEGAN manuscript, were downloaded from http://www-ab.informatik.uni-tuebingen.de/software/megan/old-datasets [7]. This set contains the first 10,000 reads from Sample 1 of the Sargasso Sea dataset [17] and is referred to as ‘SSea sample 1’ in the subsequent text. The first dataset, V1CD2, was aligned using Blat against NCBI NR. The other three datasets HGF1S, HGF1T, and ‘SSea sample 1’ were aligned with NCBI NR by Blastx.

### BLAST and BLAT analysis

BLAST (version 2.2.22, ftp://ftp.ncbi.nih.gov/blast/) was obtained from NCBI. The parameters used to run Blastx were: word size adjustment ‘–W 2 –f 8’, soft filtering setting ‘-F “m S”’ and expectation value ‘-E 100’ to allow inclusion of short matches. We recommend these parameters while running Blastx for comprehensive taxonomic assignments using the MetaBin algorithm [7]. BLAT (version 34, http://genome-test.cse.ucsc.edu/~kent/exe/) was also obtained and used for the analysis. These two alignment programs were both integrated with the web-based version of MetaBin.

### Functional analysis using COGs

The in-house reference dataset for COGs (Cluster of Orthologous Groups of proteins) (referred to as ‘COGs-DB’) was constructed by using information from 1,230 microbial genomes available at NCBI (ftp.ncbi.nih.gov/genomes/Bacteria, Dec 2010). The COGs information is inferred from COGs-DB for the best hit for a read. If no COGs could be assigned, the gene product is considered to be ‘uncharacterized’. The frequency of each COG is then counted up for every dataset. For the reads containing partial ORFs, the hit counts of COGs are corrected by the length ratio of each ‘partial ORF’ to the reference protein to minimize multiple counts of fragmented genes. The size of each COG is normalized by the total number of COGs predicted in each dataset (‘Cnormalized%’). The average size of each COG in COGs-DB is also calculated and normalized by the total number of COGs in COGs-DB (‘CDB %’). Finally, the magnitude of enrichment (enrichment value) of each COG is calculated for every microbiome by dividing the ‘Cnormalized%’ by the ‘CDB%’. COGs with an average enrichment value of ≥2.0 were defined as enriched COGs in each microbiome [16].

### Other publicly available taxonomic binning programs

MEGAN (version 3.8) (http://www-ab.informatik.uni-tuebingen.de/data/software/megan/download/welcome.html), Sort-ITEMS (http://metagenomics.atc.tcs.com/binning/SOrt-ITEMS) [8], and TACOA (version 1.0, http://www.cebitec.uni-bielefeld.de/brf/tacoa/tacoa.html) [4] were downloaded from their respective sites. WebCARMA and NBC were run from their web servers (http://webcarma.cebitec.uni-bielefeld.de/cgi-bin/webcarma.cgi) and (http://nbc.ece.drexel.edu), respectively [5], [9]. The performance of MetaBin was compared with MEGAN and SOrt-ITEMS for various simulated read datasets using similar parameters (bin size of at least one read and minimum bit-score of 29). However, for the comparative analysis of the ‘SSea sample 1’ data, all three programs were used with a minimum bit-score of 35, as used previously [7].

### Algorithm development

**Figure 1:**
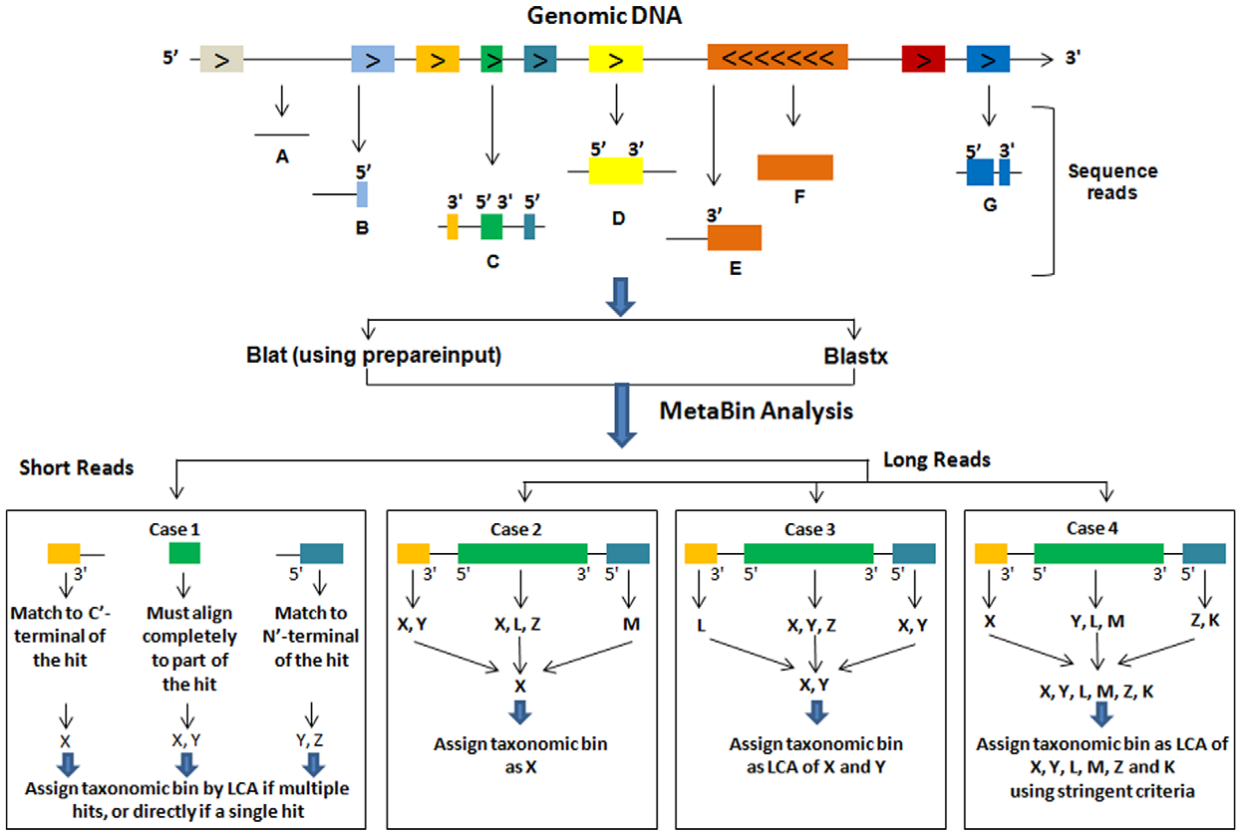
ORF-based approach for the taxonomic assignment of reads of different lengths derived from different regions of the genomic DNA. Read derived from intergenic region (A), read containing the small 5′ region of an ORF (B), read containing two partial ORFs at the 5′and 3′ terminals and a complete ORF in the middle (C), read containing only a single complete ORF (D), read containing a long partial ORF at one end (E), read obtained from within an ORF (F), read with sequencing error causing a single ORF to split into two smaller ORFs (G). X, Y, Z, K, L, and M are the genomes to which the ORFs showed matches. The taxonomic IDs of the species of these genomes are used for making the taxonomic assignments, and for creating the taxonomic bins.

Our main motivations for developing MetaBin were to provide significant improvements over currently existing homology-based methods for better taxonomic assignments, and to dramatically reduce the amount of time needed to generate the alignments usually made by Blastx. The later objective was achieved by implementing Blat as the faster alignment method in place of Blastx (both options are available), reducing the analysis time by up to 1000-fold. This feature makes it finally practical to use a more accurate and sensitive homology-based approach for both web- and console-based high-throughput analysis of large datasets. To achieve the first objective, we used the following strategy. Commonly, using a homology-based approach, a sequence is first aligned against a reference database (usually NCBI NR), and then its taxonomic identity is inferred based on the taxonomic information of the most significant match (or hit protein) found in the reference database. Importantly, since the alignments, by Blat or Blastx, are local and a read may contain multiple coding regions (complete or partial) (Figure 1), it is possible that different parts of the query align with different proteins in the NR database. Furthermore, the shotgun sequencing approach is likely to generate DNA fragments from various regions, including the intragenic and intergenic regions. Therefore, all possibilities (A–G) represented in Figure 1 are likely to occur and must be considered for taxonomic assignments. These are discussed in detail in Text S1. To consider these possibilities and assign them to the correct taxonomic bins, we have used a unique approach that considers the taxonomic information from all complete or partial ORFs present in a read, and then assign it to a taxonomic bin.

Case 1 is applicable for shorter reads (45–75 bp) which do not contain multiple or complete ORFs, but contain only a single partial ORF which may originate from one of the terminals or any other region of the protein. In this case, MetaBin employs very stringent criteria and considers only those partial ORFs which either match to the N- or C- terminals of the hit protein, or almost completely match with high identity to other regions of the hit protein. All other partial ORFs are discarded, leading to more accurate assignments for shorter reads. For case 2, if two or more ORFs come from the same genome, the taxonomic ID (TID) of the common genome (X) is assigned as the taxonomic bin. For case 3, when two or more ORFs show a common match to two or more genomes, then the LCA of the two genomes (X and Y) is assigned. For case 4, when the different ORFs have no commonly matching genomes, then the LCA of all the genomes is assigned as the taxonomic bin.

**Figure 2:**
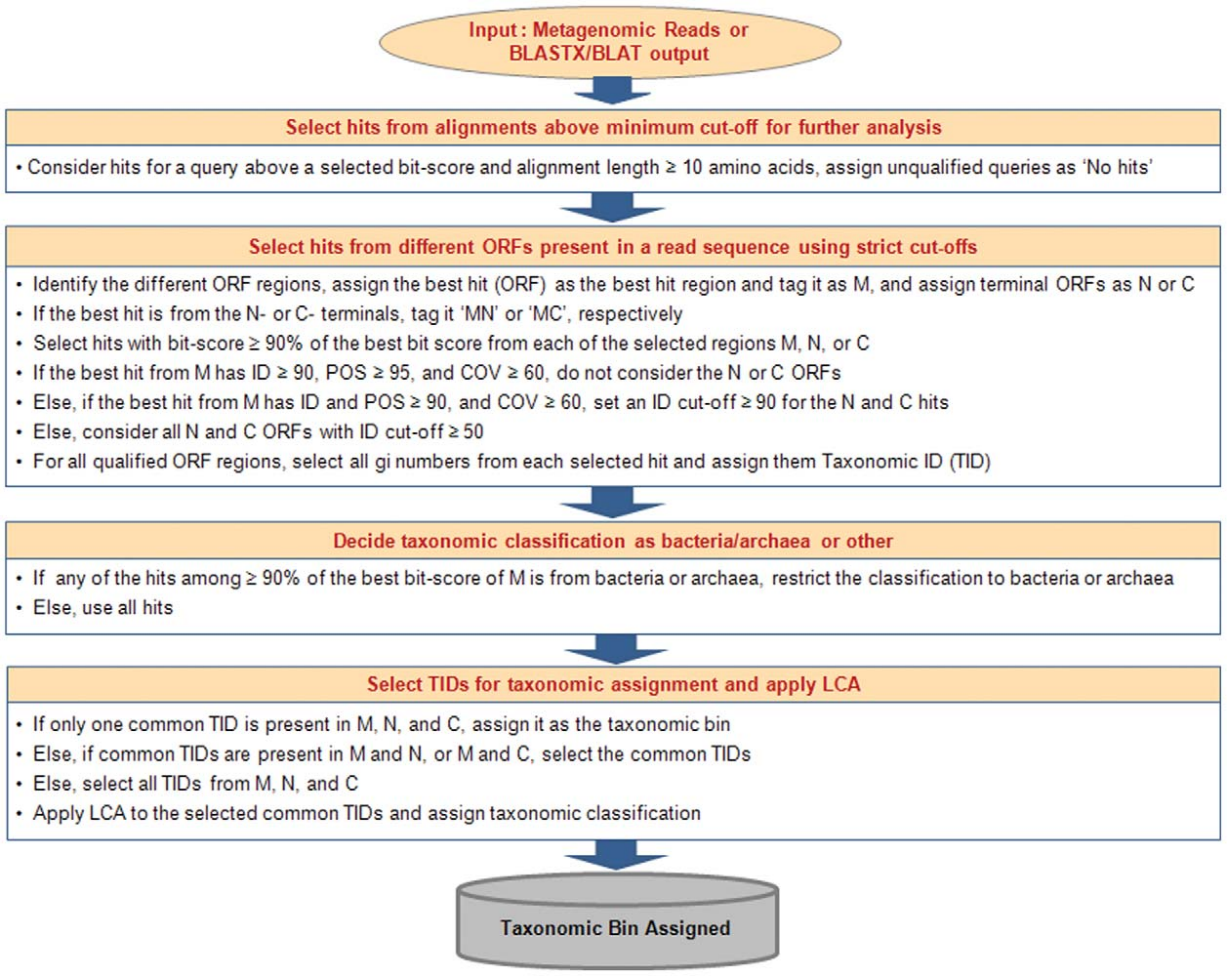
Flowchart of MetaBin algorithm. ID and POS refer to %Identity and %Positives, respectively, as provided in the Blastx or Blat output. COV refers to the % coverage of the query with the hit (reference protein).

Taking into account the taxonomic information of the common hit(s) for multiple ORFs in a read leads to more correct and specific taxonomic assignments at the genus or species level since, in most cases, only the correct hit (genome) is expected to show a match for all of the ORFs. This approach also minimizes the use of LCA analysis, which leads to non-specific or higher-level taxonomic assignment, as in the case of other programs like MEGAN. Even for reads derived from novel genomes, where the order of the ORFs in the reads may not be the same as the order of the ORFs in the reference genomes, there is a strong likelihood that the ORFs will show a match to other related genomes of the same genus or family, and thus will result in correct prediction of the taxonomic lineage by subsequent LCA analysis. After parsing the alignment output file for each read, the steps shown in Figure 2 are carried out for taxonomic assignment, and in case of paired-end read data, the steps shown in Figure S1 and as described in Text S2 are additionally carried out for re-assignment. Since the hits are qualified based on bit-score, the alignment results are sorted by bit-score because, by default, they are sorted by Expect value (E-value). In addition, we use the genome information of all the genomes listed in the annotation line of any given protein of the NCBI NR database to avoid any undesired genome bias that results from using only the first genome of the annotation line. Consideration of the above sequence features by the MetaBin algorithm results in more accurate and more specific taxonomic assignments for all different read lengths.

## Results

### Validation on simulated read datasets

To validate MetaBin on simulated metagenomic data, we carried out taxonomic analysis of the simulated read datasets using MetaBin with both Blastx and Blat output (referred to as MetaBinX and MetaBinT, respectively), and with MEGAN and SOrt-ITEMS using the Blastx output on various simulated read datasets. The assignments were counted at three levels, namely ‘Genus’, ‘Family, and ‘Phylum’ as shown in Figure S2. An assignment is counted as ‘correct’ when the assigned taxonomic level is the same as the expected taxonomic level. For example, when a read belonging to ‘Escherichia coli str. K-12 substr. DH10B’ is assigned to the phylum ‘Proteobacteria’, the assignment is considered as correct since this is the expected phylum. To calculate sensitivity and the positive predictive value (PPV), we considered only intragenic reads because the reference database (NR) contains only protein sequences, and therefore, only reads originating from protein coding regions (intragenic) are expected to find a match. We used the following standard formulae for calculating sensitivity and PPV.
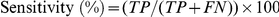


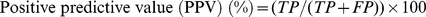


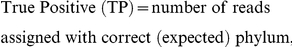


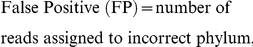


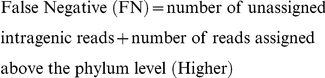
For all the bacterial read datasets, in most comparisons including longer reads (800 bp, 450 bp and 250 bp) and all comparisons including shorter reads (75 bp and 45 bp), MetaBinX assigned a higher percentage of reads to their correct genus, family and phylum, as compared to MEGAN and SOrt-ITEMS (Table 1 and Table 2, details in Text S2 and Text S3). As apparent from Table 1 and Table 2, the performance of SOrt-ITEMS was much weaker in all comparisons to MetaBinX and in most comparisons to MEGAN. Therefore, for most comparisons discussed in the following text, we have mainly compared MetaBin with MEGAN. For longer read lengths, the performance of MetaBinT was mostly comparable to that of MetaBinX, MEGAN and SOrt-ITEMS for NR, but for NRminusGenus and NRminusFamily it was slightly weaker. MetaBinX and MetaBinT both performed significantly better for short reads and assigned, up to 18% for NR, and up to 7% for NRminusGenus and up to 9% for NRminusFamily, more reads to the correct genus and phylum, respectively as compared to MEGAN. The ability of MetaBin to make more accurate assignments at the lower and more specific taxonomic levels underscores its usefulness.

**Table 1:** Summary of results using MetaBin, MEGAN and SOrt-ITEMS on simulated bacterial read datasets for different sequencing technologies.

Read Length (bp)	Method	Complete NR Database (NR)					NR with genus deleted (NRminusGenus)				NR with family deleted (NRminusFamily)		
		Genus	Family	Phylum	Sens	PPV	Family	Phylum	Sens	PPV	Phylum	Sens	PPV
**800**	**MetaBinX**	93.49	97.97	99.15	99.18	99.95	33.68	61.57	64.18	85.84	49.35	51.91	80.13
	**MetaBinT**	93.46	97.92	99.1	99.19	99.96	28.68	52.89	66.04	80.91	42.07	56.47	75.19
	**MEGAN**	92.53	97.46	98.61	98.67	99.92	33.16	60.46	63.04	85.73	49.31	51.88	79.94
	**SOrt-ITEMS**	52.62	68.29	94.61	96.01	97.7	6.25	48.01	49.38	84.59	35.68	36.89	78.42
**400**	**MetaBinX**	88.03	92.87	94.71	94.47	99.92	24.84	45.89	47.71	83.03	33.88	35.65	75.05
	**MetaBinT**	83.14	87.97	90.46	93.83	99.85	15.78	28.96	49.69	78.91	20.44	41.81	72.77
	**MEGAN**	87.73	92.72	94.49	94.28	99.88	24.32	45.16	46.89	82.92	34.69	36.34	75.81
	**SOrt-ITEMS**	34.41	67.62	91.53	91.7	98.1	8.35	42.84	45.19	78.68	32.86	35.29	68.89
**250**	**MetaBinX**	86.94	92.14	94.73	94.79	99.88	21.63	39.39	40.86	81.19	27.89	29.29	72.71
	**MetaBinT**	85.71	90.75	93.53	93.57	99.89	14.63	26.45	58.41	78.36	18.22	50.25	71.76
	**MEGAN**	86.33	91.73	94.28	94.43	99.77	21.24	38.84	40.48	81.17	28.05	29.29	73.11
	**SOrt-ITEMS**	41.63	59.8	78.06	78.31	97.5	8.12	30.18	31.41	79	22.46	23.48	69.16

Read Length <100 bp													
**75**	**MetaBinX**	92.14	95.85	96.59	98.53	99.87	20.18	35.91	53.77	77.15	25.97	42.43	66.06
	**MetaBinT**	93	96.77	97.63	99.09	99.86	16.15	29.05	51.43	67.33	22.06	43.4	55.17
	**MEGAN**	82.44	88.55	92.36	94.3	99.74	16.85	32.17	48.60	74.50	23.86	36.77	64.21
	**SOrt-ITEMS**	76.52	80.84	82.65	82.03	95.32	13.68	33.6	36.91	69.98	25.11	27.89	59.79
**45**	**MetaBinX**	76.51	80.48	82.37	96.89	99.98	7.91	12.48	53.44	72.53	6.96	37.91	63.58
	**MetaBinT**	86.12	90.56	92.93	96.47	99.70	10.83	19.02	57.07	64.97	13.23	52.5	56.08
	**MEGAN**	67.94	73.02	76.69	90.3	99.84	6.59	11.25	46.63	73.62	3.82	20.67	65.39
	**SOrt-ITEMS**	49.44	56.51	59.53	59.24	93.81	5.94	10.82	11.24	69.65	6.04	6.2	60.64

**Table 2:** Summary of results using MetaBin, MEGAN and SOrt-ITEMS on simulated archaeal read datasets for different sequencing technologies.

Read Length (bp)	Method	Complete NR Database (NR)					NR with genus deleted (NRminusGenus)				NR with family deleted (NRminusFamily)		
		Genus	Family	Phylum	Sens	PPV	Family	Phylum	Sens	PPV	Phylum	Sens	PPV
**800**	**MetaBinX**	97.81	98.44	99.69	99.69	100	28.35	77.96	80.33	95.95	56.85	59.8	81.18
	**MetaBinT**	97.86	98.65	99.58	99.63	99.95	21.63	64.72	80.49	93.99	48.15	63.58	79.14
	**MEGAN**	97.86	98.54	99.64	99.63	100	28.09	77.75	80.61	95.16	56.54	59.97	78.75
	**SOrt-ITEMS**	60.24	75.09	99.11	99.11	100	2.24	60.03	61.23	96.65	42.78	44	85.21
**400**	**MetaBinX**	92.09	93.19	94.95	95.07	99.87	19.23	58.66	59.85	95.92	42.05	42.98	79.26
	**MetaBinT**	87.19	88.39	90.94	94.9	99.92	9.69	33.17	65.59	93.13	24.64	51.51	77.27
	**MEGAN**	92.34	93.65	95.48	95.75	99.81	19.85	59.38	61.38	95.37	42.25	43.91	78.81
	**SOrt-ITEMS**	38.86	73.82	95.23	95.79	99.39	5.76	53.22	55.67	91.84	37.14	39.21	73.57
**250**	**MetaBinX**	91.54	92.75	95.16	95.19	99.96	15.65	47.87	49.12	94.32	34.32	35.33	78.19
	**MetaBinT**	90.74	91.97	94.48	98.39	99.94	8.1	26.78	73.57	92.15	19.69	57.81	75.69
	**MEGAN**	91.86	93.31	95.71	95.9	99.85	16.47	49.96	51.91	93.1	35.43	36.88	75.49
	**SOrt-ITEMS**	52.54	73.85	95.3	95.47	99.81	5.09	34.84	36.34	88.95	23.8	24.81	72.19

Read Length <100 bp													
**75**	**MetaBinX**	96.7	96.95	97.29	99.2	99.96	12.51	41.77	67.24	90.91	30.47	50.54	72.95
	**MetaBinT**	96.85	97.14	97.51	99.59	99.93	7.98	27.25	57.43	75.56	20.28	43.48	59.37
	**MEGAN**	89.3	91.39	96.26	98.23	99.89	11.95	40.83	66.61	89.7	29.62	49.47	70.3
	**SOrt-ITEMS**	94.07	95.21	96.4	96.54	99.85	8.15	34.11	36.49	83.73	23.73	25.6	66.07
**45**	**MetaBinX**	82.4	83.07	84.77	98.95	99.99	2.53	8.96	62.02	86.48	6.56	47.04	70.17
	**MetaBinT**	92.99	93.9	95.84	98.73	99.92	3.97	14.29	75.23	75.23	10.68	51.23	58.61
	**MEGAN**	74.19	76.35	81.96	95.71	99.96	2.57	9.59	64.46	90.73	7.03	49.07	73.72
	**SOrt-ITEMS**	56.07	60.79	64.03	64.33	99.25	2.27	7.6	7.71	85.39	5.33	5.44	70.65

The above tables show the percentage of total reads correctly assigned at different taxonomic levels such as Genus, Family or Phylum. ‘Sens’ refers to %average sensitivity and ‘PPV’ refers to %average positive predictive value.

The average sensitivity and PPV of MetaBinX for all simulated read datasets was also similar or higher as compared to MEGAN and SOrt-ITEMS, especially for short reads. MetaBinT showed comparable sensitivity and PPV for NR, but its PPV was lower for NRminusGenus and NRminusFamily, though the sensitivity was considerably higher. This could be attributed to the differences in the accuracy and sensitivity of the Blastx and Blat algorithms. In the case of simulated reads derived from the bacterial genomes, for ∼75 bp reads, both MetaBinT and MetaBinX showed 2.8–6.6% higher average sensitivity for NR, NRminusGenus and NRminusFamily as compared to MEGAN, and showed 14.5–16.8% higher average sensitivity for NR, NRminusGenus and NRminusFamily as compared to SOrt-ITEMS. In the case of ∼45 bp reads, for NR both MetaBinT and MetaBinX showed >6% higher average sensitivity, for NRminusGenus, MetaBinT showed 10.4% and MetaBinX showed ∼7% higher sensitivity, and for NRminusFamily, MetaBinT showed ∼32% and MetaBinX showed 17% higher sensitivity as compared to MEGAN. For the same read length (∼45 bp), both MetaBinT and MetaBinX showed 31–46% higher average sensitivity, for NR, NRminusGenus, and NRminusFamily, as compared to SOrt-ITEMS.

In the case of simulated reads derived from the archaeal genomes, for longer reads, in the case of NR, all tools showed comparable sensitivity for all comparisons. However, for NRminusGenus and NRminusFamily, the performance of MetaBinX, MetaBinT and MEGAN was comparable, whereas SOrt-ITEMS showed a much lower sensitivity. Similarly, for ∼75 bp reads, the sensitivity of MetaBinX, MetaBinT and MEGAN was mostly comparable. For ∼45 bp reads, MetaBinT showed much greater sensitivity and assigned a greater number of reads to their correct genus, family or phylum. Overall, among the tools used, the performance of SOrt-ITEMS was considerably weaker as compared to MetaBin and MEGAN.

### Validation on real metagenomic dataset (short reads): Gut metagenomic data from a European individual

To validate MetaBin on real metagenomic data, we used recent human gut data obtained by Illumina sequencing from a Spanish male individual (V1CD2) [15], and analyzed it using MetaBin with Blat as the alignment program. The ‘prepareinput’ program was used to translate the reads into six reading frames and align them against NR using Blat which generated a large size output file of 149 GB. MetaBin (the ‘metabin’ program) was then run to carry out the taxonomic assignments, and only those bins containing at least 10,000 reads were considered, while the rest of the parameters used the default values. In total, it took about 370 CPU hours, which is really reasonable considering the input size of more than 20.72 million reads. A large number of reads, 12,840,080 (62%), had no match in the NR database. One reason for this could be that some of these reads (∼12%) are derived from the intergenic regions, as in case of simulated reads of a similar length (∼75 bp) (Figure S3 and Figure S4). Other possible reasons could be short read length, the strict classification criteria used by MetaBin, and the fact that a large number of genomes present in the human gut are novel and not well represented in the NR database [16].

A total of 7,019,398 (33.9%) reads could be assigned to taxonomic bins, and the remaining 865,736 (4.18%) reads could not be assigned to any taxonomic bin (Table S2). Bacteroidetes was the most abundant phylum (77.4%) followed by Firmicutes (16.8%), Proteobacteria (3.5%), Actinobacteria (1.7%), Cyanobacteria (0.27%), and Euryarchaeota (0.24%). These results corroborate previous observations [15], [16]. Since this data was available as paired-end reads, we analyzed the same dataset using the paired-end option of MetaBin. Out of 10,348,691 read pairs, for 3,714,756 (35.9%) read pairs (both reads) remained either unassigned or had no Blat hits, and for 6,249,660 (60.4%), only one of the reads could be assigned to a taxonomic bin, while the other read remained unassigned or had no Blat hit, as might be expected for reads derived from the intergenic regions (Figure 1 and Figure S3). For the latter read pairs, the taxonomic bin of the assigned read was directly allocated as the taxonomic bin of the unassigned read. Of the remaining 384,274 read pairs where both the reads were taxonomically assigned, 355,015 (92.4%) read pairs were assigned to the same lineage and 29,259 (7.6%) read pairs were assigned to different lineages. Of the latter, 10,121 read pairs were reassigned to the same lineage, while the remaining 19,138 read pairs could not be reassigned since there was no apparent taxonomic similarity between the reads.

### Validation on real metagenomic dataset (long reads): Sargasso sea dataset

To test the performance on longer reads, we compared the results of MetaBin, MEGAN and SOrt-ITEMS using the same sample data obtained from Sargasso Sea dataset which was used in previous studies [7], [8]. MetaBin and MEGAN both predicted a similar number of bins at the phylum, family and genus levels which corroborate with the previous study on the entire Sargasso sea dataset [17]. Furthermore, MetaBin assigned comparatively more reads (nearly twice the number of reads at the species level) to each of these common bins (Table S3), which shows its ability to assign more reads (higher sensitivity) (details are provided in Text S2). Overall, the performance of SOrt-ITEMS was comparatively limited compared to both MetaBin and MEGAN. Though the focus of this study is on homology-based approaches, to provide a brief comparison of MetaBin with two publicly available composition-based methods (TACOA and NBC), as well as with another method based on homology to protein families (WebCARMA), we compared the performance of these programs on the same dataset. As apparent from the results, the composition-based (TACOA) and protein family based (WebCARMA) method have limitations for making comprehensive taxonomic assignments as compared to homology-based methods. However, another composition-based method, NBC, showed unusually high assignments as it assigned almost all the reads to the phylum or even to the genus level, which is surprising since these sample reads are derived from a metagenomic environment (Sargasso sea) where a large number of genomes are novel (yet uncultured and not yet sequenced). Therefore, an almost absolute taxonomic assignment at the genus, or even at the phylum level, is certainly not expected with the current knowledge.

### Comparative analysis using human gut datasets

To demonstrate the comparative analysis feature of MetaBin, we used two human gut datasets, HGF1S and HGF1T, and analyzed the Blastx results for both using MetaBin. In the taxonomic tree dendrogram shown in Figure S5, the HGF1S and HGF1T datasets are represented in red and blue, respectively. When a taxonomic bin is commonly present in both datasets, its respective normalized proportions are shown as a pie chart with the above assigned colors. Since HGF1S and HGF1T are from individuals belonging to the same family, it is apparent that their guts contain similar flora, but have surprisingly different amounts of the constituent microbes (Figure S5 and Table S4). All phyla were common to both datasets, but ‘Bacteroidetes’ was the most abundant phylum for HGF1S, whereas ‘Firmicutes’ was the most abundant phylum for HGF1T which corroborate with the previous study on the entire human gut dataset [16]. We could also analyze this data for taxonomic reassignment using the paired-end option, and the results are described in Text S2.

### Features of the web server

The web server provides several options (described below) for taxonomic analysis, visualization of results, and comparative analysis.

### Application - Taxonomic analysis of metagenomic data

Using this page, the user can submit and carry out taxonomic analysis of either sequence reads or Blastx output. Since MetaBin uses a homology-based approach, alignments with a reference database (NCBI NR) are required. Therefore, the ‘Application’ page presents two options, BLAT and BLAST, to generate the alignments. The first option, BLAT, uses Blat as the alignment method and is much faster (up to 1000 times) as compared to Blastx, and thus can dramatically reduce the amount of time taken to generate the alignments with comparable results (Table 1 and 2, Figure S6). When submitting sequence reads as input, we recommend the Blat option to obtain faster results and the complete process, including alignment and taxonomic classification, can be carried out at the server. The input sequences submitted in FASTA format is first checked for the correct input format (refer to Tutorial available on the website for details). After validation, reading frames (RFs) are predicted in the reads by translating them into six reading frames, and the qualified RFs (≥10 amino acids) are aligned against the NCBI NR database using Blat. The alignment results are then analyzed to classify the sequences into their appropriate taxonomic bins. The second option, BLAST, uses Blastx for generating the alignments. Here we recommend users to run the Blastx job (full alignment format) on their own machine, preferably using multiple node/processors, and then upload the Blastx alignment output to our server for carrying out the taxonomic assignments. An option to upload sequence reads is also provided, but it will take a much longer time to generate the alignments as Blastx is very slow in comparison to Blat.

Various options are available to change the input parameters such as minimum bit-score (Blat or Blastx output), bit-score range to select hits, and bin size (minimum number of reads needed to form a taxonomic bin), otherwise the default parameters will be used. Additionally, the user can select either the complete NR database or the ‘NR minus Eukaryotes’ version from which all the proteins belonging exclusively to eukaryotes are deleted. Since the focus of metagenomic studies is often to determine the prokaryotic composition, the latter option is useful and decreases the total analysis time by about 30%. An option to specify if the reads should be analyzed as paired-ends is also available. The ‘Results’ page provides the output files in tab-delimited format which can be downloaded from the server. It displays thumbnail images of the taxonomic tree (*.png file) and functional annotation of the reads using COGs functional classes which can be clicked on to view the full-sized images. A summary table of the reads assigned to each of the taxonomic bins (*.sum file) is also shown.

### Visualization of results and comparative analysis

The Results page provides a link to the ‘Visualization’ page, where several options for displaying the results and carrying out comparative analysis can be found. If the MetaBin standalone version was used, the resultant *.json file can be uploaded on this page for additional web-based analyses. The first option ‘Create Taxonomic Tree’ is used to visualize the taxonomic tree and prepare a ‘Composition chart’ for a single dataset. On the taxonomic tree, each taxonomic bin is shown as a node whose size is determined by the proportion of the number of reads assigned to that bin to the total number of reads in that dataset. The composition chart provides an overview of the microbial distribution in the dataset and shows the ‘abundance of the microbes’, which is computed as a proportion of the total number of reads assigned to any taxonomic bin of a certain taxonomic level by the total number of reads assigned to that taxonomic level. The second option ‘Compare Metagenome Profiles’ can be used to compare the taxonomic profiles of up to five metagenomic datasets using the *.json output files from the MetaBin analysis. The taxonomic tree generated after comparing the metagenomic profiles of multiple datasets shows each dataset as distinct colors; and, when a taxonomic bin is present in two or more datasets, its respective normalized proportions are shown as a pie chart using the same color scheme. The composition chart compares the microbial distribution in the datasets and shows the ‘abundance of microbes’ classified at various taxonomic levels in the different datasets represented in different colors.

### Stand-alone version of MetaBin

To analyze large metagenomic datasets, a free stand-alone executable program is available for download for several operating systems including Linux, Mac, and Windows. Various options are available to change the input parameters such as bin size (‘-b’, minimum number of reads needed to form a taxonomic bin), minimum bit-score (‘-s’, Blastx or Blat output) and bit-score range (‘-r’), to select hits. An option (-d) is provided for comparative analysis which generates a taxonomic tree after comparing the proportions of each taxonomic group in the selected metagenomes, and displays the respective proportions as a pie chart. Using the ‘-p’ option, the program can also be used for the taxonomic assignments of paired-end sequence read data, accepted in the specified formats (refer to the website Tutorial for details). To use Blat as the alignment method, the user should run the ‘prepareinput’ program to translate the reads into six reading frames and run Blat. The output of this program is used as the input for the ‘metabin’ program. For Blastx, users should carry out the alignments separately by generating an output in full alignment format and then use the Blastx output as input for the ‘metabin’ program.

## Discussion

The correct assignment of metagenomic sequences to their respective source genomes, or taxonomic lineage, is a critical step for estimating the complexity of any metagenome and for further functional analysis of the metagenomic data [18]. Though the homology-based approaches are more common, specific, and useful for diverse length of reads as compared to the composition-based approaches, their implementation on large metagenomic datasets is dramatically limited by the long analysis time needed to generate the Blastx alignments. MetaBin provides a significant improvement over the currently existing homology-based methods for better and faster taxonomic assignments by using a more specific ORF-based approach. Taking into account the taxonomic information from common hit(s) for multiple ORFs in a read leads to more correct and specific taxonomic assignments at the genus and species level, and minimizes the use of LCA analysis, which leads to non-specific or higher level taxonomic assignment, as in the case of other programs.

The implementation of Blat in MetaBin makes it practical to use a more accurate and sensitive homology-based approach for the high-throughput analysis of large datasets and for the development of a web-based community server. It allows the user to directly submit their reads on our server and run the complete analysis pipeline, including both homology-based alignment and taxonomic assignment. The web server also provides several useful options for visualization of results and comparative analysis of multiple metagenomic datasets. In addition, the functional analysis of reads using COGs provides insights on the functional composition of the data. The availability of a standalone command line version of the program allows users to carry out large-scale analysis on their own machines, and also makes it suitable for integration in other pipelines for automated analysis of metagenomic data.

The results obtained from the analysis of simulated reads and a variety of real metagenomic datasets attests to the usability, accuracy, and sensitivity of MetaBin. Further, its ability to perform a complete analysis, including alignment with NR and taxonomic assignment, for such large datasets like the EU individual human gut metagenomic data [15], consisting of short ∼75 bp Illumina reads, in relatively short time, clearly demonstrates the practical usability of MetaBin on real metagenomic data. MetaBin can be used for the comprehensive taxonomic assignment of sequence reads of diverse lengths (≥45 bp) derived from any existing sequencing technology. To our knowledge, it is the only method which can be applied for the taxonomic binning of reads of lengths as short as 45–75 bp with higher accuracy and sensitivity than competing methods, as demonstrated in this work.

In conclusion, the MetaBin program and web server can be considered a significant improvement over currently existing programs for carrying out the taxonomic binning of metagenomic sequences with high accuracy, sensitivity and speed. In the future, we plan to further improve its performance, to keep it updated as per the advancements in next-generation sequencing technologies, and to continue the development of more options for comparative analysis and visualization of the results.

## Supporting Information

Figure S1 Calculation of weight and criteria for reassigning taxonomic bin to paired-end reads on the basis of weight. The abbreviations ID and POS refer to %Identity and %Positives, respectively, as provided in Blastx output. COV refers to the % coverage of the query with the hit (reference protein).
(TIF)

Figure S2 Schematic view of taxonomic classification for calculation of sensitivity and PPV.
(DOC)

Figure S3 Number of simulated reads originating from intergenic regions for various sequencing methodologies. As expected the chances for a read to have originated from an intergenic region increases as the read length decreases or as the intergenic distance increases (Figure S4).
(DOC)

Figure S4 Summary of average gene length (blue) and average intergenic distance (red) for the 25 bacterial and two archaeal genomes. The average intergenic regions are small (∼182 bp on average) in the selected microbial genomes. In the case of CFP2 and RSD17, the average intergenic distances are longer as compared to the other genomes. A plausible explanation could be that because both of these bacteria are endosymbionts, many of their functional genes have become pseudogenes, thus converting genes into intergenic regions. The average gene length was ∼956 bp.
(DOC)

Figure S5 Comparison of the gut microflora of HGF1S and HGF1T datasets. The HGF1S and HGF1T datasets are represented in red and blue, respectively. When a taxonomic bin is commonly present in both datasets, its respective normalized proportions are shown as a pie chart with the above assigned colors.
(TIF)

Figure S6 Comparison of time taken for processing the BLASTX results for different numbers of reads by MetaBin and MEGAN. Simulated and real metagenomic reads of length ∼800 bp (Sanger) were used. The approximate size of the datasets containing 1,000, 5,000, 10,000, 15,000, and 20,000 reads were 0.34, 1.9, 4.8, 6.4, and 8.3 GB, respectively. MetaBin is comparatively much faster than MEGAN in processing the Blastx output and carrying out the taxonomic analysis.
(DOC)

Table S1 Complete taxonomic lineage of 27 microbial (bacteria and archaea) genomes used in this analysis.
(DOC)

Table S2 Summary of taxonomic assignment of reads by MetaBin using Blat (MetaBinT) for the gut metagenomic data from a European individual (V1CD2).
(DOC)

Table S3 Comparative analysis of taxonomic assignment of reads by homology- and composition-based methods for the Sargasso dataset (SSea Sample 1).
(DOC)

Table S4 Comparative analysis of human gut datasets HGF1T and HGF1S.
(DOC)

Text S1 Description of possible cases (A–G as shown in Figure 1).
(DOC)

Text S2 Supplementary information.
(DOC)

Text S3 Genome wise summary of analysis by MetaBinX and MetaBinT on 25 simulated read datasets.
(DOC)
