# Supplementary figures and images for: Fast and Accurate Taxonomic Assignments of Metagenomic Sequences Using MetaBin
Source: PLoS One. 2012 Apr 4;7(4):e34030. doi: 10.1371/journal.pone.0034030 (PMC3319535; doi:10.1371/journal.pone.0034030)

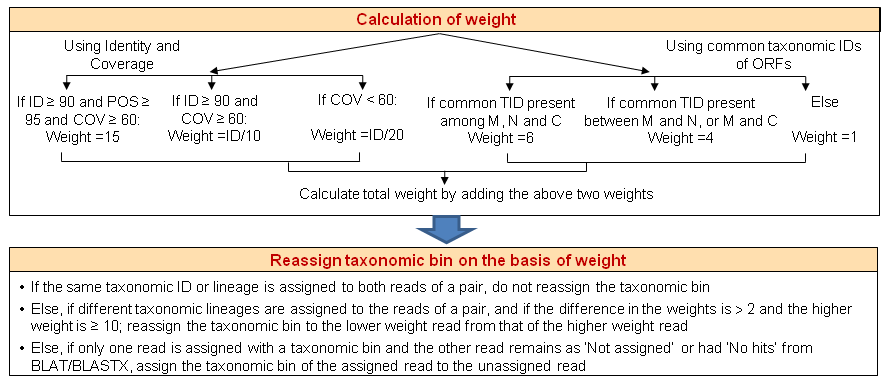

Supplement: Figure S1 — Calculation of weight and criteria for reassigning taxonomic bin to paired-end reads on the basis of weight. The abbreviations ID and POS refer to %Identity and %Positives, respectively, as provided in Blastx output. COV refers to the % coverage of the query with the hit (reference protein). (TIF) [file pone.0034030.s001.tif]

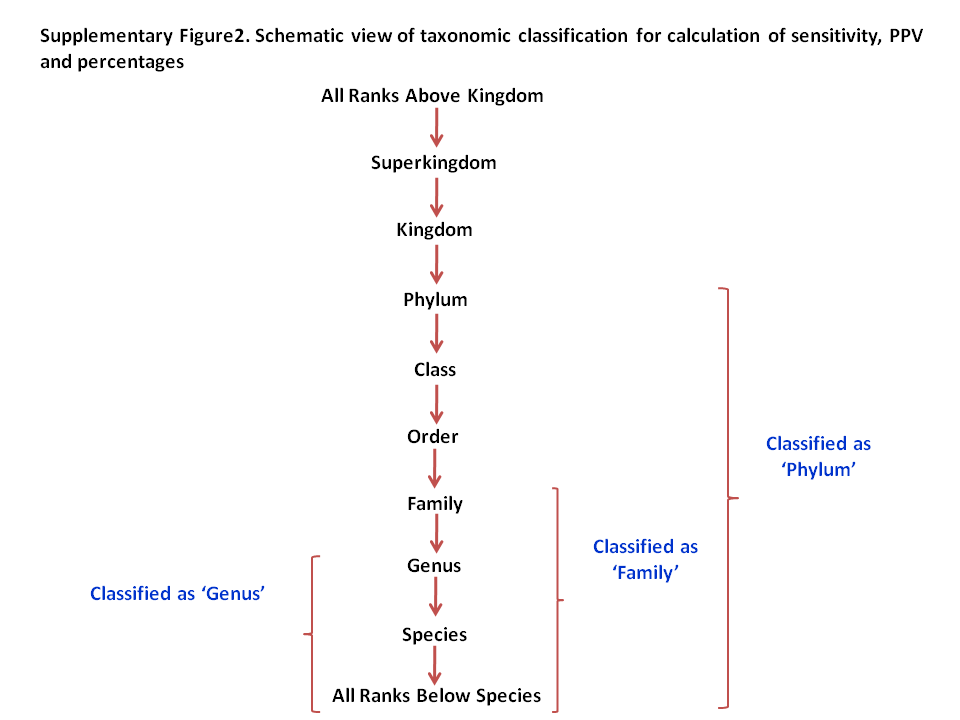

Supplement: Figure S2 — Schematic view of taxonomic classification for calculation of sensitivity and PPV. (DOC) [file pone.0034030.s002.doc]

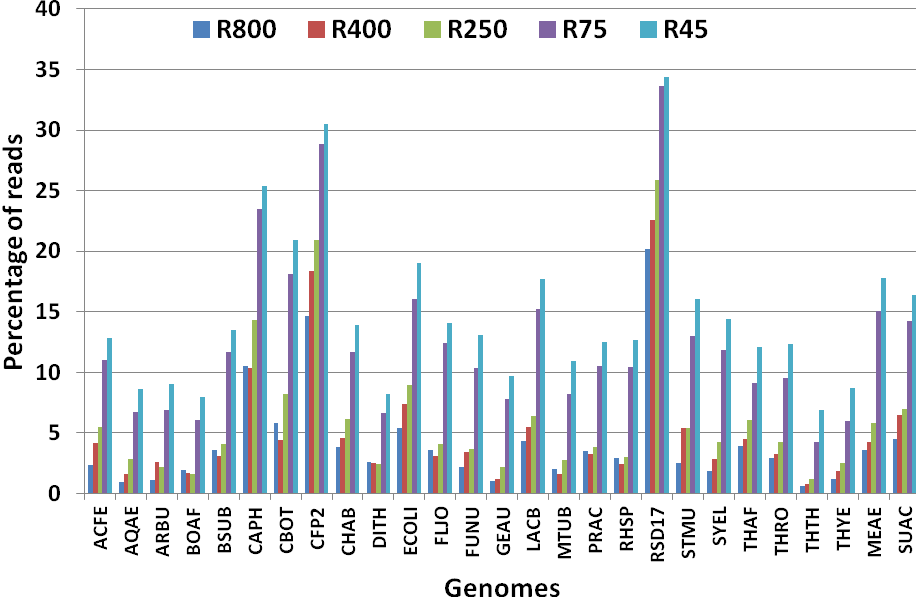

Supplement: Figure S3 — Number of simulated reads originating from intergenic regions for various sequencing methodologies. As expected the chances for a read to have originated from an intergenic region increases as the read length decreases or as the intergenic distance increases (Figure S4). (DOC) [file pone.0034030.s003.doc]

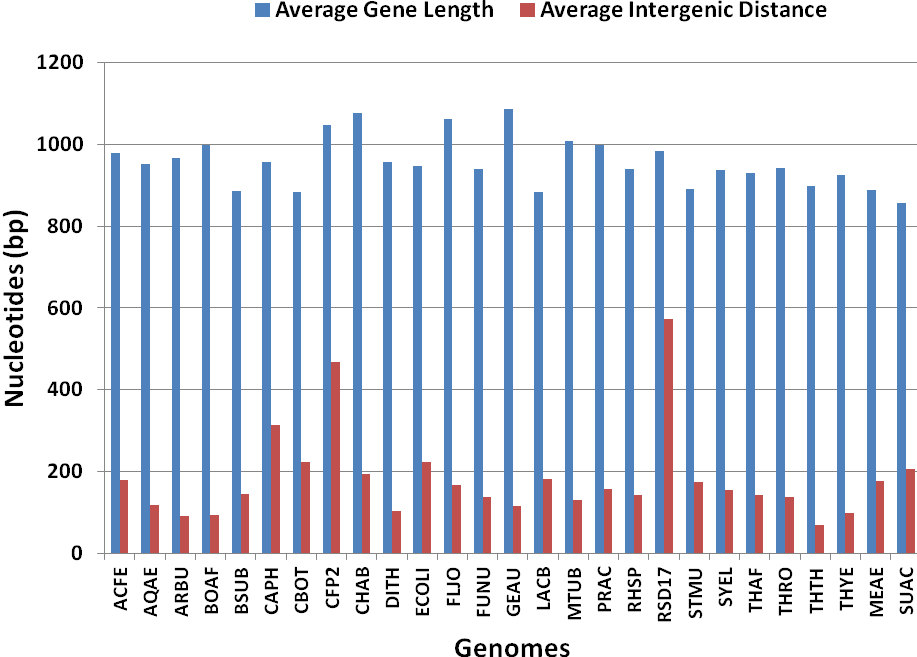

Supplement: Figure S4 — Summary of average gene length (blue) and average intergenic distance (red) for the 25 bacterial and two archaeal genomes. The average intergenic regions are small (∼182 bp on average) in the selected microbial genomes. In the case of CFP2 and RSD17, the average intergenic distances are longer as compared to the other genomes. A plausible explanation could be that because both of these bacteria are endosymbionts, many of their functional genes have become pseudogenes, thus converting genes into intergenic regions. The average gene length was ∼956 bp. (DOC) [file pone.0034030.s004.doc]

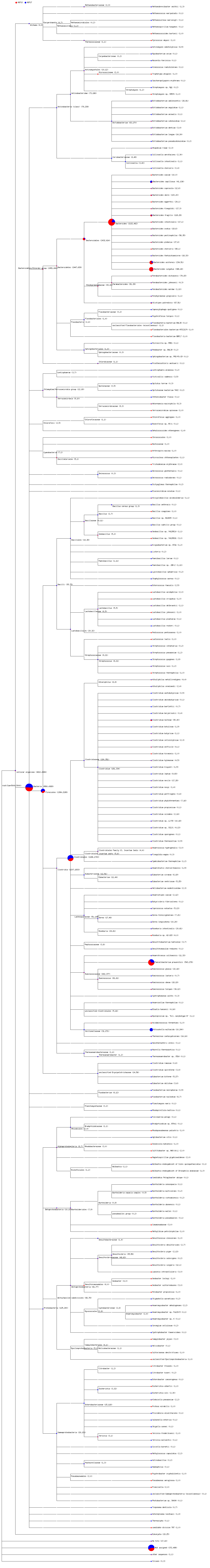

Supplement: Figure S5 — Comparison of the gut microflora of HGF1S and HGF1T datasets. The HGF1S and HGF1T datasets are represented in red and blue, respectively. When a taxonomic bin is commonly present in both datasets, its respective normalized proportions are shown as a pie chart with the above assigned colors. (TIF) [file pone.0034030.s005.tif]

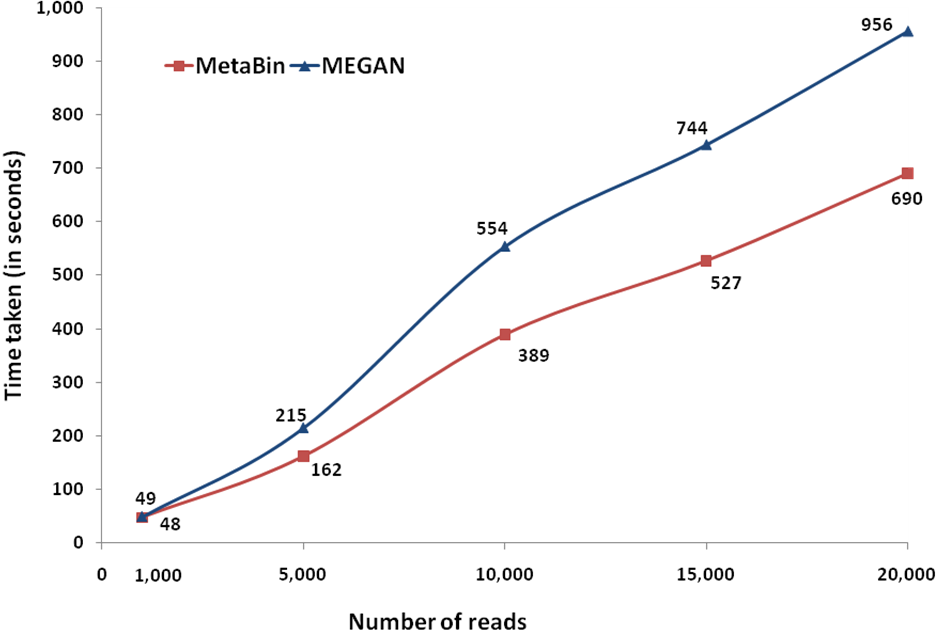

Supplement: Figure S6 — Comparison of time taken for processing the BLASTX results for different numbers of reads by MetaBin and MEGAN. Simulated and real metagenomic reads of length ∼800 bp (Sanger) were used. The approximate size of the datasets containing 1,000, 5,000, 10,000, 15,000, and 20,000 reads were 0.34, 1.9, 4.8, 6.4, and 8.3 GB, respectively. MetaBin is comparatively much faster than MEGAN in processing the Blastx output and carrying out the taxonomic analysis. (DOC) [file pone.0034030.s006.doc]
