# Supplementary material for: Fast and Accurate Taxonomic Assignments of Metagenomic Sequences Using MetaBin
Source: PLoS One. 2012 Apr 4;7(4):e34030. doi: 10.1371/journal.pone.0034030 (PMC3319535; doi:10.1371/journal.pone.0034030)
Supplement: Table S1 — Complete taxonomic lineage of 27 microbial (bacteria and archaea) genomes used in this analysis. (DOC) [file pone.0034030.s007.doc]

**Table S1** Complete taxonomic lineage of 27 microbial (bacteria and archaea) genomes used in this analysis

| **Bacterial genomes** | | | |
| --- | --- | --- | --- |
| **Genome Name** | **Symbol^1^** | **Taxonomic ID^2^** | **Complete Taxonomic Lineage** |
| Acidimicrobium ferrooxidans DSM 10331 | ACFE | 525909 | cellular organisms; Bacteria; Actinobacteria; Actinobacteria (class); Acidimicrobidae; Acidimicrobiales; Acidimicrobineae; Acidimicrobiaceae; Acidimicrobium; Acidimicrobium ferrooxidans |
| Aquifex aeolicus VF5 | AQAE | 224324 | cellular organisms; Bacteria; Aquificae; Aquificae (class); Aquificales; Aquificaceae; Aquifex; Aquifex aeolicus |
| Arcobacter butzleri RM4018 | ARBU | 367737 | cellular organisms; Bacteria; Proteobacteria; delta/epsilon subdivisions; Epsilonproteobacteria; Campylobacterales; Campylobacteraceae; Arcobacter; Arcobacter butzleri |
| Bacillus subtilis | BSUB | 1423 | cellular organisms; Bacteria; Firmicutes; Bacilli; Bacillales; Bacillaceae; Bacillus; Bacillus subtilis group |
| Borrelia afzelii PKo | BOAF | 390236 | cellular organisms; Bacteria; Spirochaetes; Spirochaetes (class); Spirochaetales; Spirochaetaceae; Borrelia; Borrelia burgdorferi group; Borrelia afzelii |
| Candidatus Azobacteroides pseudotrichonymphae genomovar. CFP2 | CFP2 | 511995 | cellular organisms; Bacteria; Bacteroidetes/Chlorobi group; Bacteroidetes; Bacteroidia; Bacteroidales; unclassified Bacteroidales; Candidatus Azobacteroides; Candidatus Azobacteroides pseudotrichonymphae |
| Candidatus Phytoplasma mali | CAPH | 37692 | cellular organisms; Bacteria; Tenericutes; Mollicutes; Acholeplasmatales; Acholeplasmataceae; Candidatus Phytoplasma; 16SrX (Apple proliferation group) |
| Chlamydophila abortus S26/3 | CHAB | 218497 | cellular organisms; Bacteria; Chlamydiae/Verrucomicrobia group; Chlamydiae; Chlamydiae (class); Chlamydiales; Chlamydiaceae; Chlamydophila; Chlamydophila abortus |
| Clostridium botulinum A str. ATCC 19397 | CBOT | 441770 | cellular organisms; Bacteria; Firmicutes; Clostridia; Clostridiales; Clostridiaceae; Clostridium; Clostridium botulinum; Clostridium botulinum A |
| Dictyoglomus thermophilum H-6-12 | DITH | 309799 | cellular organisms; Bacteria; Dictyoglomi; Dictyoglomia; Dictyoglomales; Dictyoglomaceae; Dictyoglomus; Dictyoglomus thermophilum |
| Escherichia coli str. K-12 substr. DH10B | ECOLI | 316385 | cellular organisms; Bacteria; Proteobacteria; Gammaproteobacteria; Enterobacteriales; Enterobacteriaceae; Escherichia; Escherichia coli; Escherichia coli K-12 |
| Flavobacterium johnsoniae UW101 | FLJO | 376686 | cellular organisms; Bacteria; Bacteroidetes/Chlorobi group; Bacteroidetes; Flavobacteria; Flavobacteriales; Flavobacteriaceae; Flavobacterium; Flavobacterium johnsoniae |
| Fusobacterium nucleatum subsp. nucleatum ATCC 25586 | FUNU | 190304 | cellular organisms; Bacteria; Fusobacteria; Fusobacteria (class); Fusobacteriales; Fusobacteriaceae; Fusobacterium; Fusobacterium nucleatum; Fusobacterium nucleatum subsp. nucleatum |
| Gemmatimonas aurantiaca T-27 | GEAU | 379066 | cellular organisms; Bacteria; Gemmatimonadetes; Gemmatimonadetes (class); Gemmatimonadales; Gemmatimonadaceae; Gemmatimonas; Gemmatimonas aurantiaca |
| Lactobacillus brevis ATCC 367 | LACB | 387344 | cellular organisms; Bacteria; Firmicutes; Bacilli; Lactobacillales; Lactobacillaceae; Lactobacillus; Lactobacillus brevis |
| Mycobacterium tuberculosis H37Rv | MTUB | 83332 | cellular organisms; Bacteria; Actinobacteria; Actinobacteria (class); Actinobacteridae; Actinomycetales; Corynebacterineae; Mycobacteriaceae; Mycobacterium; Mycobacterium tuberculosis complex; Mycobacterium tuberculosis |
| Propionibacterium acnes KPA171202 | PRAC | 267747 | cellular organisms; Bacteria; Actinobacteria; Actinobacteria (class); Actinobacteridae; Actinomycetales; Propionibacterineae; Propionibacteriaceae; Propionibacterium; Propionibacterium acnes |
| Rhodobacter sphaeroides 2.4.1 (CHROMOSOME1) | RHSP | 272943 | cellular organisms; Bacteria; Proteobacteria; Alphaproteobacteria; Rhodobacterales; Rhodobacteraceae; Rhodobacter; Rhodobacter sphaeroides |
| Streptococcus mutans UA159 | STMU | 210007 | cellular organisms; Bacteria; Firmicutes; Bacilli; Lactobacillales; Streptococcaceae; Streptococcus; Streptococcus mutans |
| Synechococcus elongatus PCC 6301 | SYEL | 269084 | cellular organisms; Bacteria; Cyanobacteria; Chroococcales; Synechococcus; Synechococcus elongatus |
| Thermodesulfovibrio yellowstonii DSM 11347 | THYE | 289376 | cellular organisms; Bacteria; Nitrospirae; Nitrospira (class); Nitrospirales; Nitrospiraceae; Thermodesulfovibrio; Thermodesulfovibrio yellowstonii |
| Thermomicrobium roseum DSM 5159 | THRO | 309801 | cellular organisms; Bacteria; Chloroflexi; Thermomicrobia (class); Thermomicrobiales; Thermomicrobiaceae; Thermomicrobium; Thermomicrobium roseum |
| Thermosipho africanus TCF52B | THAF | 484019 | cellular organisms; Bacteria; Thermotogae; Thermotogae (class); Thermotogales; Thermotogaceae; Thermosipho; Thermosipho africanus |
| Thermus thermophilus HB8 | THTH | 300852 | cellular organisms; Bacteria; Deinococcus-Thermus; Deinococci; Thermales; Thermaceae; Thermus; Thermus thermophilus |
| uncultured Termite group 1 bacterium phylotype Rs-D17 | RSD17 | 471821 | cellular organisms; Bacteria; Elusimicrobia; environmental samples; uncultured Termite group 1 bacterium |
| **Archaeal genomes** | | | |
| Methanococcus aeolicus Nankai-3 | MEAE | 419665 | cellular organisms; Archaea; Euryarchaeota; Methanococci; Methanococcales; Methanococcaceae; Methanococcus; Methanococcus aeolicus |
| Sulfolobus acidocaldarius DSM 639 | SUAC | 330779 | cellular organisms; Archaea; Crenarchaeota; Thermoprotei; Sulfolobales; Sulfolobaceae; Sulfolobus; Sulfolobus acidocaldarius |

^1^: Symbol used in this analysis for the respective genome

^2^: Taxonomic ID as per the NCBI taxonomy database (http://www.ncbi.nlm.nih.gov/Taxonomy/)
