# Supplementary material for: Fast and Accurate Taxonomic Assignments of Metagenomic Sequences Using MetaBin
Source: PLoS One. 2012 Apr 4;7(4):e34030. doi: 10.1371/journal.pone.0034030 (PMC3319535; doi:10.1371/journal.pone.0034030)
Supplement: Table S2 — Summary of taxonomic assignment of reads by MetaBin using Blat (MetaBinT) for the gut metagenomic data from a European individual (V1CD2). (DOC) [file pone.0034030.s008.doc]

**Table S2** Summary of taxonomic assignment of reads by MetaBin (without paired-end option) using Blat (MetaBinT) for the gut metagenomic data from a European individual (V1CD2)

2a) Summary of reads assigned to various taxonomic levels

| Taxonomy | Number of assigned reads | % of total assignments at that level |
| --- | --- | --- |
| Phylum | 6,566,641 | 93.55 |
| Order | 6,277,417 | 89.43 |
| Class | 6,386,096 | 90.98 |
| Family | 5,546,786 | 79.02 |
| Genus | 5,388,543 | 76.77 |
| Species | 4,115,181 | 58.63 |
| Total assigned | 7,019,398 |  |

2b) Reads assigned to the phylum level

| Taxonomy | Number of assigned reads | % of total assignments at phylum level |
| --- | --- | --- |
| Bacteroidetes | 5,081,997 | 77.39 |
| Firmicutes | 1,106,403 | 16.85 |
| Proteobacteria | 232,886 | 3.55 |
| Actinobacteria | 112,266 | 1.71 |
| Cyanobacteria | 17,412 | 0.27 |
| Euryarchaeota | 15,677 | 0.24 |

At the phylum level, six phyla were predicted. Among these, Bacteroidetes and Firmicutes are the most abundant phyla containing ≥94% of the total assigned reads.

2c) Reads assigned to the family level

| Taxonomy | Number of assigned reads | % of total assignments at family level |
| --- | --- | --- |
| Prevotellaceae | 3,419,292 | 61.64 |
| Bacteroidaceae | 1,133,762 | 20.44 |
| Ruminococcaceae | 269,992 | 4.87 |
| Eubacteriaceae | 169,222 | 3.05 |
| Lachnospiraceae | 118,705 | 2.14 |
| Veillonellaceae | 100,005 | 1.80 |
| Clostridiaceae | 96,463 | 1.74 |
| Rikenellaceae | 42,618 | 0.77 |
| Porphyromonadaceae | 32,752 | 0.59 |
| Erysipelotrichaceae | 27,761 | 0.50 |
| Streptomycetaceae | 26,676 | 0.48 |
| Burkholderiaceae | 21,054 | 0.38 |
| Enterobacteriaceae | 19,002 | 0.34 |
| Streptococcaceae | 16,275 | 0.29 |
| Bacillaceae | 16,002 | 0.29 |
| Bifidobacteriaceae | 13,933 | 0.25 |
| Rhodobacteraceae | 11,919 | 0.21 |
| Flavobacteriaceae | 11,353 | 0.2 |

At the family level, a total of 18 families were predicted. Of these, Prevotellaceae and Bacteroidaceae are the most abundant families containing >82% of the total reads assigned.

2d) Reads assigned to the genus level

| Taxonomy | Number of assigned reads | % of total assignments at genus level |
| --- | --- | --- |
| Prevotella | 3,419,292 | 63.45 |
| Bacteroides | 1,133,762 | 21.04 |
| Faecalibacterium | 168,585 | 3.13 |
| Eubacterium | 168,258 | 3.12 |
| Clostridium | 94,597 | 1.76 |
| Mitsuokella | 72,196 | 1.34 |
| Ruminococcus | 63,963 | 1.19 |
| Alistipes | 42,616 | 0.79 |
| Coprococcus | 39,665 | 0.74 |
| Roseburia | 39,960 | 0.74 |
| Parabacteroides | 27,124 | 0.50 |
| Streptomyces | 26,664 | 0.49 |
| Subdoligranulum | 20,428 | 0.38 |
| Burkholderia | 16,307 | 0.30 |
| Streptococcus | 15,256 | 0.28 |
| Dorea | 14,719 | 0.27 |
| Bacillus | 12,675 | 0.24 |
| Bifidobacterium | 12,476 | 0.23 |

At the genus level, 18 genera were predicted. Of these, Prevotella and Bacteroides are the most abundant and contain >84% of the total reads assigned.

Note: Taxonomic bins with at least 10,000 reads are shown here for assignments at the phylum, family, and genus levels.
