# Supplementary material for: Fast and Accurate Taxonomic Assignments of Metagenomic Sequences Using MetaBin
Source: PLoS One. 2012 Apr 4;7(4):e34030. doi: 10.1371/journal.pone.0034030 (PMC3319535; doi:10.1371/journal.pone.0034030)
Supplement: Table S3 — Comparative analysis of taxonomic assignment of reads by homology- and composition-based methods for the Sargasso dataset (SSea Sample 1). (DOC) [file pone.0034030.s009.doc]

**Table S3** Comparative analysis of taxonomic assignment of reads by homology- and composition-based methods for the Sargasso dataset (SSea Sample 1)

3a) Comparison of reads assigned to various taxonomic levels

| **Taxonomic Rank** | **Homology-based (using Blastx^1^)** | | | **Homology-based (using Blat^2^)** | **Homology-based (using Pfam^3^)** | **Composition-based** | |
| --- | --- | --- | --- | --- | --- | --- | --- |
|  | **MetaBinX** | **MEGAN** | **SOrt-ITEMS** | **MetaBinT** | **WebCARMA** | **TACOA** | **NBC** |
| Phylum | 8,908 | 8,651 | 8,503 | 8,618 | 3,388 | 3,518 | 9,988 |
| Order | 7,659 | 7,356 | 5,917 | 7,695 | 2,694 | 2,544 | 9,744 |
| Class | 7,747 | 7,517 | 6,623 | 7,737 | 2,843 | 2,735 | 8,371 |
| Family | 5,775 | 5,472 | 5,018 | 6,016 | 2,140 | 2,320 | 9,256 |
| Genus | 7,272 | 6,893 | 4,567 | 7,263 | 2,305 | 2,435 | 9,988 |
| Species | 5,026 | 2,054 | 121 | 5,301 | 1,488 | 0 | 9,998 |
| Total Assigned | 9,680 | 9,679 | 8,992 | 9,133 | 3,907 | 8,870 | 9,998 |

^1^ The alignment was carried out using Blastx against complete NR database.

^2^ The alignment was carried out using Blat against complete NR database.

^3^ Homology-based search against protein families in Pfam database.

3b) Comparison of reads assigned at the phylum level

| **Taxonomic Rank** | **Homology-based (Using Blastx^1^)** | | | **Homology-based (Using Blat^2^)** | **Homology-based (Using Pfam^3^)** | **Composition-based** | |
| --- | --- | --- | --- | --- | --- | --- | --- |
| **Phylum** | **MetaBinX** | **MEGAN** | **SOrt-ITEMS** | **MetaBinT** | **WebCARMA** | **TACOA** | **NBC** |
| Proteobacteria | 8,149 | 7,984 | 7,837 | 7,797 | 2,964 | 3,209 | 5,672 |
| Cyanobacteria | 510 | 486 | 491 | 500 | 236 | 53 | 1,448 |
| Bacteroidetes | 104 | 91 | 73 | 163 | 43 | 1 | 108 |
| Actinobacteria | 33 | 19 | 20 | 35 | 28 | 33 | 114 |
| Firmicutes | 17 | 4 | 11 | 31 | 21 | 122 | 1,549 |
| Chlamydiae | 4 | 3 | 0 | 4 | 1 | 17 | 69 |
| Thermotogae | 0 | 0 | 0 | 2 | 1 | 1 | 75 |
| Ascomycota | 0 | 0 | 0 | 0 | 20 | 0 | 438 |
| Glomeromycota | 0 | 0 | 0 | 0 | 0 | 0 | 159 |

3c) Comparison of reads assigned at the family level

| **Taxonomic Rank** | **Homology-based (Using Blastx^1^)** | | | **Homology-based (Using Blat^2^)** | **Homology-based (Using Pfam^3^)** | **Composition-based** | |
| --- | --- | --- | --- | --- | --- | --- | --- |
| **Family** | **MetaBinX** | **MEGAN** | **SOrt-ITEMS** | **MetaBinT** | **WebCARMA** | **TACOA** | **NBC** |
| Burkholderiaceae | 2,953 | 2,901 | 2,678 | 2,944 | 849 | 1,669 | 2,647 |
| Shewanellaceae | 1,778 | 1,742 | 1,720 | 1,771 | 639 | 456 | 1,657 |
| Prochlorococcaceae | 313 | 298 | 304 | 312 | 143 | 4 | 1,031 |
| Aeromonadaceae | 126 | 125 | 124 | 131 | 31 | 0 | 138 |
| Pseudomonadaceae | 110 | 92 | 75 | 115 | 50 | 1 | 127 |
| Enterobacteriaceae | 82 | 59 | 46 | 81 | 67 | 59 | 148 |
| Rhodobacteraceae | 64 | 48 | 21 | 102 | 67 | 0 | 16 |
| Flavobacteriaceae | 18 | 7 | 3 | 78 | 22 | 0 | 93 |
| Vibrionaceae | 14 | 6 | 4 | 31 | 10 | 7 | 114 |
| Rhizobiaceae | 8 | 2 | 0 | 17 | 4 | 1 | 56 |
| Idiomarinaceae | 6 | 6 | 0 | 3 | 3 | 1 | 65 |
| Clostridiaceae | 5 | 2 | 0 | 5 | 1 | 0 | 1,022 |
| Parachlamydiaceae | 3 | 3 | 0 | 3 | 0 | 1 | 68 |
| Bacillaceae | 3 | 0 | 0 | 3 | 9 | 0 | 141 |
| Staphylococcaceae | 1 | 0 | 0 | 0 | 0 | 2 | 76 |
| Legionellaceae | 0 | 0 | 0 | 7 | 1 | 0 | 77 |
| Streptococcaceae | 0 | 0 | 0 | 4 | 1 | 13 | 82 |
| Thermotogaceae | 0 | 0 | 0 | 2 | 1 | 0 | 75 |
| Lactobacillaceae | 0 | 0 | 0 | 1 | 0 | 2 | 51 |
| Schizosaccharomycetaceae | 0 | 0 | 0 | 0 | 0 | 0 | 149 |
| Natranaerobiaceae | 0 | 0 | 0 | 0 | 0 | 0 | 103 |

3d) Comparison of reads assigned at the genus level

| **Taxonomic Rank** | **Homology-based (Using Blastx^1^)** | | | **Homology-based (Using Blat^2^)** | **Homology-based (Using Pfam^3^)** | **Composition-based** | |
| --- | --- | --- | --- | --- | --- | --- | --- |
| **Genus** | **MetaBinX** | **MEGAN** | **SOrt-ITEMS** | **MetaBinT** | **WebCARMA** | **TACOA** | **NBC** |
| Burkholderia | 2,831 | 2,742 | 2,214 | 2,841 | 812 | 1,669 | 2,546 |
| Shewanella | 1,778 | 1,742 | 1,584 | 1,771 | 638 | 456 | 1,657 |
| Candidatus Pelagibacter | 1,494 | 1,489 | 184 | 1,229 | 255 | 0 | 82 |
| Prochlorococcus | 313 | 298 | 267 | 312 | 143 | 4 | 1,031 |
| Synechococcus | 166 | 148 | 111 | 162 | 76 | 0 | 153 |
| Aeromonas | 126 | 125 | 118 | 128 | 30 | 0 | 111 |
| Pseudomonas | 100 | 80 | 49 | 98 | 45 | 1 | 122 |
| Cupriavidus | 16 | 26 | 0 | 14 | 4 | 0 | 60 |
| Vibrio | 10 | 4 | 0 | 26 | 7 | 7 | 95 |
| Idiomarina | 6 | 6 | 0 | 3 | 3 | 1 | 65 |
| Clostridium | 5 | 2 | 0 | 5 | 1 | 0 | 913 |
| Flavobacterium | 4 | 3 | 0 | 3 | 2 | 0 | 76 |
| Candidatus Protochlamydia | 3 | 3 | 0 | 0 | 0 | 0 | 68 |
| Psychroflexus | 2 | 2 | 0 | 58 | 5 | 0 | 0 |
| Trichodesmium | 1 | 0 | 0 | 2 | 1 | 0 | 214 |
| Yersinia | 1 | 0 | 0 | 1 | 2 | 56 | 49 |
| Oceanobacillus | 1 | 0 | 0 | 0 | 4 | 0 | 54 |
| Legionella | 0 | 0 | 0 | 7 | 1 | 0 | 77 |
| Lactobacillus | 0 | 0 | 0 | 1 | 0 | 2 | 51 |
| Candidatus Koribacter | 0 | 0 | 0 | 0 | 1 | 0 | 154 |
| Staphylococcus | 0 | 0 | 0 | 0 | 0 | 2 | 75 |
| Glomus | 0 | 0 | 0 | 0 | 0 | 0 | 159 |
| Schizosaccharomyces | 0 | 0 | 0 | 0 | 0 | 0 | 149 |
| Alkaliphilus | 0 | 0 | 0 | 0 | 0 | 0 | 109 |
| Natranaerobius | 0 | 0 | 0 | 0 | 0 | 0 | 103 |
| Debaryomyces | 0 | 0 | 0 | 0 | 0 | 0 | 62 |

Note: Taxonomic bins with at least 50 reads are shown here for comparisons at the phylum, family, and genus levels.
