# Supplementary material for: Fast and Accurate Taxonomic Assignments of Metagenomic Sequences Using MetaBin
Source: PLoS One. 2012 Apr 4;7(4):e34030. doi: 10.1371/journal.pone.0034030 (PMC3319535; doi:10.1371/journal.pone.0034030)
Supplement: Table S4 — Comparative analysis of human gut datasets HGF1T and HGF1S. (DOC) [file pone.0034030.s010.doc]

**Table S4** Comparative analysis of human gut datasets HGF1T and HGF1S

**4a)** Comparative analysis of reads assigned to the phylum level

| Phylum | HGF1T | HGF1S | Proportion* |
| --- | --- | --- | --- |
| Firmicutes | 2,195 | 1,384 | 1.59 |
| Bacteroidetes | 639 | 2,447 | 0.26 |
| Proteobacteria | 241 | 123 | 1.96 |
| Actinobacteria | 238 | 79 | 3.01 |
| Fusobacteria | 12 | 6 | 2 |
| Verrucomicrobia | 10 | 9 | 1.11 |

**4b)** Comparative analysis of reads assigned to the family level

| Family | HGF1T | HGF1S | Proportion* |
| --- | --- | --- | --- |
| Bacteroidaceae | 462 | 2,132 | 0.22 |
| Ruminococcaceae | 377 | 642 | 0.59 |
| Clostridiaceae | 351 | 190 | 1.85 |
| Veillonellaceae | 271 | 31 | 8.74 |
| Bifidobacteriaceae | 170 | 63 | 2.7 |
| Lachnospiraceae | 137 | 51 | 2.69 |
| Enterobacteriaceae | 118 | 13 | 9.08 |
| Desulfovibrionaceae | 63 | 48 | 1.31 |
| Erysipelotrichaceae | 56 | 14 | 4 |
| Rikenellaceae | 51 | 67 | 0.76 |
| Eubacteriaceae | 51 | 12 | 4.25 |
| Coriobacteriaceae | 48 | 6 | 8 |
| Porphyromonadaceae | 40 | 93 | 0.43 |
| Streptococcaceae | 21 | 9 | 2.33 |
| Fusobacteriaceae | 12 | 6 | 2 |
| Paenibacillaceae | 11 | 1 | 11 |

**4c)** Comparative analysis of reads assigned to the genus level

| Genus | HGF1T | HGF1S | Proportion* |
| --- | --- | --- | --- |
| Bacteroides | 462 | 2,132 | 0.22 |
| Clostridium | 334 | 181 | 1.85 |
| Faecalibacterium | 278 | 543 | 0.51 |
| Mitsuokella | 240 | 10 | 24 |
| Bifidobacterium | 170 | 63 | 2.7 |
| Ruminococcus | 61 | 81 | 0.75 |
| Roseburia | 61 | 24 | 2.54 |
| Desulfovibrio | 58 | 35 | 1.66 |
| Alistipes | 51 | 67 | 0.76 |
| Dorea | 45 | 17 | 2.65 |
| Eubacterium | 44 | 11 | 4 |
| Collinsella | 40 | 3 | 13.33 |
| Parabacteroides | 39 | 91 | 0.43 |
| Anaerotruncus | 33 | 11 | 3 |
| Escherichia | 32 | 3 | 10.67 |
| Streptococcus | 21 | 8 | 2.63 |
| Thermosinus | 14 | 14 | 1 |
| Blautia | 14 | 4 | 3.5 |
| Coprococcus | 13 | 5 | 2.6 |
| Fusobacterium | 12 | 6 | 2 |
| Anaerostipes | 12 | 2 | 6 |
| Paenibacillus | 11 | 1 | 11 |

*Proportion is calculated as ‘Number of reads assigned to HGF1T/Number of reads assigned to HGF1S’.

Note: Taxonomic bins with at least 10 reads are shown here for comparisons at the phylum, family, and genus levels.
