# Supplementary material for: Fast and Accurate Taxonomic Assignments of Metagenomic Sequences Using MetaBin
Source: PLoS One. 2012 Apr 4;7(4):e34030. doi: 10.1371/journal.pone.0034030 (PMC3319535; doi:10.1371/journal.pone.0034030)
Supplement: Text S1 — Description of possible cases (A–G as shown in Figure 1). (DOC) [file pone.0034030.s011.doc]

**Description of possible cases (A-G as shown in Figure 1)**

The detailed explanations of possibilities (A-G) as shown in Figure 1 are provided below. The long reads and short reads in these cases refer to read length ≥ 400 bp and < 400 bp, respectively.

**A**: reads derived entirely from intergenic regions. Figure S3 shows that many reads in all simulated read datasets are derived from the intergenic region, even if the average intergenic regions are small (~182 bp on average) in the selected microbial genomes (Figure S4). However, as expected, the probability of a read originating entirely from the intergenic region decreases with the increasing length of the read, and also with shorter intergenic distance. Since the NR reference database contains only protein sequences, correct hits for intergenic reads are not expected; however, some small peptide-like regions in such reads may still get ambiguously aligned against NR with alignment (Blastx or Blat) bit-scores above the used cut-off. Also, there is a possibility that the intergenic region from one genome may code for a true ORF from another genome (Example A).

**B**: reads that contain only short coding regions at either of the terminals (Example B and C). This includes partial ORFs containing protein start or end regions and random peptide sequences. In these cases, the bit-score is expected to be low, even less than 35, which is generally used as the cut-off in other methods [1,2], leaving these reads to be classified as unassigned by other methods. To indentify such cases, we have relaxed our bit-score cut-off to 29 in case of Blastx and 17 in case of Blat (Example D). However, reducing the bit-score cut-off to 29 may increase the number of false and random matches. Since our rationale for reducing this cut-off is only to include the partial terminal ORFs; therefore, to avoid any false positive matches due to random peptides, we confirm the presence of the 5’ or 3’ region of the protein match (hit) in such hits.

**C**: long reads (Sanger) that contain a complete ORF (long) in the middle and two small partial ORFs at the terminals. In these cases, Blastx or Blat will preferably identify the longer ORF as the best hit since it shows the longest match with the reference protein. The other two terminal partial ORFs will usually have lower bit-scores, and therefore may not be identified when selecting a bit-score cut-off range of up to 90% of the best hit. To identify such cases, we first find all the unique coding regions in the query sequence that showed a protein match in NR, and then classify them as Main (M), which is the main hit region, N-terminal (N), and C-terminal (C) (Example E). We consider only the ORFs from these regions because in most cases a longest possible read (Sanger method, length 800-1000 bp) is expected to contain at most one complete and up to two partial ORFs at the ends. The N and C terminal regions are then checked using the same strategy as in Case B. This additional information from the terminal ends adds confidence to the assessment and reduces the need for LCA analysis (Figure 1 and 2).

**D**: reads that contain complete ORFs. These are obviously one of the easiest regions to identify and make taxonomic assignments since there is only one best hit matching region expected in these cases.

**E**: reads that contain long partial ORF at either of the terminal ends of the query. In these cases, since the match is of considerable length, the bit-score is likely to be above the cut-off and therefore can be easily assigned a taxonomic class as in case D.

**F**: reads that derive completely from genic regions. These cases are more likely for short reads and are also easier cases to assign a taxonomic classification as in cases D and E.

**G**: read that contain sequencing errors that cause the ORF to be split into two parts. These cases are similar to case C and will be treated similarly.

**Example A) An example of a case when a short simulated illumina read is derived from the intergenic region of ECOLI (Case A of Figure 1).**

>r894_4350022-4350095_74|gi|170079663|ref|NC_010473.1| ECOLI_Escherichia coli str. K-12 substr. DH10B, complete genome

TTCCTGGCATCCTGGACGGTGATGCCCCTACGGTTGCCCTCGCCAGCACGGGCATCGGTAAAGCGTAAGGTTCA

**Blastx results**
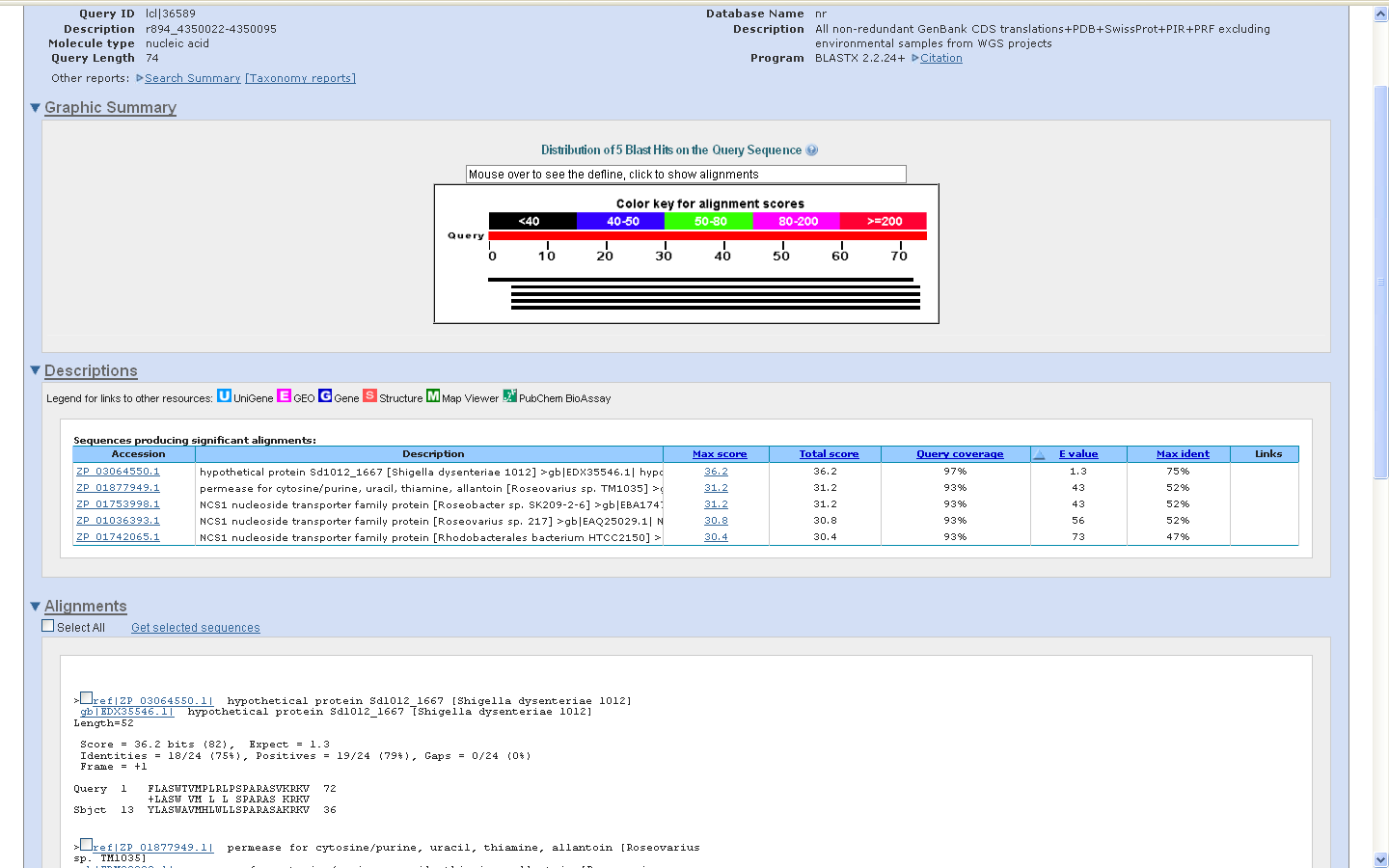


Only the alignment for the selected hit for taxonomic assignment is shown. In this case, the difference between the bit-scores of the first and the second hits is more than 10% of the first (best) hit bit-score, so only the first hit is considered for assignment. The match almost entirely covers the query. By using MetaBin and MEGAN the above read was assigned to *Shigella dysenteriae*.

**Example B) Case of a read containing a short partial ORF at either of the terminals (Case B of Figure 1).**

>ARBU_154240_154590|351

AAATCTAAATGCCATCAATTAACTCCATAATAGATTTTTAATAAGATTAGCTAAATATTTATGAAAATAAGAGTAAATTAGAATTATTGTAAATTTAAAAAAATTTTTAACAATACTTAGAAATTTTAAGATAGTAAGTTTCTTAGTGAAAAAATTATAATTAGATTGATTGAAATTATAAAATGGCACGCCTGGTAGGAGTCGAACCCACAACCCACCGGGTCGAAACCGGTTATTCTATCCAGTTGAACTACAGACGCACCTAAAATTAGGATGTAAGTCTATCCTAATATTTCTTAAATTTTAAAAAAAGATATTATCTAAAACTATTAAAAGGAAGAAAACTATTCC


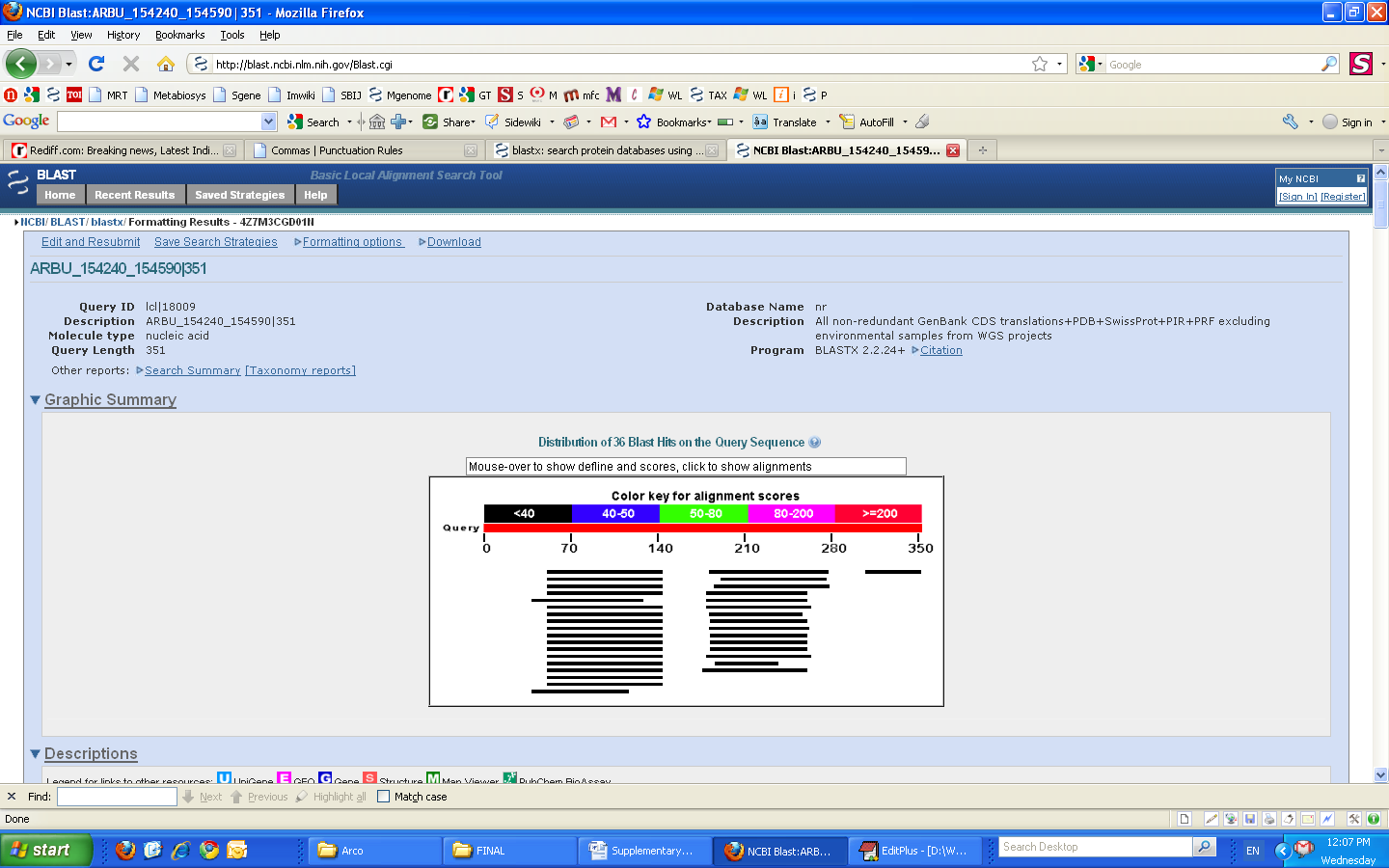


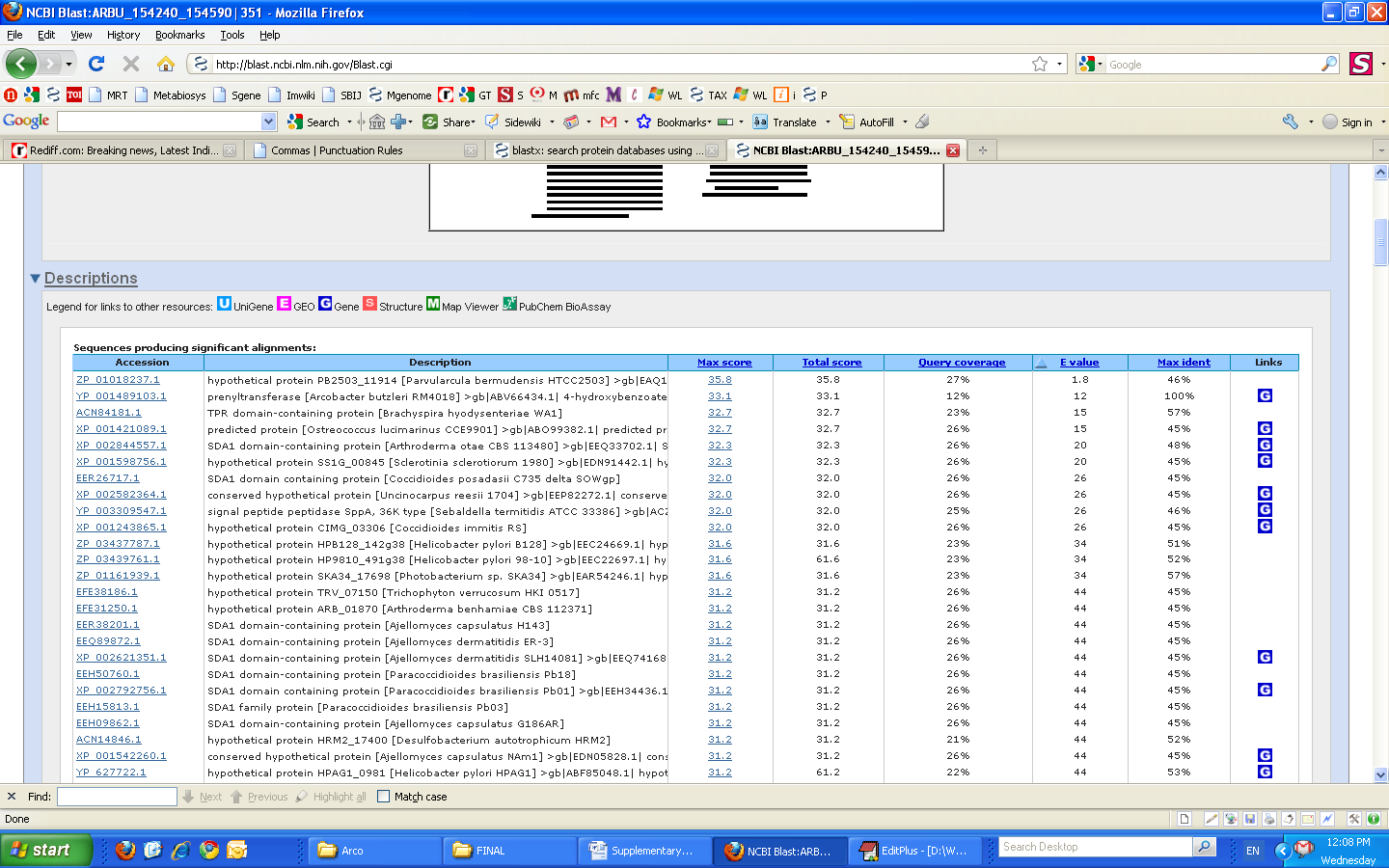


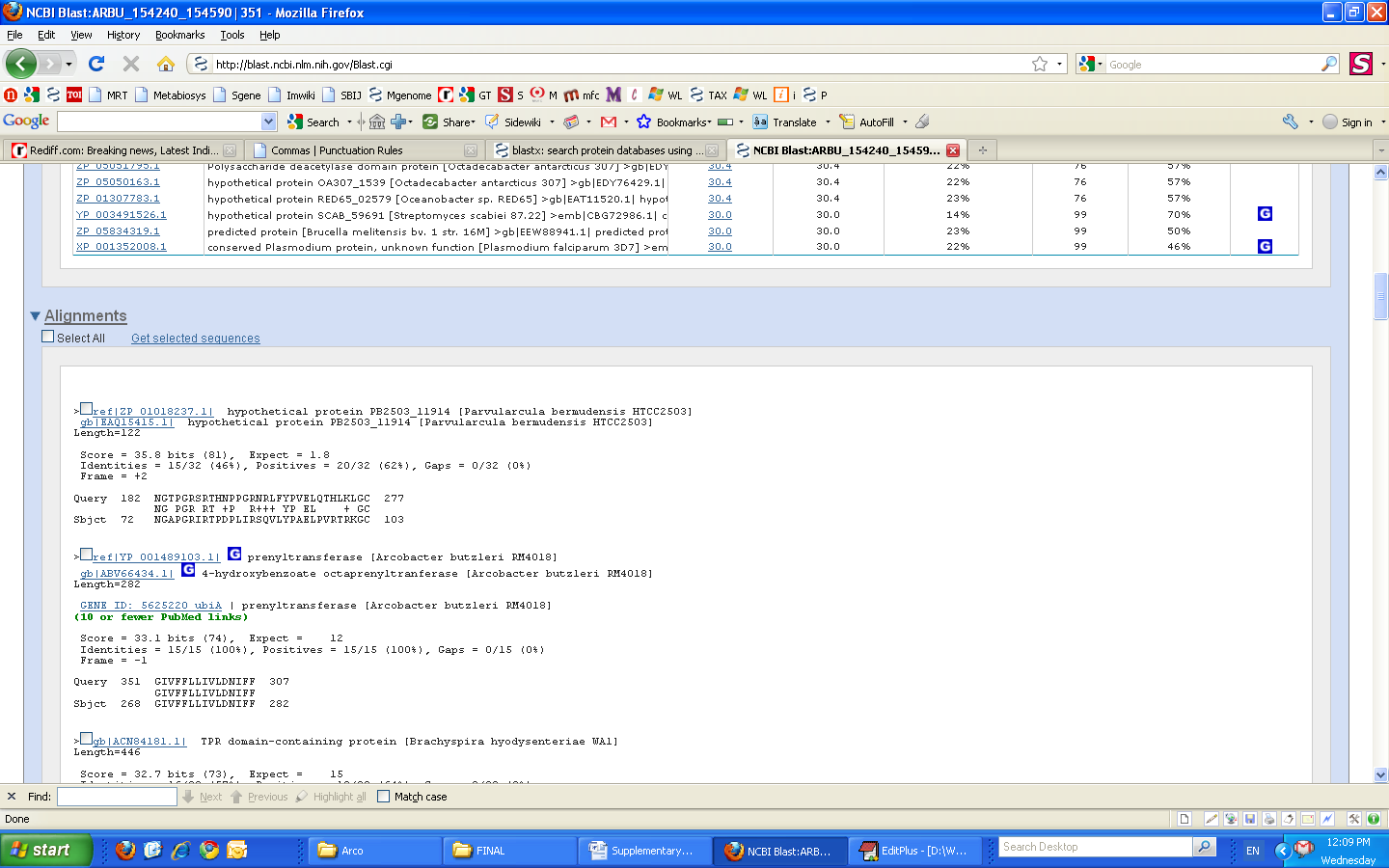


Alignments for top two hits are shown. In case of the above example, the first hit is found for the middle (182-277) region of the query with bit-score 35.8. As evident from the above alignment, this appears to be a random match. The second hit is from the C-terminal of the query showing a match with the 3’-end region of the matched protein (hit) ‘>gi|157736420|ref|YP_001489103.1| prenyltransferase [Arcobacter butzleri RM4018]’. The complete end (268-282) of the hit (complete protein length is 282 amino acids) is present with a match identity of 100%. Therefore, our algorithm considers this hit as the first best hit for taxonomic assignment even if its bit-score is less (33.1) than the bit-score of the previous hits (previous best bit-score 35.8).

The other software like SOrt-ITEMS will not consider this hit because its bit-score is less than 35. Instead they would consider only the first three hits because only the hits with bit-score of 0.9 of best bit-score are selected. In case, MEGAN is used by lowering the bit-score to 29, it will consider the first ten hits (bit score range 35.8-32) for LCA which would again lead to an incorrect assignment as ‘cellular organisms’ due to the erroneous consideration of random matches (hits).

**Example C):** **Blastx results for a read containing a short partial ORF at the terminal (Case B of Figure 1).**

>ARBU_120590_120795|206

TTTTACCCACAAATAAACCAATTCCATAAAAAACTCTTTATAAATAATTTTATTACTAATCTTAGTTAAAAAAGACTTATTTTATACAAAGCTAGACTACTTTACTTTTCTTTGGATATAATCTTTGAAAAATTTTATAAAATATATAAATTCAAAAGGAAATAAAAATGCCATTATTAGATAGTTTTAGAGTTGACCATACGATT

**
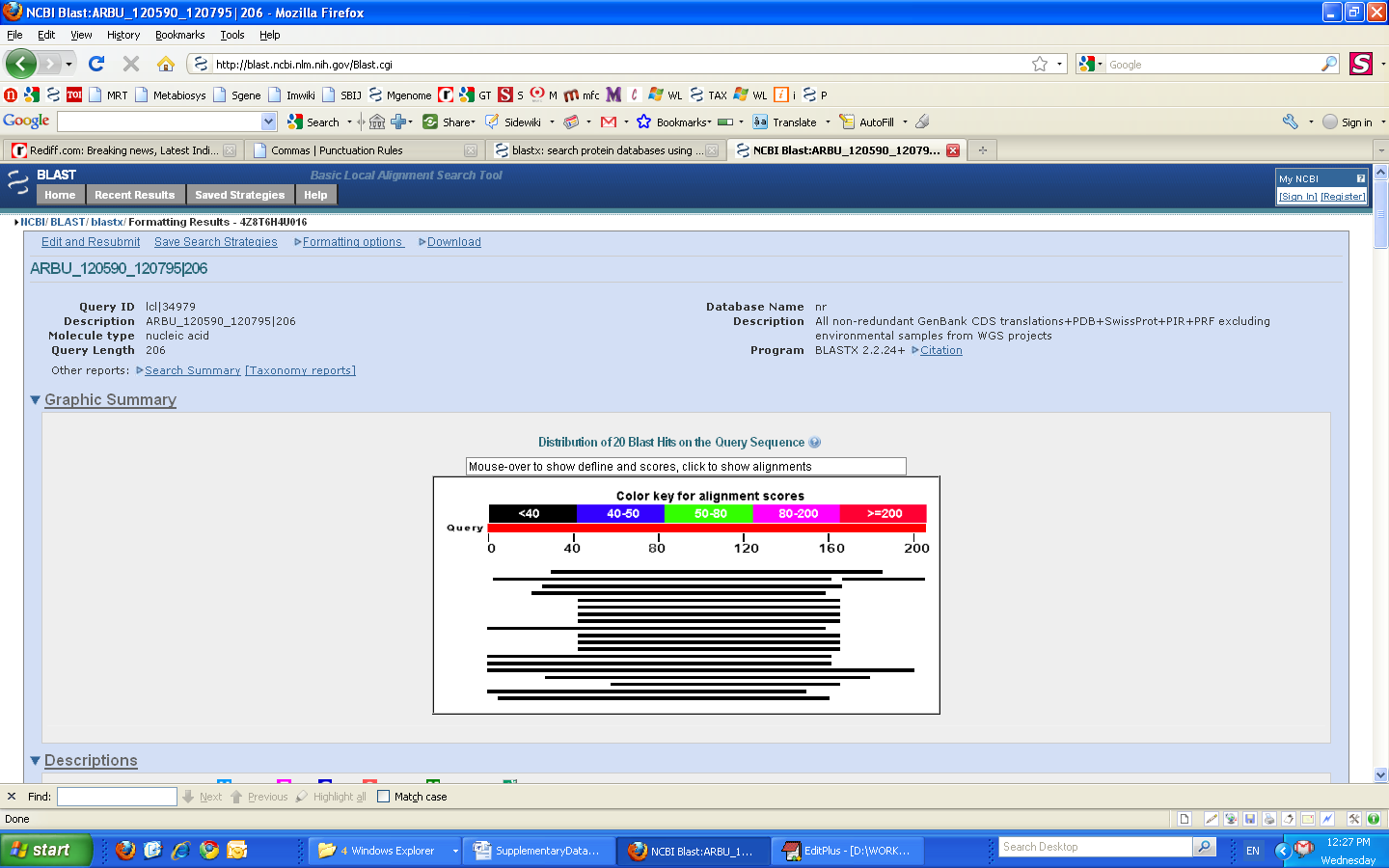
**

**
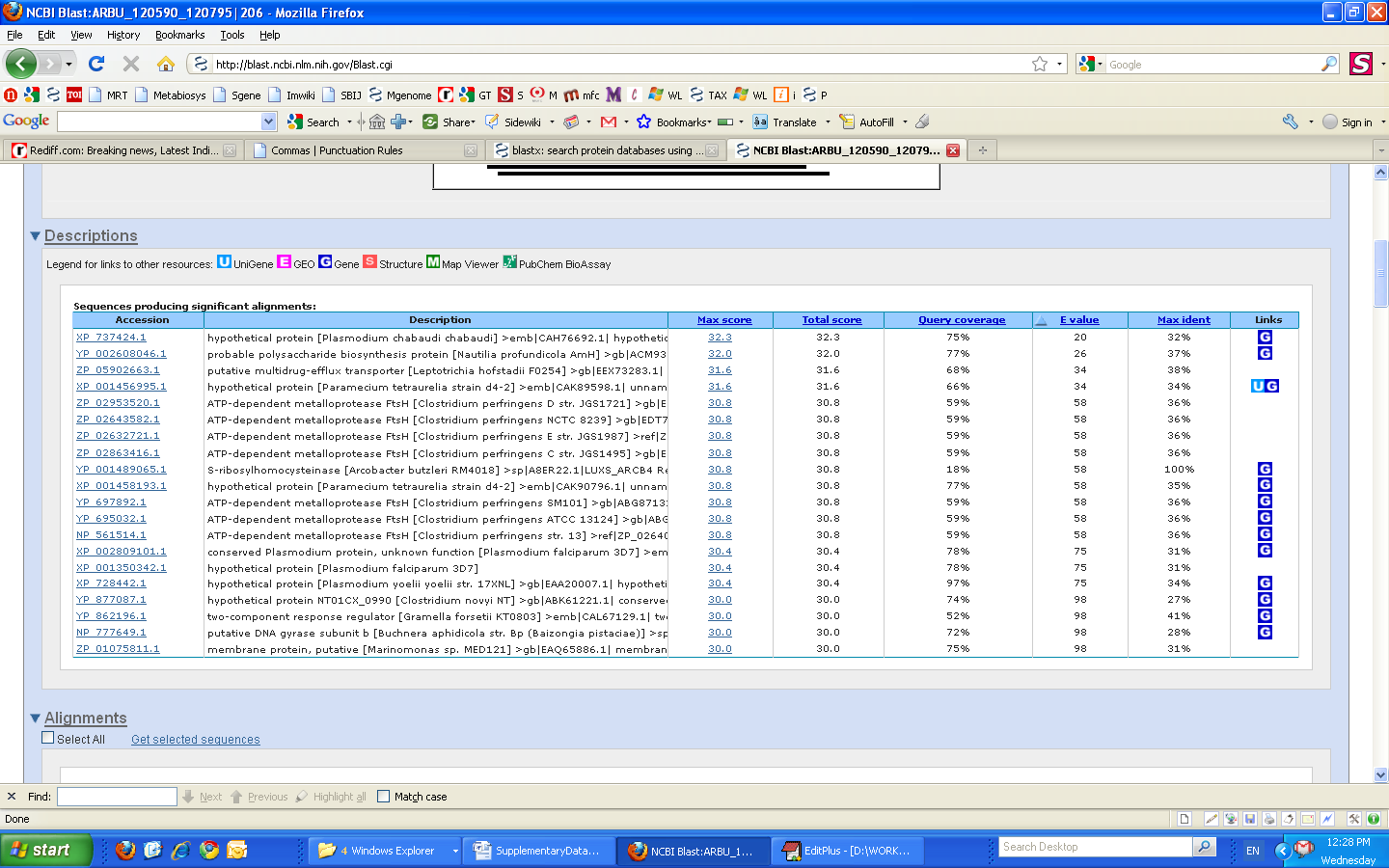
**

**
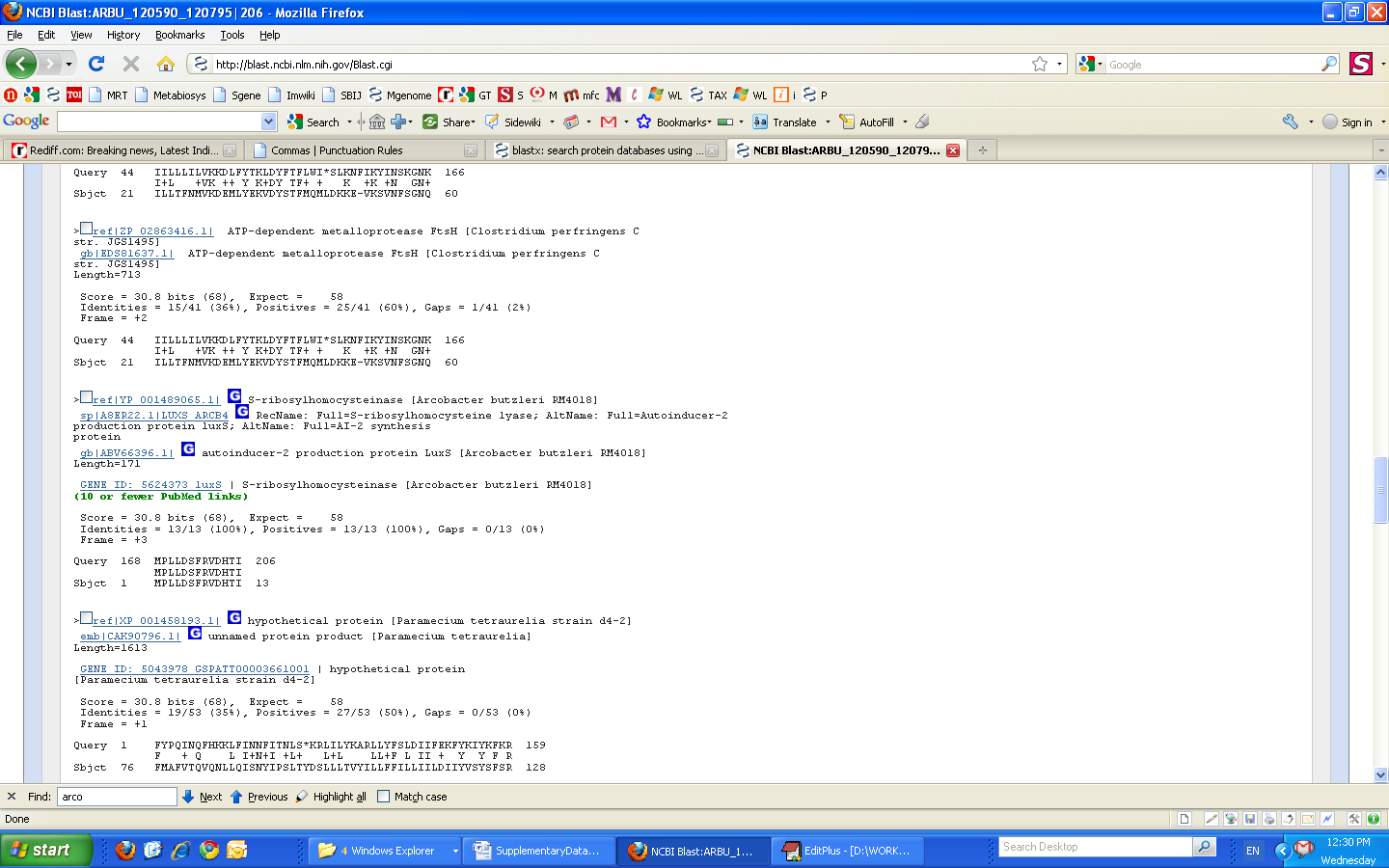
**

Only the alignment for the hit selected for taxonomic assignment is shown. In case of the above example, the correct match is found at the C-terminal of the query. The complete start (N terminal) of the hit is present (1-13 of 171 amino acids) and is matched with an identity of 100%. Therefore, our algorithm will consider only this hit for assignment even if its Blastx bit-score is less (30.8) than the bit-score of the previous hits (best bit-score is 32.3).

The other software like SOrt-ITEMS will not consider this hit because its bit-score is less than 35. In case, MEGAN is used by lowering the bit-score to 29, it will consider the first hits (bit score range 32.3-29 using 0.9 of best score) for LCA which would again lead to an incorrect assignment as ‘cellular organisms’ due to the erroneous consideration of random matches (hits).

**Example D) Example of a short illumina read showing a match at a very low bit-score**

>NC_009850.fna_981929_981970|42

GGCAAATGTAAAAGGTAATTTTAGTGTTCAAGTTGGAGCATT

**Blastx alignment against NR**

**
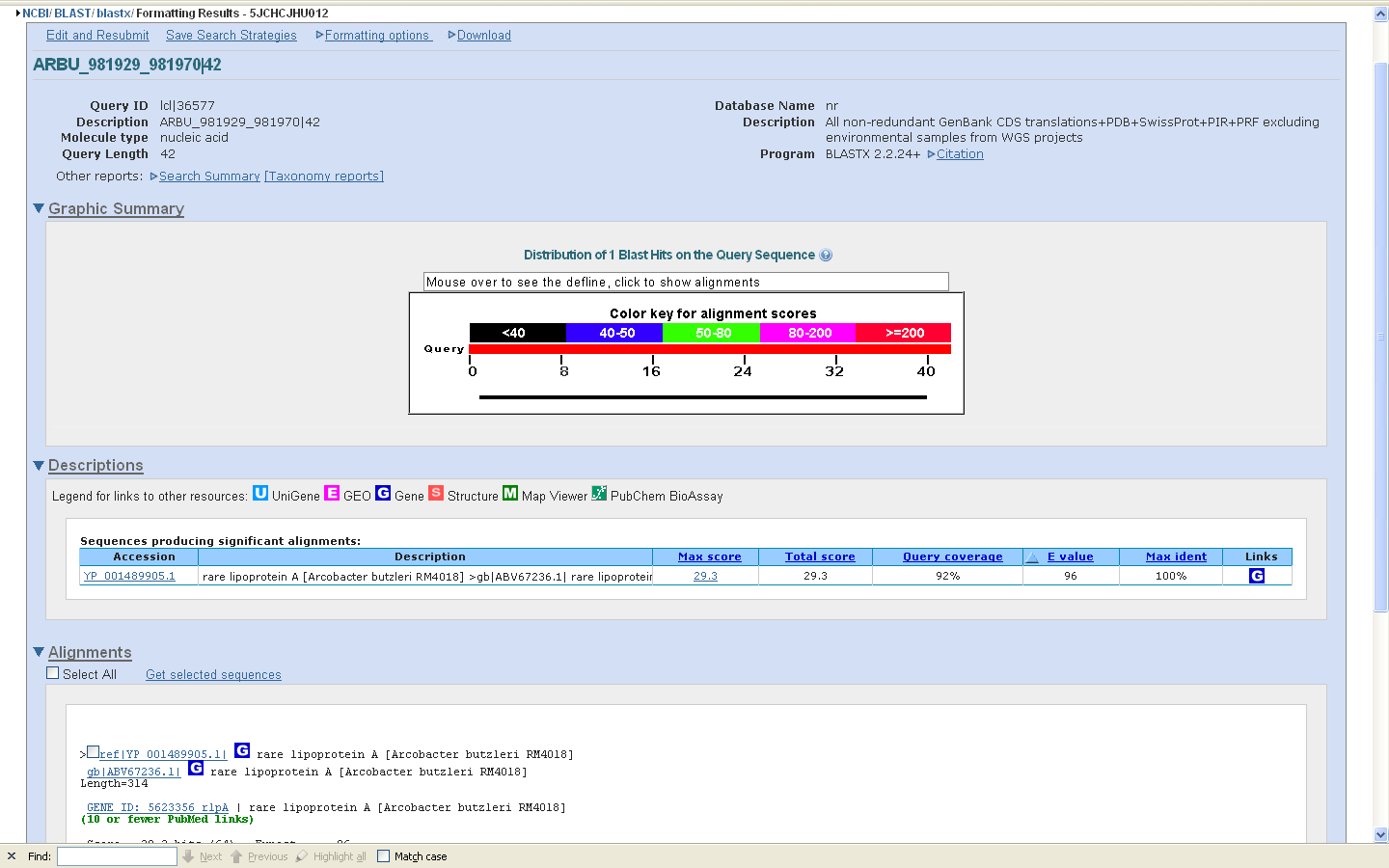

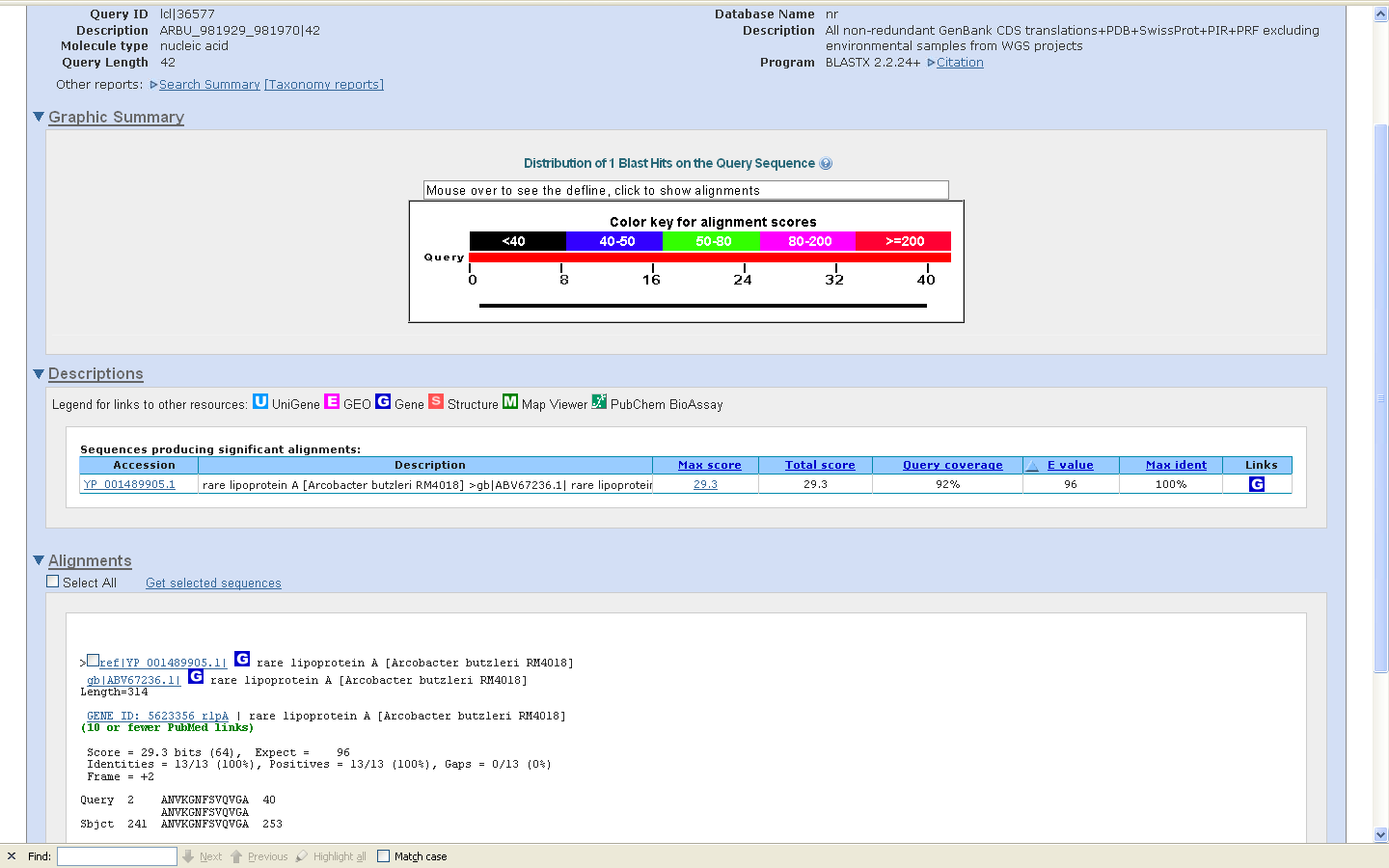

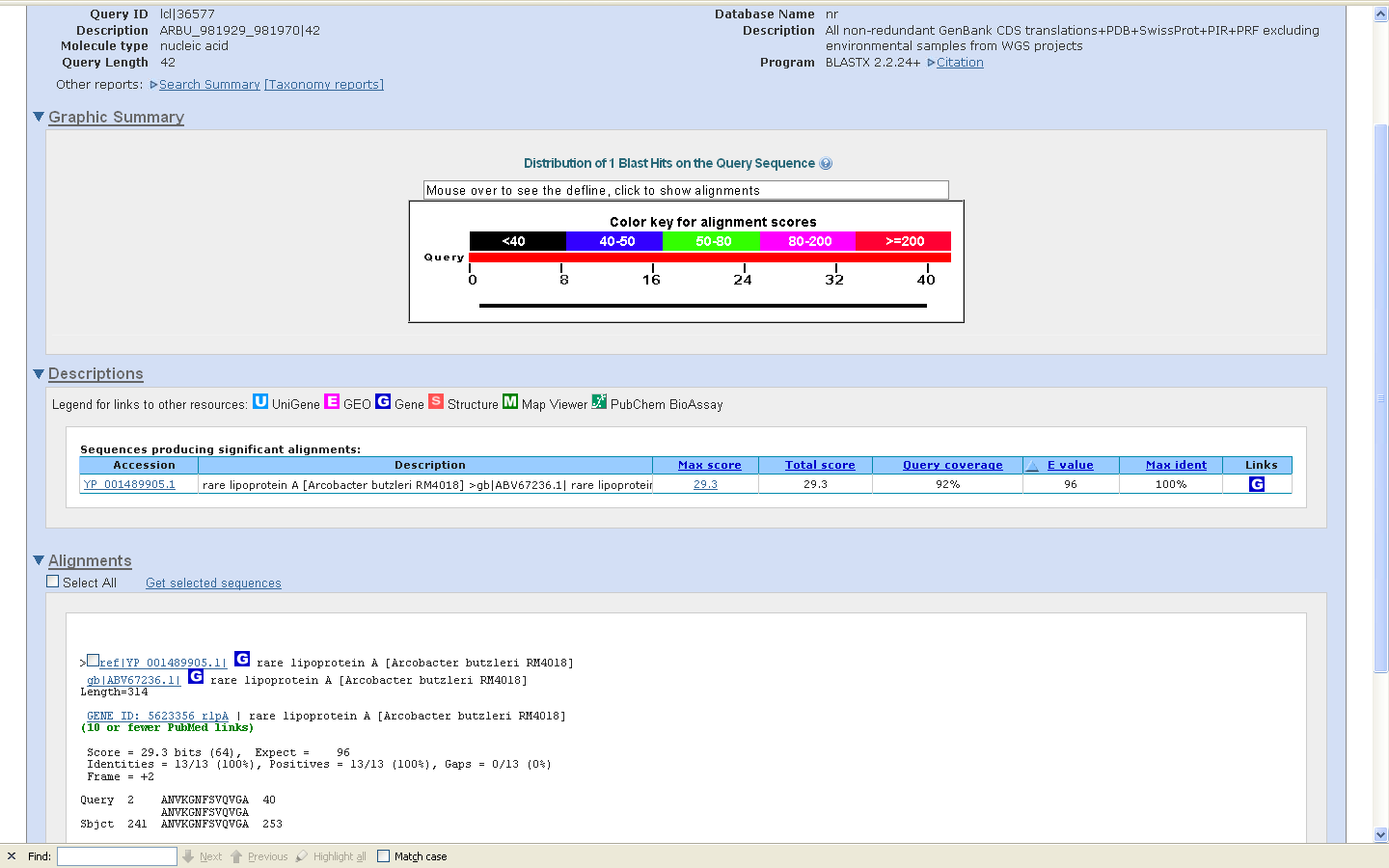
**

**
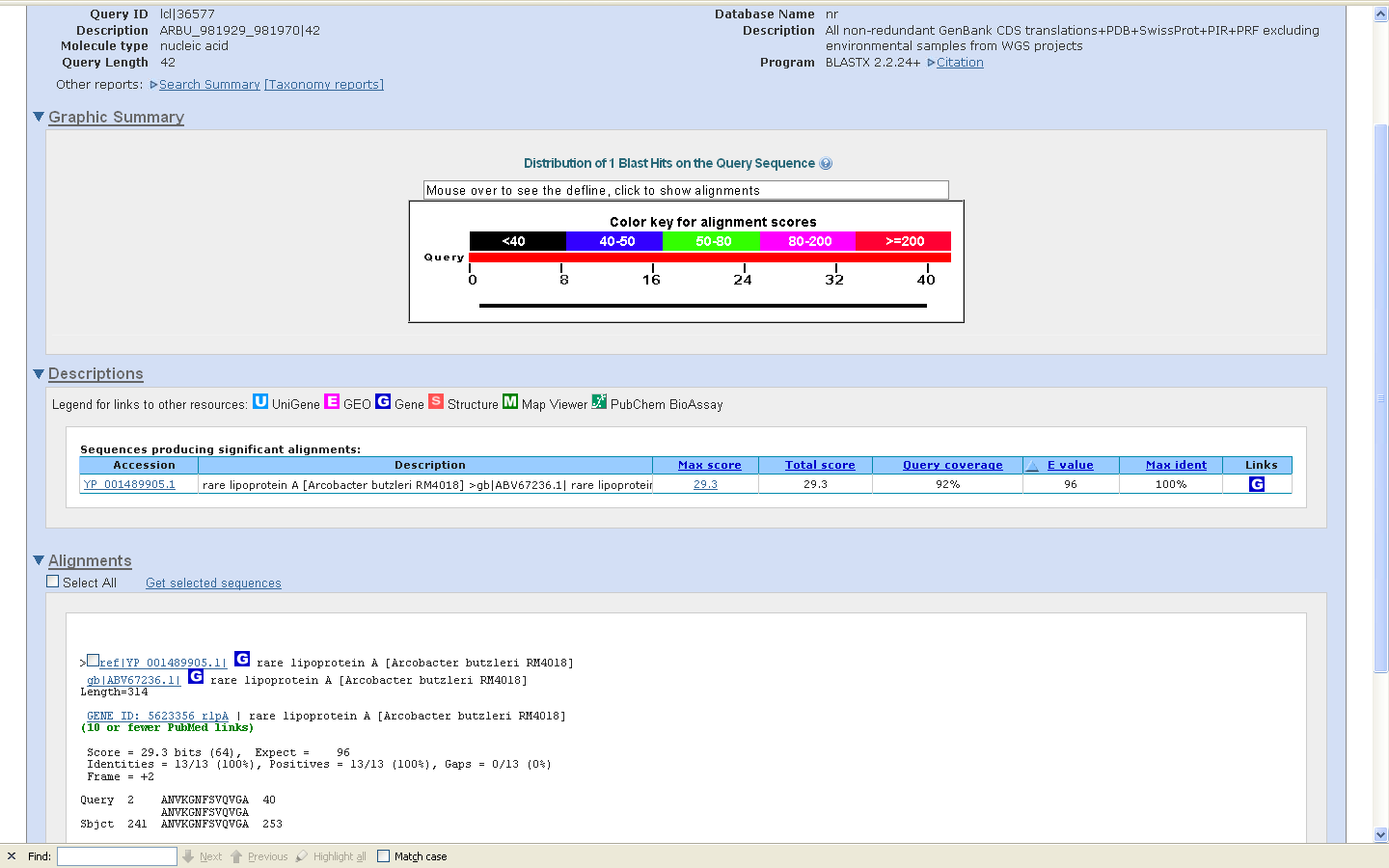
**

In the above example, the illumina read of length 42 bp showed a perfect match for the region 2-42, with 100% identity, but with a low bit-score. Such cases helped us to select the minimum bit-score cut-off of 29.

**Example E) Case of a read with two or more partial or complete ORFs**

>NC_009850.fna_54421_54913|493

GTTAAAAAAATAATTGCTGCTTGGGTTATTACCGTTCCTGCTTCTGCTCTTTTGGCTGCAGGTATCTTTTATATGATTAAAGGTATCGTTATTGTATAAGTAGAATTTTCTACTTATACAATCTATTTCTTAACTTCTAATTTACATTAGGAACTTATTATCGAAGCTATATTCTCTGTTTTACCTATTTATTTTTTCATTTTTTTAGGTTTTATTGCAAAAAAAAGATTTACAACACAAATTGATGAAAAAACTTTAGTTTTATTATCTTTATATTTTTTCCAACCAATTTTGATTCTTTGGGGATTGACAAAATCACCTATAAACTATGAATTTATAATGTCACCACTTTTTTATATTATCATTGTTTTTACAACTTTATCTTTTTTAATATTTTTTAGTAAGATAATTTTTGATTCGCGTACAGATGAGTCAATATATTTAGGAACTGCTCTTATTGGAAATACAGGAAATCTTGGAATTCCTCTTGGAA

**Blastx alignment against NR**

**
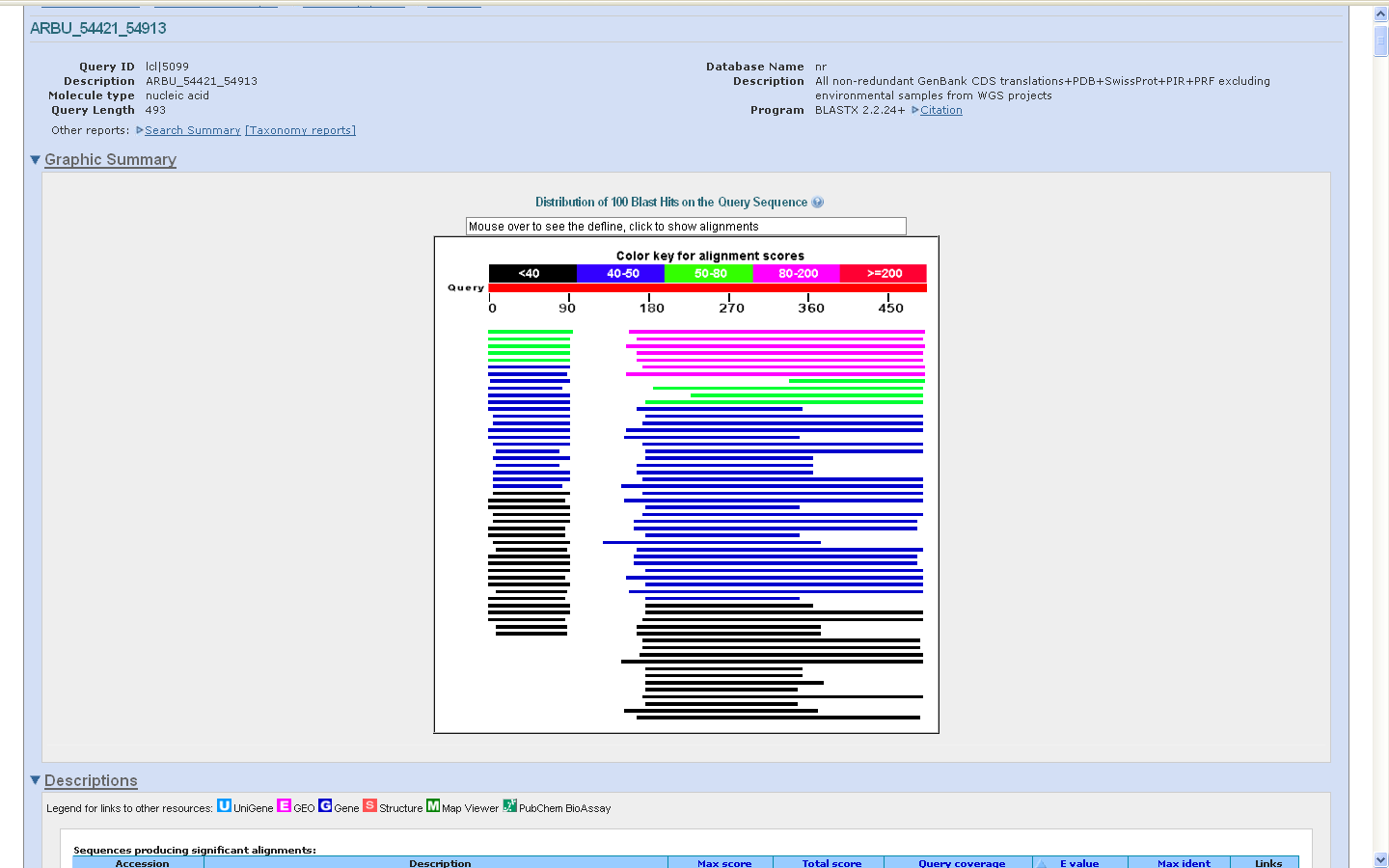
**


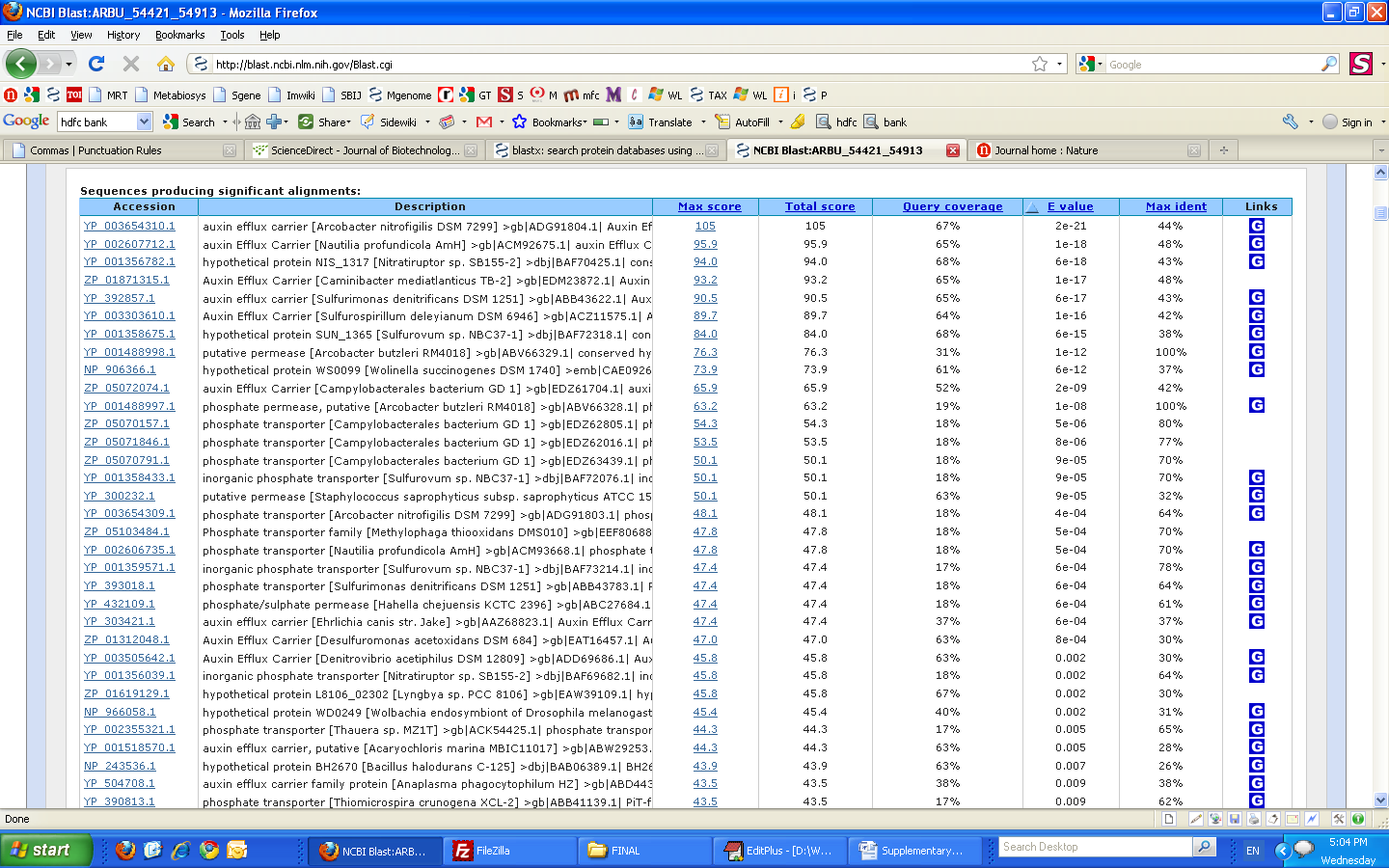


**Alignment from the C-terminal region (160-492) of the read (read length=493 bp)**

**
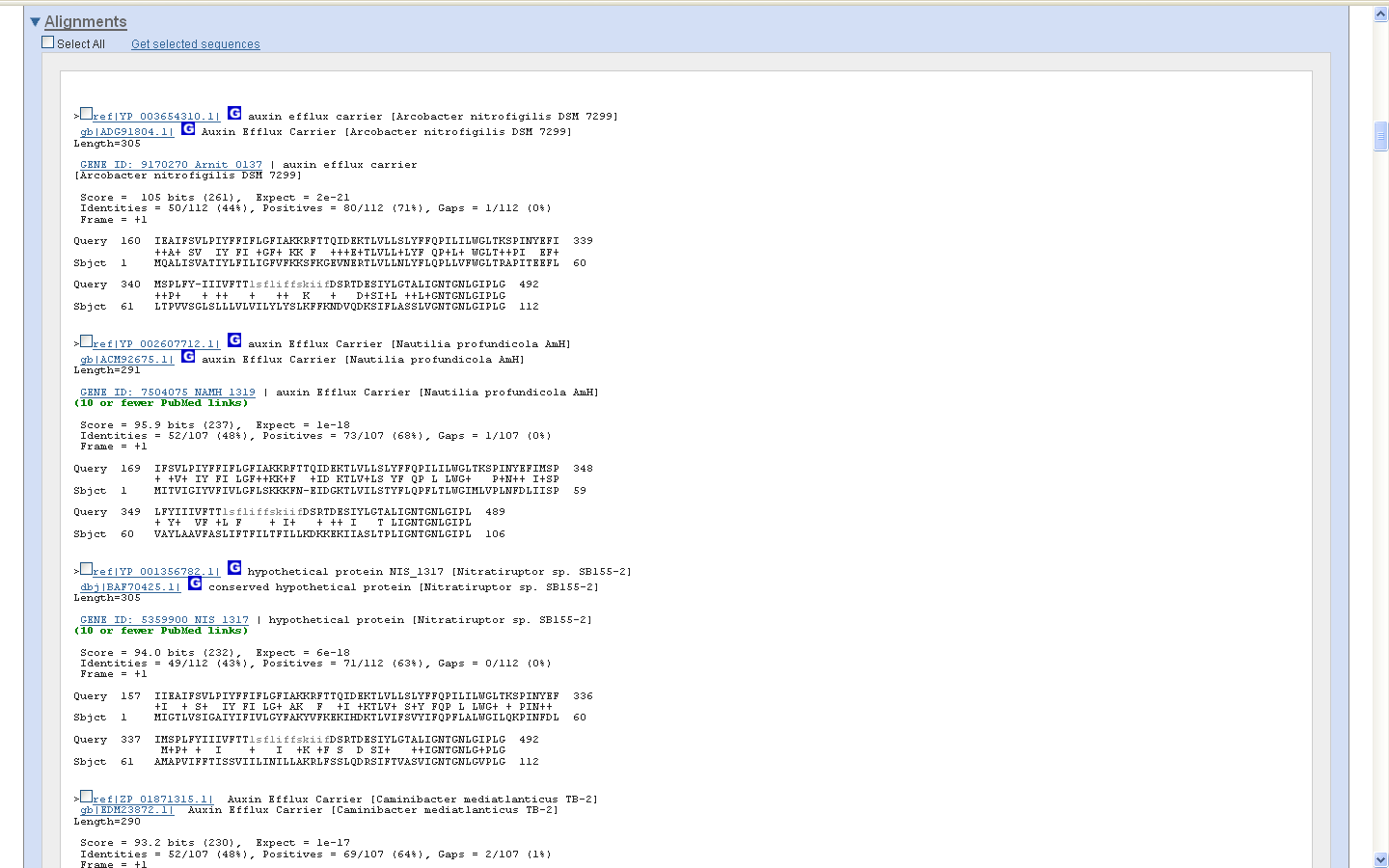
**

**Alignment from the N-terminal region (1-93) of the read (read length=493 bp)**

**
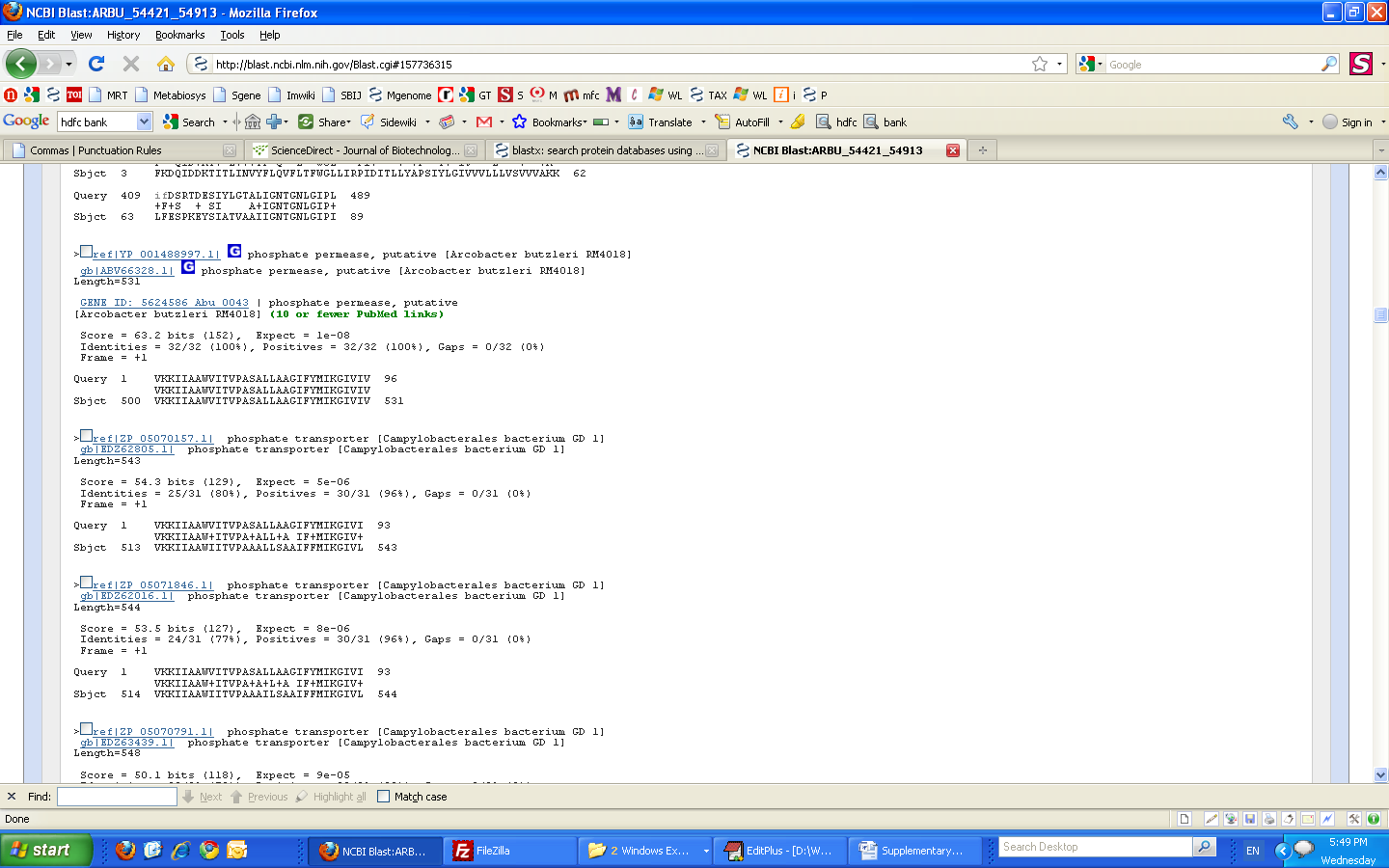
**

Selected alignments of hits for the selected regions (ORFs) are shown. In the above case, the top bit-score is 105, so MEGAN and SOrt-ITEMS would only select hits with bit-score lying in the range 95-105, considering the criteria of taking all hits with bit-score 0.9 of the best bit-score. This would lead to the consideration of two genomes namely *Arcobacter nitrofigilis DSM 7299*, and *Nautilia profundicola* *AmH*, which would lead to a higher taxonomic level assignment (based on LCA), even if this read is from Arcobacter.

Our algorithm, on the other hand, first finds all unique coding regions in the query. Two unique ORF regions (1-93 and 162-492) were found at the N and C terminals of the query sequence. Now, after parsing the Blastx or Blat output, MetaBin would consider both these ORFs for taxonomic assignment even if the C-terminal region was the best hit (bit-score=105). Therefore, from the C-terminal ORF, both *Arcobacter nitrofigilis DSM 7299*, and *Nautilia profundicola AmH* would be selected, and from the N-terminal ORF only *Arcobacter butzleri RM4018* would be selected. Now, the algorithm will find a common genome (taxonomic ID) from both these regions and would assign Arcobacter as the taxonomic bin of the read.

Reference List

1. Huson DH, Auch AF, Qi J, Schuster SC (2007) MEGAN analysis of metagenomic data. Genome Res 17: 377-386.

2. Monzoorul HM, Ghosh TS, Komanduri D, Mande SS (2009) SOrt-ITEMS: Sequence orthology based approach for improved taxonomic estimation of metagenomic sequences. Bioinformatics 25: 1722-1730.
