# Supplementary material for: Fast and Accurate Taxonomic Assignments of Metagenomic Sequences Using MetaBin
Source: PLoS One. 2012 Apr 4;7(4):e34030. doi: 10.1371/journal.pone.0034030 (PMC3319535; doi:10.1371/journal.pone.0034030)
Supplement: Text S2 — Supplementary information. (DOC) [file pone.0034030.s012.doc]

**Text S2**

*Description of FigureS1*

In order to carry out the taxonomic reassignment of one member of a paired-end read to another, in the case when one of them has a more reliable assignment, we assigned a weight to each read of the pair. This weight has two components based on, A) %Identity (ID) and %Coverage (COV) of the read with the protein hit present in the NCBI NR database, and B) common taxonomic ID(s) (TID) for the multiple ORFs contained in a read. The final weight is calculated by adding the partial weights of the above two components. A read can get a maximum weight of 16 (potential best match) as per the following methodology.

For calculating the contribution (partial weight) of the first component (%ID and %COV), i) for reads that show a %ID ≥90 and %Positives ≥95 along with a %COV of ≥60, the maximum partial weight is 15, ii) for the remaining reads that show a %ID of ≥90 and a %COV of ≥60, the maximum partial weight is 9 since it is calculated as ID/10, and iii) for the remaining reads, the maximum partial weight is 4 since it is calculated as ID/20 where ID is <90. The partial weight values are rounded down to the nearest whole number

For calculating the contribution (partial weight) from the second component, the following procedure is used. For a read, multiple (three) ORFs (N-terminal, C-terminal and M, Figure1) are considered. Now the partial weight is assigned as, i) if a common taxonomic ID(s) is assigned for all three ORFs, a partial weight of 6 is allocated, ii) if two out of the three ORFs are assigned the same taxonomic ID(s), a partial weight of 4 is allocated, and (iii) if there is no common taxonomic ID assigned to the three ORFs, a partial weight of 1 is allocated.

As shown in Figure S1, the total weight is calculated by adding the partial weights of components A and B. Based on the final weight, there can be three possible cases as described below.

Case I (Best case): In the case when a read shows a % ID ≥90 and %Positives ≥95 along with a %COV of ≥60, common taxonomic IDs are not calculated since a match with such high identity and coverage should be the best possible match for the read. So, the total weight assigned will be 16 (15+1; 15 (from component A) + 1 (from component B, since the partial weight from multiple ORFs is not considered in this case).

Case II: When %ID is ≥90 and %COV is ≥60%, the maximum total weight can be 15 as shown below.

9+6=15 (The maximum partial weight contribution from component A is 9 (%ID ≥90, ID/10 is rounded off to 9), and the maximum partial weight contribution from component B is 6 (all three ORFs shared at least one common taxonomic ID).

Other possible weights for case II can be 13 (9+4 (two ORFs with same TID)) or 10 (9+1 (no common TID)).

Case III: When %ID <90, the maximum total weight can be 10 as shown below.

4+6=10, (The maximum partial weight contribution from component A is 4 (%ID <90, so if it is 89 then it is calculated as 89/20=4.45 which is rounded off as 4), and the maximum partial weight contribution from component B is 6 (if all three ORFs shared at least one common taxonomic ID).

In the above weight calculation, as apparent, more importance is given to the percent identity and coverage of a read with the hit, since if a read shows %ID ≥90, %Positives ≥95 and %COV≥60, it should be a good enough match to decide the taxonomic origin of the read. Similarly, greater importance is given to a read that contains multiple ORFs with all sharing the same TID, indicating the likelihood of that read originating from a genome of that TID.

Criteria for reassignment:

- If the same taxonomic ID or lineage is assigned to both reads of a pair, do not reassign the taxonomic bin.
- Else, if different taxonomic lineages are assigned to the reads of a pair, and if the difference in the weights is > 2 and the higher weight is ≥ 10; reassign the taxonomic bin to the lower weight read from that of the higher weight read.
- Else, if only one read is assigned with a taxonomic bin and the other read remains as ‘Not assigned’ or had ‘No hits’ from Blat or Blastx, assign the taxonomic bin of the assigned read to the unassigned read.

In the above criteria, a weight ≥10 indicates that the read either has at least %ID ≥90 and %COV≥60, or it has having multiple ORFs sharing the same taxonomic group (TID). Also, a difference of 2 units in weights (along with a total weight ≥10) indicates that one of the reads has found a better match to a taxonomic group, so the other one can be satisfactorily assigned to that one.

*Description of results obtained from simulated reads analysis*

To validate MetaBin on simulated metagenomic data, we carried out taxonomic analysis of the simulated read datasets using MetaBin with both Blastx and Blat output (referred to as MetaBinX and MetaBinT, respectively), and with MEGAN and SOrt-ITEMS using the Blastx output on various simulated read datasets. For all the bacterial read datasets, in most comparisons including longer reads (800 bp, 450 bp and 250 bp) and all comparisons including shorter reads (75 bp and 45 bp), MetaBinX assigned a higher percentage of reads to their correct genus, family and phylum, as compared to MEGAN and SOrt-ITEMS (Table 1 and 2, details in Text S2 and S3). As apparent from Table 1 and 2, the performance of SOrt-ITEMS was much weaker in all comparisons to MetaBinX and in most comparisons to MEGAN. For longer read lengths, the performance of MetaBinT was mostly comparable to that of MetaBinX, MEGAN and SOrt-ITEMS for NR, but for NRminusGenus and NRminusFamily it was slightly weaker. MetaBinX and MetaBinT both performed significantly better for short reads and assigned, up to 18% for NR, and up to 7% for NRminusGenus and up to 9% for NRminusFamily, more reads to the correct genus and phylum, respectively as compared to MEGAN. It is apparent that SOrt-ITEMS could assign a lower percentage of reads at the deeper taxonomic levels such as genus and family however, the assignments at the phylum level were comparative in many cases. The ability of MetaBin to make more accurate assignments at the lower and more specific taxonomic levels underscores its usefulness.

The average sensitivity and PPV of MetaBinX for all simulated read datasets was also similar or higher as compared to MEGAN and SOrt-ITEMS, especially for short reads. MetaBinT showed comparable sensitivity and PPV for NR, but its PPV was lower for NRminusGenus, though the sensitivity was considerably higher. This could be attributed to the differences in the accuracy and sensitivity of the Blastx and Blat algorithms. In the case of simulated reads derived from the bacterial genomes, for ~75 bp reads, both MetaBinT and MetaBinX showed 2.8-6.6% higher average sensitivity for NR, NRminusGenus and NRminusFamily as compared to MEGAN, and showed 14.5-16.8% higher average sensitivity for NR, NRminusGenus and NRminusFamily as compared to SOrt-ITEMS. In the case of ~45 bp reads, for NR both MetaBinT and MetaBinX showed >6% higher average sensitivity, for NRminusGenus, MetaBinT showed 10.4% and MetaBinX showed ~7% higher sensitivity, and for NRminusFamily, MetaBinT showed ~32% and MetaBinX showed 17% higher sensitivity as compared to MEGAN. For the same read length (~45 bp), both MetaBinT and MetaBinX showed 31-46% higher average sensitivity, for NR, NRminusGenus, and NRminusFamily, as compared to SOrt-ITEMS.

*Interesting observations from simulated read analysis using MetaBin*

At the genomic level, we made some interesting observations. For only ECOLI, the total assignments made at the genus level using Blastx against NR, by both MetaBin and MEGAN, were significantly lower (<14%) as compared to the other genomes (>77%) (Text S3). However, at the family and phylum levels, there was not much difference in the total or correct assignments for ECOLI, as compared to other genomes. On the contrary, in the case of Blastx against NRminusGenus, the percentage of reads assigned to the correct family and phylum, by both MetaBin and MEGAN, were much higher for ECOLI as compared to all other genomes. These observations appear crucial for homology-based taxonomic binning approaches. A plausible explanation for the first case could be that the members of enterobacteriaceae family are highly similar at the genomic level. As a result, some reads had significant and equivalent (bit-score) matches from more than one genome of the same family. Therefore, the programs could not resolve the taxonomic assignment to a single genome and thus carried out LCA resulting in the assignment of reads at the family level rather than at the genus or species level. An explanation for the second case could be that the enterobacteriaceae family contains the most genomes from several genera as compared to the other families. So, even if a genus of this family is deleted, a read is likely to match to other genomes belonging to other genera of this family, increasing the probability of assigning a correct taxonomic lineage at the family level or, at least at the phylum level, as observed here.

In the case of BOAF, Blastx against NRminusGenus made very few assignments at the phylum level. This can be explained for the same reason as in the second case above. The [Spirochaetaceae](http://www.ncbi.nlm.nih.gov/Taxonomy/Browser/wwwtax.cgi?mode=Info&id=137&lvl=3&p=mapview&p=has_linkout&p=blast_url&p=genome_blast&lin=f&keep=1&srchmode=1&unlock) family contains only 31 genomes from two genera, Borrelia and Treponema, with 27 and four genomes, respectively. If the Borrelia genus is deleted, the probability of finding a match from the remaining four genomes of the Treponema genus is severely limited. Similarly, in case of THYE, Blastx against NRminusGenus made very few assignments at the phylum level because only a few completely sequenced genomes are available in its phylum.

The above observations indicate that the likelihood of correct taxonomic assignment will increase with the availability of more closely related genomes (same family). However, these assignments will be limited to higher levels, instead of at the species or genus levels, due to the similarity among the genomes resulting in multiple hits, and will require more strict parameters for assignments in future algorithms.

In the cases of RSD17 and CFP2, which are unculturable endosymbiotic bacteria, for Blastx against NR, the number of reads assigned to the genus and phylum levels are comparatively lower than for the other genomes. In the case of Blastx against NRminusGenus with the CFP2 genus deleted, the numbers of reads assigned at the phylum level were comparable to the other genomes. This indicates that even for taxonomic assignments of sequence reads derived from unculturable bacterial genomes or novel genomes, homology-based approaches still perform well as long as some other genomes from the same phylum are known.

*Validation on the Sargasso sea dataset*

We compared the results of MetaBinX (using Blastx), MEGAN, SOrt-ITEMS, WebCARMA, MetaBinT (using Blat), TACOA and NBC by using the same sample data ‘SSea sample 1’ from the Sargasso sea dataset which was used earlier in other studies [1,2]. The first three programs (homology-based search using Blastx) were used with similar parameters: a bit-score cutoff of 35, a 10% bit-score range of the best bit-score, and a minimum taxonomic bin size of at least two reads. About 97% of the reads could be assigned to different taxonomic groups by both MetaBinX and MEGAN, while SOrt-ITEMS assigned about 90% of the reads (Table S3a). The total assignments by MetaBinT were less than both MetaBinX and MEGAN, but assignments at specific levels such as species, genus, family, class or order were higher than for all other methods. Considering only the bins with at least 50 reads at the phylum level (Table S3b), most phyla were commonly predicted by MetaBin (using Blastx and Blat), MEGAN, and SOrt-ITEMS. At the family and genus levels (Table S3c and S3d), the predicted taxonomic bins by MetaBin (using Blastx and Blat) and MEGAN were similar, of which a few were also common to SOrt-ITEMS.

It is clear from these results that MetaBin and MEGAN both predict similar bins at the phylum, family and genus levels, indicating the correctness of the results. Furthermore, MetaBinX and MetaBinT assigned comparatively more reads to each of these common bins, up to the phylum and order levels, which shows their unique ability to identify more reads (sensitive) and assign them to the correct bins (accurate); attesting to its higher accuracy and sensitivity for real metagenomic data. It is also noteworthy that MetaBin (both variants) assigned more than twice the number of reads at the species level as compared to MEGAN, which indicates that the performance of MetaBin is more specific. Overall, the performance of SOrt-ITEMS was comparatively poor compared to both MetaBin and MEGAN, and was limited to higher taxonomic levels. Though the focus of this study is on homology-based approaches, just to provide a brief comparison of MetaBin with two composition-based methods (TACOA and NBC), as well as with another method based on homology to protein families (WebCARMA), we ran these programs on the same dataset (as analyzed above). As apparent from the results, the TACOA (composition-based) and WebCARMA (protein family based) methods have limitations for making taxonomic assignments; and both perform poorly compared to homology-based methods. However, another composition-based method, NBC, showed unusually high number of total assignments as it assigned almost all the reads to the phylum or even to the genus level, which is surprising given these sample reads were derived from a metagenomic environment (Sargasso sea) where a large number of the genomes are novel (yet cultured and not yet sequenced). Therefore, an almost absolute taxonomic assignment at the genus or even at the phylum level is certainly not expected with the current knowledge. As mentioned above, the homology-based methods predicted similar bins at the phylum, family and genus levels which also corroborate with the results (phylum level) from the previous study on the entire Sargasso sea dataset [1]. However, NBC also assigned a significant number of reads to additional phylums.

*Comparative analysis using human gut datasets*

To demonstrate the comparative analysis feature of MetaBin, we used the human gut data and analyzed the Blastx results for both HGF1S and HGF1T datasets using MetaBin with a minimum bin size of two reads (Table S4). In the dendrogram shown in Figure S5, the HGF1S dataset is represented in red and HGF1T in blue. When a taxonomic bin is commonly present in both datasets, its respective normalized proportions are shown as a pie chart with the above assigned colors. Since HGF1S and HGF1T are from individuals belonging to the same family, it is apparent (considering only the genera with at least 10 reads) that their guts contain similar flora, but have surprisingly different amounts of the constituent microbes. All phyla were common to both datasets, but ‘Bacteroidetes’ was the most abundant phylum for HGF1S, whereas ‘Firmicutes’ was the most abundant phylum for HGF1T. At the family and genus levels, all taxonomic bins were common to both datasets, but their relative numbers showed much variation. Even though the datasets analyzed are only subsets of the original data, the results corroborate with the results of the previous study on the entire human gut dataset [3], indicating the correctness and usefulness of MetaBin.

The above human gut data was generated as paired-end reads; therefore, we could analyze this data using the ‘-p’ option of MetaBin for taxonomic reassignment considering the reads as paired-ends. There were 2,406 and 2,463 paired-end reads for HGF1S and HGF1T, respectively. For a total of 67 and 89 read pairs, in HGF1S and HGF1T, respectively, both reads remained either unassigned or had no Blat hits. For the remaining read pairs, the pre-calculated weight (Figure S1) of the reads was used to reassign taxonomic bins to the read pairs which were previously assigned to different taxonomic lineages. For some read pairs, only one of the reads could be assigned to a taxonomic bin, while the other read remained unassigned, or had no Blastx hit as might be expected for reads derived from the intergenic regions (Figure 1). For such read pairs, the taxonomic bin of the assigned read was directly allocated as the taxonomic bin of the unassigned read. For HGF1S, 1,915 read pairs were assigned to the same lineage and, out of 277 read pairs which were assigned to different lineages, 69 pairs could be reassigned to the same lineage; however, the remaining 208 read pairs could not be reassigned since there was no apparent taxonomic similarity between the reads. For HGF1T, 1,807 read pairs were assigned to the same lineage. Out of 338 read pairs which were assigned to different lineages, 82 pairs could be reassigned to the same lineage, and the remaining 256 read pairs could not be reassigned since there was no apparent taxonomic similarity between the reads. For the 214 and 318 read pairs of HGF1S and HGF1T, respectively, where one of the reads was assigned as ‘Not assigned’ or ‘No hits’, the taxonomic bin of the assigned read was directly allocated as the taxonomic bin of the unassigned read. Thus, our approach is also useful for taxonomic assignment of read pairs where one of the members remains taxonomically unassigned or had no Blastx hits.

***Time taken on a real metagenomic dataset containing more than 20 million reads***

The performance of MetaBin was validated on real metagenomic data obtained by Illumina sequencing from a Spanish male individual (V1CD2) (details are provided in the Methods section). The ‘prepareinput’ program which prepares the input file for Blat alignment by translating the nucleotide reads into all six reading frames (RFs) was used. The output file containing these RFs was then divided into 22 parts, and each part was aligned against NR using Blat on 22 processors, each with 16 GB of RAM, and the outputs were concatenated into a single file (149 GB). MetaBin (the ‘metabin’ program) was then run on a single processor (with 32 GB of RAM) to carry out the taxonomic assignments. Only those bins containing at least 10,000 reads were considered, while the rest of the parameters used the default values. In total, it took about 370 CPU hours, which is significantly reasonable considering the input size of more than 20.72 million reads. We estimated that for the same number of reads, the Blastx program would have taken almost two years on a single processor using similar system configuration as used above in case of Blat.

*Comparison of time taken to analyze the Blastx output by MetaBin and MEGAN*

**Although MetaBin comprehensively examines the Blastx output for all possible coding regions (ORFs), which is an additional task as compared to MEGAN, it is comparatively faster than MEGAN (Figure S6). This is an additional advantage of using MetaBin for the analysis of large datasets. In addition, after running MetaBin once on a file, the next run (using the same or different input parameters) will be much faster, as MetaBin saves the formatted file after parsing the Blastx results, thus eliminating this step in subsequent runs, which consumes most of the total time taken.**

*Methods* *(additional information on Test sequences and database construction)*

**To test the performance of MetaBin, local versions of the NR database were created for 23 of the 27 selected genomes by removing all sequences belonging to the associated genus and family. These are referred to as NRminusGenus and NRminusFamily, respectively, in the manuscript text. Since the genus information is not known for** RSD17**, and CAPH, DITH, and GEAU were the only sequenced genomes in their genus,** these were not deleted from the NR database to create the NRminusGenus databases. For these genomes, the taxonomic assignments were calculated at one higher taxonomic level.

*Reasoning for using a reference database that includes information from both prokaryotes and eukaryotes*

We included models from both prokaryotic and eukaryotic genomes while using the web-based version of WebCARMA and also in the case of the NBC classifier. Similarly, the NR database which was used as a reference for the homology-based taxonomic assignments by MetaBin and MEGAN contained proteins from both prokaryotes and eukaryotes. So, for the evaluation of both, homology- and composition-based methods (except TACOA with which only prokaryotic models were available), the reference databases/models were not limited to only prokaryotes but included information from both prokaryotes and eukaryotes. It is important that the taxonomic assignments should not be restricted by using only prokaryotic genomes/proteins since the objective of these taxonomic assignment methods is to identify the taxonomic-based origin of these reads. Therefore, in cases where the microbial sample is extracted from organismal environments like human gut, these methods can also serve as a tool to check for and estimate the presence of contaminating eukaryotic sequences in the metagenomic datasets.

*Availability*

MetaBin web-server and standalone program is freely available at http://metabin.riken.jp/. This is the main web server which should be used to carry out large-scale analysis.

An alternate web server to download the standalone program is available at http://metasystems.riken.jp/metabin/.

Reference List

1. Huson DH, Auch AF, Qi J, Schuster SC (2007) MEGAN analysis of metagenomic data. Genome Res 17: 377-386.

2. Monzoorul HM, Ghosh TS, Komanduri D, Mande SS (2009) SOrt-ITEMS: Sequence orthology based approach for improved taxonomic estimation of metagenomic sequences. Bioinformatics 25: 1722-1730.

3. Kurokawa K, Itoh T, Kuwahara T, Oshima K, Toh H, Toyoda A, Takami H, Morita H, Sharma VK, Srivastava TP, Taylor TD, Noguchi H, Mori H, Ogura Y, Ehrlich DS, Itoh K, Takagi T, Sakaki Y, Hayashi T, Hattori M (2007) Comparative metagenomics revealed commonly enriched gene sets in human gut microbiomes. DNA Res 14: 169-181.
