# Supplementary material for: Fast and Accurate Taxonomic Assignments of Metagenomic Sequences Using MetaBin
Source: PLoS One. 2012 Apr 4;7(4):e34030. doi: 10.1371/journal.pone.0034030 (PMC3319535; doi:10.1371/journal.pone.0034030)
Supplement: Text S3 — Genome wise summary of analysis by MetaBinX and MetaBinT on 25 simulated read datasets. (DOC) [file pone.0034030.s013.doc]

**Text S3**

**Genome wise summary of analysis by MetaBinX and MetaBinT on 25** simulated read datasets.

| **MetaBinX against complete NR for read length ~800 bp** | | | | | | | | | |
| --- | --- | --- | --- | --- | --- | --- | --- | --- | --- |
| **Genome** | **TGENUS** | **CGENUS** | **TFAMILY** | **CFAMILY** | **TPHYLUM** | **CPHYLUM** | **UNASS** | **NOHITS** | **INTERGENIC** |
| ACFE | 968 | 968 | 968 | 968 | 971 | 971 | 2 | 0 | 23 |
| AQAE | 930 | 925 | 975 | 975 | 982 | 982 | 2 | 0 | 9 |
| ARBU | 967 | 967 | 970 | 970 | 984 | 984 | 0 | 0 | 11 |
| BOAF | 979 | 979 | 979 | 979 | 979 | 979 | 0 | 0 | 19 |
| BSUB | 926 | 926 | 940 | 940 | 958 | 958 | 1 | 0 | 36 |
| CAPH | 884 | 883 | 885 | 884 | 885 | 884 | 5 | 0 | 105 |
| CBOT | 920 | 917 | 924 | 922 | 935 | 935 | 0 | 0 | 58 |
| CFP2 | 829 | 827 | 841 | 841 | 843 | 843 | 5 | 0 | 146 |
| CHAB | 897 | 897 | 955 | 955 | 958 | 958 | 1 | 0 | 38 |
| DITH | 964 | 964 | 964 | 964 | 964 | 964 | 0 | 0 | 26 |
| ECOLI | 170 | 137 | 892 | 891 | 930 | 929 | 0 | 0 | 54 |
| FLJO | 902 | 901 | 908 | 908 | 959 | 958 | 1 | 0 | 36 |
| FUNU | 958 | 957 | 960 | 959 | 960 | 959 | 0 | 0 | 22 |
| GEAU | 981 | 980 | 981 | 980 | 981 | 980 | 0 | 0 | 10 |
| LACB | 936 | 935 | 943 | 942 | 952 | 952 | 2 | 0 | 43 |
| MTUB | 949 | 949 | 974 | 974 | 975 | 975 | 0 | 0 | 20 |
| PRAC | 949 | 949 | 949 | 949 | 962 | 962 | 1 | 0 | 35 |
| RHSP | 895 | 895 | 927 | 927 | 970 | 970 | 0 | 0 | 29 |
| RSD17 | 782 | 779 | 782 | 779 | 783 | 779 | 4 | 0 | 202 |
| STMU | 932 | 932 | 941 | 941 | 967 | 967 | 0 | 0 | 25 |
| SYEL | 930 | 930 | 942 | 942 | 981 | 981 | 0 | 0 | 18 |
| THAF | 915 | 912 | 954 | 954 | 954 | 954 | 0 | 0 | 39 |
| THRO | 953 | 946 | 963 | 963 | 964 | 963 | 3 | 0 | 29 |
| THTH | 960 | 960 | 983 | 983 | 985 | 985 | 0 | 0 | 6 |
| THYE | 974 | 974 | 974 | 974 | 974 | 974 | 0 | 0 | 12 |
| Total | 22450 | 22389 | 23474 | 23464 | 23756 | 23746 | 27 | 0 | 1051 |

| **MetaBinX against complete NR for read length ~400 bp** | | | | | | | | | | | | | | | | | | | |
| --- | --- | --- | --- | --- | --- | --- | --- | --- | --- | --- | --- | --- | --- | --- | --- | --- | --- | --- | --- |
| **GENOME** | | **TGENUS** | | **CGENUS** | | **TFAMILY** | | **CFAMILY** | | **TPHYLUM** | | **CPHYLUM** | | **UNASS** | | **NOHITS** | | **INTERGENIC** | |
| ACFE | | 1841 | | 1833 | | 1841 | | 1833 | | 1853 | | 1849 | | 25 | | 0 | | 83 | |
| AQAE | | 1812 | | 1798 | | 1928 | | 1928 | | 1944 | | 1944 | | 10 | | 0 | | 32 | |
| ARBU | | 1826 | | 1826 | | 1839 | | 1839 | | 1881 | | 1881 | | 9 | | 0 | | 51 | |
| BOAF | | 1909 | | 1908 | | 1910 | | 1909 | | 1910 | | 1909 | | 10 | | 0 | | 33 | |
| BSUB | | 1738 | | 1738 | | 1779 | | 1779 | | 1844 | | 1844 | | 21 | | 0 | | 61 | |
| CAPH | | 1708 | | 1708 | | 1710 | | 1710 | | 1711 | | 1710 | | 70 | | 0 | | 207 | |
| CBOT | | 1733 | | 1732 | | 1736 | | 1735 | | 1762 | | 1761 | | 53 | | 0 | | 88 | |
| CFP2 | | 1217 | | 1216 | | 1252 | | 1252 | | 1264 | | 1263 | | 39 | | 0 | | 367 | |
| CHAB | | 1701 | | 1701 | | 1860 | | 1860 | | 1868 | | 1867 | | 30 | | 0 | | 91 | |
| DITH | | 1893 | | 1892 | | 1893 | | 1892 | | 1893 | | 1892 | | 7 | | 0 | | 50 | |
| ECOLI | | 194 | | 154 | | 1569 | | 1569 | | 1667 | | 1667 | | 9 | | 0 | | 148 | |
| FLJO | | 1756 | | 1756 | | 1774 | | 1774 | | 1901 | | 1900 | | 23 | | 0 | | 61 | |
| FUNU | | 1855 | | 1855 | | 1871 | | 1871 | | 1872 | | 1871 | | 11 | | 0 | | 68 | |
| GEAU | | 1924 | | 1921 | | 1924 | | 1921 | | 1924 | | 1921 | | 21 | | 0 | | 24 | |
| LACB | | 1644 | | 1644 | | 1669 | | 1669 | | 1730 | | 1730 | | 33 | | 0 | | 110 | |
| MTUB | | 1802 | | 1801 | | 1901 | | 1901 | | 1905 | | 1905 | | 9 | | 0 | | 32 | |
| PRAC | | 1834 | | 1833 | | 1834 | | 1833 | | 1888 | | 1888 | | 22 | | 0 | | 65 | |
| RHSP | | 1622 | | 1615 | | 1736 | | 1729 | | 1900 | | 1896 | | 6 | | 0 | | 49 | |
| RSD17 | | 1126 | | 1122 | | 1127 | | 1122 | | 1128 | | 1122 | | 33 | | 0 | | 452 | |
| STMU | | 1751 | | 1750 | | 1771 | | 1770 | | 1838 | | 1838 | | 28 | | 0 | | 108 | |
| SYEL | | 1739 | | 1737 | | 1760 | | 1760 | | 1869 | | 1868 | | 38 | | 0 | | 57 | |
| THAF | | 1732 | | 1730 | | 1806 | | 1805 | | 1806 | | 1805 | | 14 | | 0 | | 89 | |
| THRO | | 1824 | | 1816 | | 1861 | | 1861 | | 1868 | | 1864 | | 31 | | 0 | | 65 | |
| THTH | | 1877 | | 1875 | | 1940 | | 1939 | | 1943 | | 1942 | | 4 | | 0 | | 16 | |
| THYE | | 1903 | | 1903 | | 1903 | | 1903 | | 1905 | | 1903 | | 13 | | 0 | | 37 | |
| Total | | 41961 | | 41864 | | 44194 | | 44164 | | 45074 | | 45040 | | 569 | | 0 | | 2444 | |
| **MetaBinX against complete NR for read length ~250 bp** | | | | | | | | | | | | | | | | | | |  |
| **GENOME** | **TGENUS** | | **CGENUS** | | **TFAMILY** | | **CFAMILY** | | **TPHYLUM** | | **CPHYLUM** | | **UNASS** | | **NOHITS** | | **INTERGENIC** | |  |
| ACFE | 2634 | | 2620 | | 2634 | | 2620 | | 2665 | | 2656 | | 81 | | 0 | | 164 | |  |
| AQAE | 2538 | | 2517 | | 2784 | | 2782 | | 2819 | | 2816 | | 28 | | 0 | | 84 | |  |
| ARBU | 2727 | | 2722 | | 2741 | | 2738 | | 2854 | | 2853 | | 47 | | 0 | | 64 | |  |
| BOAF | 2888 | | 2888 | | 2894 | | 2894 | | 2896 | | 2895 | | 28 | | 0 | | 47 | |  |
| BSUB | 2508 | | 2505 | | 2585 | | 2583 | | 2723 | | 2723 | | 91 | | 0 | | 121 | |  |
| CAPH | 2444 | | 2441 | | 2449 | | 2446 | | 2450 | | 2447 | | 92 | | 0 | | 430 | |  |
| CBOT | 2515 | | 2512 | | 2523 | | 2521 | | 2580 | | 2579 | | 122 | | 0 | | 247 | |  |
| CFP2 | 2104 | | 2101 | | 2178 | | 2178 | | 2201 | | 2200 | | 98 | | 0 | | 627 | |  |
| CHAB | 2418 | | 2417 | | 2688 | | 2688 | | 2706 | | 2705 | | 86 | | 0 | | 184 | |  |
| DITH | 2803 | | 2803 | | 2803 | | 2803 | | 2803 | | 2803 | | 41 | | 0 | | 73 | |  |
| ECOLI | 314 | | 234 | | 2292 | | 2287 | | 2541 | | 2538 | | 65 | | 0 | | 268 | |  |
| FLJO | 2509 | | 2507 | | 2545 | | 2544 | | 2733 | | 2732 | | 105 | | 0 | | 122 | |  |
| FUNU | 2732 | | 2730 | | 2749 | | 2749 | | 2751 | | 2749 | | 41 | | 0 | | 109 | |  |
| GEAU | 2733 | | 2725 | | 2732 | | 2725 | | 2734 | | 2725 | | 114 | | 0 | | 64 | |  |
| LACB | 2453 | | 2452 | | 2504 | | 2503 | | 2629 | | 2627 | | 146 | | 0 | | 191 | |  |
| MTUB | 2617 | | 2613 | | 2799 | | 2799 | | 2810 | | 2807 | | 58 | | 0 | | 82 | |  |
| PRAC | 2602 | | 2597 | | 2602 | | 2597 | | 2742 | | 2742 | | 97 | | 0 | | 115 | |  |
| RHSP | 2261 | | 2246 | | 2454 | | 2440 | | 2753 | | 2747 | | 61 | | 0 | | 89 | |  |
| RSD17 | 2037 | | 2034 | | 2037 | | 2034 | | 2044 | | 2040 | | 112 | | 0 | | 775 | |  |
| STMU | 2487 | | 2485 | | 2516 | | 2514 | | 2666 | | 2666 | | 98 | | 0 | | 162 | |  |
| SYEL | 2432 | | 2430 | | 2469 | | 2469 | | 2675 | | 2673 | | 134 | | 1 | | 126 | |  |
| THAF | 2593 | | 2587 | | 2725 | | 2724 | | 2725 | | 2724 | | 43 | | 0 | | 181 | |  |
| THRO | 2572 | | 2563 | | 2652 | | 2652 | | 2671 | | 2662 | | 106 | | 0 | | 127 | |  |
| THTH | 2754 | | 2741 | | 2852 | | 2840 | | 2867 | | 2850 | | 10 | | 0 | | 36 | |  |
| THYE | 2771 | | 2768 | | 2771 | | 2768 | | 2772 | | 2768 | | 47 | | 0 | | 76 | |  |
| Total | 61446 | | 61238 | | 64978 | | 64898 | | 66810 | | 66727 | | 1951 | | 1 | | 4564 | |  |

| **MetaBinX against complete NR for read length ~75 bp** | | | | | | | | | |
| --- | --- | --- | --- | --- | --- | --- | --- | --- | --- |
| **GENOME** | **TGENUS** | **CGENUS** | **TFAMILY** | **CFAMILY** | **TPHYLUM** | **CPHYLUM** | **UNASS** | **NOHITS** | **INTERGENIC** |
| ACFE | 8596 | 8554 | 8595 | 8555 | 8626 | 8600 | 36 | 153 | 1102 |
| AQAE | 8937 | 8930 | 9075 | 9072 | 9087 | 9084 | 45 | 143 | 674 |
| ARBU | 8959 | 8954 | 8978 | 8975 | 9041 | 9038 | 48 | 195 | 688 |
| BOAF | 9145 | 9145 | 9150 | 9150 | 9150 | 9150 | 26 | 189 | 608 |
| BSUB | 8286 | 8282 | 8353 | 8349 | 8445 | 8442 | 49 | 174 | 1167 |
| CAPH | 7356 | 7352 | 7359 | 7355 | 7361 | 7356 | 50 | 218 | 2351 |
| CBOT | 7923 | 7921 | 7925 | 7923 | 7974 | 7972 | 35 | 150 | 1810 |
| CFP2 | 6753 | 6750 | 6815 | 6815 | 6836 | 6833 | 29 | 105 | 2882 |
| CHAB | 8385 | 8382 | 8614 | 8614 | 8637 | 8635 | 27 | 145 | 1169 |
| DITH | 8934 | 8930 | 8935 | 8930 | 8936 | 8930 | 50 | 171 | 662 |
| ECOLI | 982 | 905 | 7644 | 7638 | 7979 | 7972 | 12 | 110 | 1606 |
| FLJO | 8355 | 8350 | 8394 | 8390 | 8557 | 8556 | 28 | 151 | 1239 |
| FUNU | 8572 | 8569 | 8581 | 8578 | 8581 | 8578 | 21 | 163 | 1036 |
| GEAU | 8957 | 8927 | 8958 | 8927 | 8958 | 8927 | 55 | 130 | 782 |
| LACB | 8036 | 8028 | 8071 | 8063 | 8189 | 8184 | 62 | 198 | 1522 |
| MTUB | 8592 | 8551 | 8754 | 8754 | 8785 | 8762 | 38 | 241 | 818 |
| PRAC | 8627 | 8617 | 8626 | 8617 | 8714 | 8708 | 46 | 126 | 1052 |
| RHSP | 8134 | 8074 | 8380 | 8329 | 8659 | 8623 | 40 | 152 | 1046 |
| RSD17 | 6437 | 6434 | 6437 | 6434 | 6443 | 6440 | 22 | 133 | 3364 |
| STMU | 8298 | 8293 | 8328 | 8323 | 8444 | 8443 | 38 | 182 | 1296 |
| SYEL | 8320 | 8303 | 8352 | 8352 | 8543 | 8527 | 52 | 176 | 1179 |
| THAF | 8725 | 8720 | 8827 | 8826 | 8828 | 8826 | 45 | 171 | 913 |
| THRO | 8696 | 8671 | 8714 | 8714 | 8742 | 8717 | 51 | 166 | 948 |
| THTH | 9114 | 9068 | 9199 | 9155 | 9221 | 9164 | 49 | 152 | 421 |
| THYE | 9130 | 9127 | 9131 | 9128 | 9135 | 9128 | 59 | 145 | 600 |
| Total | 202249 | 201837 | 210195 | 209966 | 211871 | 211595 | 1013 | 4039 | 30935 |

| **MetaBinX against complete NR for read length ~45 bp** | | | | | | | | | |
| --- | --- | --- | --- | --- | --- | --- | --- | --- | --- |
| **GENOME** | **TGENUS** | **CGENUS** | **TFAMILY** | **CFAMILY** | **TPHYLUM** | **CPHYLUM** | **UNASS** | **NOHITS** | **INTERGENIC** |
| ACFE | 6661 | 6660 | 6662 | 6661 | 6722 | 6721 | 17 | 1803 | 1284 |
| AQAE | 7191 | 7188 | 7541 | 7540 | 7604 | 7603 | 2 | 1294 | 858 |
| ARBU | 7307 | 7305 | 7349 | 7347 | 7593 | 7592 | 2 | 1342 | 904 |
| BOAF | 7647 | 7647 | 7658 | 7658 | 7662 | 7662 | 0 | 1387 | 792 |
| BSUB | 6792 | 6792 | 6925 | 6925 | 7210 | 7210 | 7 | 1222 | 1348 |
| CAPH | 6219 | 6218 | 6229 | 6228 | 6236 | 6235 | 0 | 1106 | 2541 |
| CBOT | 6450 | 6449 | 6461 | 6460 | 6594 | 6593 | 1 | 1118 | 2090 |
| CFP2 | 5609 | 5607 | 5760 | 5760 | 5838 | 5838 | 1 | 851 | 3051 |
| CHAB | 6870 | 6870 | 7363 | 7363 | 7407 | 7407 | 0 | 1078 | 1388 |
| DITH | 7616 | 7615 | 7616 | 7615 | 7616 | 7615 | 1 | 1247 | 823 |
| ECOLI | 655 | 595 | 6051 | 6049 | 6659 | 6656 | 5 | 992 | 1901 |
| FLJO | 6943 | 6943 | 7035 | 7035 | 7423 | 7423 | 2 | 1036 | 1407 |
| FUNU | 7079 | 7079 | 7124 | 7124 | 7124 | 7124 | 2 | 1287 | 1303 |
| GEAU | 7298 | 7296 | 7298 | 7296 | 7298 | 7296 | 19 | 1506 | 970 |
| LACB | 6510 | 6510 | 6586 | 6586 | 6841 | 6841 | 8 | 1238 | 1767 |
| MTUB | 6837 | 6834 | 7238 | 7238 | 7264 | 7261 | 19 | 1478 | 1096 |
| PRAC | 6989 | 6985 | 6987 | 6985 | 7294 | 7292 | 11 | 1263 | 1251 |
| RHSP | 6011 | 6006 | 6435 | 6433 | 7139 | 7138 | 19 | 1431 | 1263 |
| RSD17 | 5348 | 5345 | 5348 | 5345 | 5363 | 5363 | 1 | 962 | 3440 |
| STMU | 6548 | 6548 | 6607 | 6607 | 6922 | 6922 | 1 | 1248 | 1607 |
| SYEL | 6370 | 6367 | 6474 | 6474 | 6922 | 6919 | 12 | 1404 | 1435 |
| THAF | 7021 | 7019 | 7277 | 7276 | 7278 | 7277 | 4 | 1284 | 1208 |
| THRO | 6828 | 6826 | 6933 | 6933 | 6951 | 6949 | 11 | 1542 | 1232 |
| THTH | 7148 | 7143 | 7375 | 7370 | 7419 | 7415 | 80 | 1559 | 685 |
| THYE | 7501 | 7501 | 7506 | 7505 | 7506 | 7505 | 1 | 1299 | 867 |
| Total | 163448 | 163348 | 171838 | 171813 | 175885 | 175857 | 226 | 31977 | 36511 |

| **MetaBinT against complete NR for read length ~800 bp** | | | | | | | | | | | | | | | | | | | |
| --- | --- | --- | --- | --- | --- | --- | --- | --- | --- | --- | --- | --- | --- | --- | --- | --- | --- | --- | --- |
| **Genome** | | TGENUS | | CGENUS | TFAMILY | | CFAMILY | | TPHYLUM | | CPHYLUM | | UNASS | | NOHITS | | INTERGENIC | |  |
| ACFE | | 964 | | 964 | 964 | | 964 | | 968 | | 968 | | 2 | | 1 | | 23 | |  |
| AQAE | | 936 | | 933 | 978 | | 978 | | 983 | | 982 | | 1 | | 0 | | 9 | |  |
| ARBU | | 967 | | 967 | 969 | | 969 | | 987 | | 987 | | 0 | | 0 | | 11 | |  |
| BOAF | | 975 | | 975 | 975 | | 975 | | 975 | | 975 | | 0 | | 0 | | 19 | |  |
| BSUB | | 919 | | 919 | 941 | | 941 | | 958 | | 958 | | 3 | | 1 | | 36 | |  |
| CAPH | | 886 | | 886 | 886 | | 886 | | 886 | | 886 | | 1 | | 5 | | 105 | |  |
| CBOT | | 927 | | 927 | 931 | | 931 | | 938 | | 938 | | 0 | | 0 | | 58 | |  |
| CFP2 | | 828 | | 828 | 844 | | 844 | | 845 | | 845 | | 1 | | 1 | | 146 | |  |
| CHAB | | 883 | | 883 | 953 | | 953 | | 954 | | 954 | | 1 | | 0 | | 38 | |  |
| DITH | | 962 | | 962 | 962 | | 962 | | 962 | | 962 | | 0 | | 0 | | 26 | |  |
| ECOLI | | 161 | | 124 | 885 | | 883 | | 929 | | 927 | | 0 | | 0 | | 54 | |  |
| FLJO | | 909 | | 908 | 914 | | 914 | | 955 | | 955 | | 2 | | 0 | | 36 | |  |
| FUNU | | 956 | | 955 | 961 | | 960 | | 961 | | 960 | | 0 | | 0 | | 22 | |  |
| GEAU | | 979 | | 978 | 979 | | 978 | | 979 | | 978 | | 1 | | 0 | | 10 | |  |
| LACB | | 931 | | 931 | 941 | | 941 | | 951 | | 951 | | 3 | | 1 | | 43 | |  |
| MTUB | | 951 | | 951 | 977 | | 977 | | 977 | | 977 | | 0 | | 0 | | 20 | |  |
| PRAC | | 945 | | 945 | 945 | | 945 | | 960 | | 960 | | 1 | | 0 | | 35 | |  |
| RHSP | | 890 | | 889 | 916 | | 915 | | 970 | | 970 | | 0 | | 0 | | 29 | |  |
| RSD17 | | 788 | | 788 | 789 | | 788 | | 790 | | 788 | | 1 | | 3 | | 202 | |  |
| STMU | | 931 | | 930 | 942 | | 941 | | 966 | | 966 | | 1 | | 0 | | 25 | |  |
| SYEL | | 931 | | 930 | 939 | | 939 | | 979 | | 979 | | 0 | | 0 | | 18 | |  |
| THAF | | 915 | | 912 | 952 | | 952 | | 952 | | 952 | | 0 | | 0 | | 39 | |  |
| THRO | | 950 | | 947 | 960 | | 960 | | 960 | | 960 | | 2 | | 2 | | 29 | |  |
| THTH | | 980 | | 980 | 984 | | 984 | | 984 | | 984 | | 0 | | 0 | | 6 | |  |
| THYE | | 972 | | 971 | 972 | | 971 | | 972 | | 971 | | 0 | | 0 | | 12 | |  |
| Total | | 22436 | | 22383 | 23459 | | 23451 | | 23741 | | 23733 | | 20 | | 14 | | 1051 | |  |
| **MetaBinT against complete NR for read length ~400 bp** | | | | | | | | | | | | | | | | | |  | |
| **GENOME** | **TGENUS** | | **CGENUS** | | **TFAMILY** | **CFAMILY** | | **TPHYLUM** | | **CPHYLUM** | | **UNASS** | | **NOHITS** | | **INTERGENIC** | |  | |
| ACFE | 1764 | | 1763 | | 1764 | 1763 | | 1775 | | 1772 | | 73 | | 11501 | | 83 | |  | |
| AQAE | 1710 | | 1693 | | 1857 | 1853 | | 1877 | | 1871 | | 58 | | 11803 | | 32 | |  | |
| ARBU | 1719 | | 1714 | | 1733 | 1727 | | 1808 | | 1807 | | 73 | | 11344 | | 51 | |  | |
| BOAF | 1840 | | 1839 | | 1840 | 1839 | | 1841 | | 1839 | | 64 | | 11565 | | 33 | |  | |
| BSUB | 1620 | | 1616 | | 1670 | 1668 | | 1758 | | 1755 | | 71 | | 11274 | | 61 | |  | |
| CAPH | 1556 | | 1553 | | 1559 | 1556 | | 1559 | | 1556 | | 186 | | 10661 | | 207 | |  | |
| CBOT | 1627 | | 1625 | | 1632 | 1630 | | 1670 | | 1670 | | 79 | | 10860 | | 88 | |  | |
| CFP2 | 1131 | | 1127 | | 1181 | 1181 | | 1206 | | 1204 | | 53 | | 7957 | | 367 | |  | |
| CHAB | 1618 | | 1615 | | 1794 | 1794 | | 1810 | | 1808 | | 54 | | 11434 | | 91 | |  | |
| DITH | 1826 | | 1821 | | 1826 | 1821 | | 1827 | | 1821 | | 48 | | 11672 | | 50 | |  | |
| ECOLI | 204 | | 148 | | 1457 | 1451 | | 1590 | | 1586 | | 44 | | 10318 | | 148 | |  | |
| FLJO | 1640 | | 1640 | | 1666 | 1666 | | 1816 | | 1816 | | 67 | | 11611 | | 61 | |  | |
| FUNU | 1743 | | 1739 | | 1756 | 1753 | | 1761 | | 1753 | | 69 | | 11535 | | 68 | |  | |
| GEAU | 1846 | | 1846 | | 1846 | 1846 | | 1846 | | 1846 | | 67 | | 11855 | | 24 | |  | |
| LACB | 1542 | | 1539 | | 1566 | 1563 | | 1664 | | 1663 | | 76 | | 10691 | | 110 | |  | |
| MTUB | 1715 | | 1714 | | 1836 | 1836 | | 1842 | | 1842 | | 56 | | 11615 | | 32 | |  | |
| PRAC | 1747 | | 1741 | | 1747 | 1741 | | 1820 | | 1819 | | 64 | | 11609 | | 65 | |  | |
| RHSP | 1504 | | 1494 | | 1637 | 1631 | | 1825 | | 1824 | | 82 | | 11705 | | 49 | |  | |
| RSD17 | 1059 | | 1056 | | 1060 | 1056 | | 1064 | | 1059 | | 59 | | 7136 | | 452 | |  | |
| STMU | 1624 | | 1620 | | 1653 | 1650 | | 1741 | | 1740 | | 82 | | 11323 | | 108 | |  | |
| SYEL | 1595 | | 1593 | | 1622 | 1622 | | 1758 | | 1758 | | 107 | | 11657 | | 57 | |  | |
| THAF | 1650 | | 1644 | | 1740 | 1738 | | 1742 | | 1739 | | 53 | | 10959 | | 89 | |  | |
| THRO | 1710 | | 1708 | | 1760 | 1760 | | 1770 | | 1767 | | 83 | | 11609 | | 65 | |  | |
| THTH | 1852 | | 1850 | | 1855 | 1853 | | 1866 | | 1864 | | 69 | | 11903 | | 16 | |  | |
| THYE | 1839 | | 1839 | | 1839 | 1839 | | 1842 | | 1839 | | 44 | | 11753 | | 37 | |  | |
| Total | 39681 | | 39537 | | 41896 | 41837 | | 43078 | | 43018 | | 1781 | | 277350 | | 2444 | |  | |

| **MetaBinT against complete NR for read length ~250 bp** | | | | | | | | | |
| --- | --- | --- | --- | --- | --- | --- | --- | --- | --- |
| **GENOME** | **TGENUS** | **CGENUS** | **TFAMILY** | **CFAMILY** | **TPHYLUM** | **CPHYLUM** | **UNASS** | **NOHITS** | **INTERGENIC** |
| ACFE | 2619 | 2616 | 2619 | 2616 | 2645 | 2643 | 9 | 0 | 164 |
| AQAE | 2505 | 2485 | 2747 | 2743 | 2790 | 2786 | 7 | 0 | 84 |
| ARBU | 2663 | 2658 | 2692 | 2686 | 2809 | 2808 | 21 | 0 | 64 |
| BOAF | 2848 | 2846 | 2852 | 2851 | 2854 | 2853 | 11 | 0 | 47 |
| BSUB | 2450 | 2444 | 2522 | 2517 | 2673 | 2670 | 23 | 0 | 121 |
| CAPH | 2379 | 2379 | 2385 | 2385 | 2392 | 2389 | 53 | 0 | 430 |
| CBOT | 2479 | 2477 | 2486 | 2484 | 2549 | 2548 | 21 | 0 | 247 |
| CFP2 | 2032 | 2025 | 2144 | 2144 | 2198 | 2196 | 13 | 0 | 627 |
| CHAB | 2387 | 2382 | 2655 | 2652 | 2675 | 2673 | 14 | 0 | 184 |
| DITH | 2740 | 2738 | 2740 | 2738 | 2741 | 2738 | 24 | 0 | 73 |
| ECOLI | 323 | 235 | 2200 | 2192 | 2477 | 2472 | 12 | 0 | 268 |
| FLJO | 2502 | 2497 | 2531 | 2528 | 2726 | 2722 | 8 | 0 | 122 |
| FUNU | 2657 | 2655 | 2686 | 2685 | 2688 | 2685 | 14 | 0 | 109 |
| GEAU | 2723 | 2722 | 2723 | 2722 | 2724 | 2722 | 19 | 0 | 64 |
| LACB | 2445 | 2442 | 2498 | 2496 | 2624 | 2624 | 12 | 0 | 191 |
| MTUB | 2604 | 2596 | 2789 | 2789 | 2800 | 2798 | 8 | 0 | 82 |
| PRAC | 2585 | 2580 | 2585 | 2580 | 2709 | 2705 | 27 | 0 | 115 |
| RHSP | 2237 | 2224 | 2436 | 2427 | 2742 | 2741 | 16 | 0 | 89 |
| RSD17 | 2003 | 2000 | 2003 | 2000 | 2010 | 2006 | 14 | 0 | 775 |
| STMU | 2445 | 2438 | 2476 | 2470 | 2640 | 2638 | 17 | 0 | 162 |
| SYEL | 2379 | 2369 | 2411 | 2411 | 2638 | 2631 | 22 | 0 | 126 |
| THAF | 2517 | 2512 | 2676 | 2675 | 2679 | 2675 | 12 | 0 | 181 |
| THRO | 2548 | 2546 | 2621 | 2621 | 2628 | 2628 | 12 | 0 | 127 |
| THTH | 2804 | 2797 | 2807 | 2799 | 2829 | 2817 | 8 | 0 | 36 |
| THYE | 2711 | 2710 | 2712 | 2711 | 2714 | 2711 | 18 | 0 | 76 |
| Total | 60585 | 60373 | 63996 | 63922 | 65954 | 65879 | 415 | 0 | 4564 |

| **MetaBinT against complete NR for read length ~75 bp** | | | | | | | | | |
| --- | --- | --- | --- | --- | --- | --- | --- | --- | --- |
| GENOME | TGENUS | CGENUS | TFAMILY | CFAMILY | TPHYLUM | CPHYLUM | UNASS | NOHITS | INTERGENIC |
| ACFE | 8719 | 8669 | 8719 | 8670 | 8750 | 8714 | 58 | 30 | 1102 |
| AQAE | 8965 | 8952 | 9141 | 9130 | 9158 | 9146 | 7 | 109 | 674 |
| ARBU | 8960 | 8952 | 8978 | 8970 | 9061 | 9053 | 15 | 204 | 688 |
| BOAF | 9183 | 9170 | 9189 | 9176 | 9191 | 9177 | 11 | 157 | 608 |
| BSUB | 8453 | 8447 | 8537 | 8532 | 8645 | 8640 | 12 | 127 | 1167 |
| CAPH | 7404 | 7394 | 7407 | 7397 | 7408 | 7398 | 26 | 193 | 2351 |
| CBOT | 7880 | 7871 | 7883 | 7876 | 7934 | 7924 | 10 | 209 | 1810 |
| CFP2 | 6846 | 6835 | 6926 | 6926 | 6967 | 6956 | 7 | 101 | 2882 |
| CHAB | 8402 | 8396 | 8666 | 8660 | 8688 | 8682 | 4 | 109 | 1169 |
| DITH | 9080 | 9066 | 9078 | 9066 | 9082 | 9066 | 10 | 166 | 662 |
| ECOLI | 975 | 898 | 7667 | 7659 | 8032 | 8028 | 6 | 72 | 1606 |
| FLJO | 8346 | 8342 | 8381 | 8377 | 8572 | 8569 | 12 | 153 | 1239 |
| FUNU | 8668 | 8654 | 8685 | 8670 | 8685 | 8670 | 19 | 175 | 1036 |
| GEAU | 9084 | 9069 | 9084 | 9069 | 9087 | 9069 | 21 | 48 | 782 |
| LACB | 8118 | 8114 | 8161 | 8157 | 8295 | 8291 | 10 | 140 | 1522 |
| MTUB | 8878 | 8857 | 9055 | 9055 | 9080 | 9067 | 16 | 41 | 818 |
| PRAC | 8718 | 8705 | 8717 | 8705 | 8820 | 8813 | 13 | 68 | 1052 |
| RHSP | 8232 | 8194 | 8503 | 8472 | 8833 | 8817 | 38 | 30 | 1046 |
| RSD17 | 6470 | 6461 | 6470 | 6461 | 6474 | 6465 | 10 | 102 | 3364 |
| STMU | 8301 | 8295 | 8331 | 8325 | 8469 | 8466 | 20 | 163 | 1296 |
| SYEL | 8407 | 8393 | 8444 | 8444 | 8663 | 8653 | 18 | 99 | 1179 |
| THAF | 8726 | 8715 | 8846 | 8842 | 8846 | 8842 | 8 | 180 | 913 |
| THRO | 8845 | 8822 | 8862 | 8862 | 8886 | 8866 | 31 | 56 | 948 |
| THTH | 9347 | 9315 | 9351 | 9323 | 9377 | 9338 | 67 | 41 | 421 |
| THYE | 9165 | 9155 | 9165 | 9156 | 9167 | 9156 | 14 | 153 | 600 |
| Total | 204172 | 203741 | 212246 | 211980 | 214170 | 213866 | 463 | 2926 | 30935 |

| **MetaBinT against complete NR for read length ~45 bp** | | | | | | | | | |
| --- | --- | --- | --- | --- | --- | --- | --- | --- | --- |
| **GENOME** | **TGENUS** | **CGENUS** | **TFAMILY** | **CFAMILY** | **TPHYLUM** | **CPHYLUM** | **UNASS** | **NOHITS** | **INTERGENIC** |
| ACFE | 7694 | 7592 | 7698 | 7593 | 7790 | 7718 | 80 | 626 | 1284 |
| AQAE | 8001 | 7974 | 8422 | 8404 | 8507 | 8486 | 14 | 310 | 858 |
| ARBU | 8297 | 8277 | 8351 | 8337 | 8665 | 8656 | 4 | 229 | 904 |
| BOAF | 8796 | 8791 | 8809 | 8803 | 8814 | 8807 | 5 | 194 | 792 |
| BSUB | 7634 | 7615 | 7784 | 7769 | 8123 | 8110 | 5 | 247 | 1348 |
| CAPH | 7108 | 7098 | 7124 | 7113 | 7135 | 7123 | 2 | 176 | 2541 |
| CBOT | 7276 | 7265 | 7287 | 7276 | 7449 | 7443 | 2 | 191 | 2090 |
| CFP2 | 6200 | 6188 | 6410 | 6410 | 6538 | 6532 | 4 | 142 | 3051 |
| CHAB | 7603 | 7592 | 8196 | 8190 | 8252 | 8245 | 2 | 206 | 1388 |
| DITH | 8524 | 8503 | 8525 | 8503 | 8527 | 8503 | 3 | 252 | 823 |
| ECOLI | 737 | 642 | 6713 | 6704 | 7442 | 7435 | 3 | 175 | 1901 |
| FLJO | 7691 | 7687 | 7759 | 7754 | 8252 | 8246 | 6 | 179 | 1407 |
| FUNU | 8039 | 8030 | 8101 | 8091 | 8102 | 8091 | 5 | 217 | 1303 |
| GEAU | 8291 | 8249 | 8291 | 8249 | 8297 | 8249 | 31 | 441 | 970 |
| LACB | 7354 | 7339 | 7450 | 7437 | 7770 | 7759 | 8 | 284 | 1767 |
| MTUB | 7882 | 7836 | 8310 | 8310 | 8368 | 8337 | 33 | 314 | 1096 |
| PRAC | 7809 | 7775 | 7806 | 7775 | 8172 | 8149 | 29 | 331 | 1251 |
| RHSP | 6809 | 6713 | 7291 | 7219 | 8128 | 8094 | 64 | 357 | 1263 |
| RSD17 | 6057 | 6046 | 6058 | 6046 | 6083 | 6067 | 3 | 183 | 3440 |
| STMU | 7425 | 7407 | 7505 | 7488 | 7880 | 7874 | 12 | 204 | 1607 |
| SYEL | 7250 | 7224 | 7358 | 7358 | 7907 | 7890 | 22 | 362 | 1435 |
| THAF | 7945 | 7922 | 8266 | 8250 | 8271 | 8251 | 6 | 234 | 1208 |
| THRO | 7691 | 7640 | 7784 | 7784 | 7849 | 7804 | 21 | 558 | 1232 |
| THTH | 8194 | 8077 | 8204 | 8089 | 8264 | 8142 | 112 | 548 | 685 |
| THYE | 8398 | 8374 | 8403 | 8378 | 8409 | 8378 | 9 | 298 | 867 |
| Total | 184705 | 183856 | 193905 | 193330 | 198994 | 198389 | 485 | 7258 | 36511 |

| **MetaBinX against NRminusGenus for read length ~800 bp** | | | | | | | |
| --- | --- | --- | --- | --- | --- | --- | --- |
| GENOME | TFAMILY | CFAMILY | TPHYLUM | CPHYLUM | UNASS | NOHITS | INTERGENIC |
| ACFE | 159 | 0 | 377 | 283 | 66 | 0 | 23 |
| AQAE | 736 | 687 | 839 | 818 | 34 | 0 | 9 |
| ARBU | 170 | 72 | 726 | 698 | 86 | 0 | 11 |
| BOAF | 191 | 141 | 255 | 163 | 224 | 0 | 19 |
| BSUB | 342 | 222 | 716 | 686 | 80 | 0 | 36 |
| CBOT | 202 | 37 | 461 | 410 | 129 | 0 | 58 |
| CFP2 | 418 | 418 | 557 | 548 | 48 | 0 | 146 |
| CHAB | 811 | 805 | 832 | 826 | 106 | 0 | 38 |
| ECOLI | 872 | 866 | 924 | 921 | 2 | 0 | 54 |
| FLJO | 215 | 106 | 767 | 727 | 88 | 0 | 36 |
| FUNU | 360 | 211 | 475 | 211 | 86 | 0 | 22 |
| LACB | 294 | 130 | 712 | 695 | 107 | 0 | 43 |
| MTUB | 661 | 661 | 749 | 699 | 72 | 0 | 20 |
| PRAC | 180 | 0 | 676 | 651 | 101 | 0 | 35 |
| RHSP | 615 | 562 | 885 | 878 | 13 | 0 | 29 |
| STMU | 305 | 119 | 718 | 685 | 73 | 0 | 25 |
| SYEL | 62 | 62 | 793 | 745 | 63 | 0 | 18 |
| THAF | 713 | 693 | 732 | 695 | 72 | 0 | 39 |
| THRO | 494 | 494 | 612 | 537 | 71 | 0 | 29 |
| THTH | 594 | 548 | 686 | 617 | 15 | 0 | 6 |
| THYE | 157 | 1 | 293 | 1 | 84 | 0 | 12 |
| 800 | 8551 | 6835 | 13785 | 12494 | 1620 | 0 | 708 |

| **MetaBinX against NRminusGenus for read length ~400 bp** | | | | | | | |
| --- | --- | --- | --- | --- | --- | --- | --- |
| GENOME | TFAMILY | CFAMILY | TPHYLUM | CPHYLUM | UNASS | NOHITS | INTERGENIC |
| ACFE | 360 | 0 | 639 | 434 | 364 | 0 | 83 |
| AQAE | 1200 | 1126 | 1394 | 1349 | 295 | 0 | 32 |
| ARBU | 296 | 116 | 1076 | 1037 | 550 | 0 | 51 |
| BOAF | 211 | 112 | 283 | 134 | 1011 | 0 | 33 |
| BSUB | 497 | 321 | 1055 | 1010 | 494 | 0 | 61 |
| CBOT | 303 | 53 | 644 | 547 | 613 | 0 | 88 |
| CFP2 | 468 | 468 | 723 | 700 | 250 | 0 | 367 |
| CHAB | 1254 | 1236 | 1300 | 1284 | 498 | 0 | 91 |
| ECOLI | 1508 | 1503 | 1635 | 1634 | 45 | 0 | 148 |
| FLJO | 368 | 178 | 1206 | 1164 | 544 | 0 | 61 |
| FUNU | 519 | 268 | 650 | 268 | 586 | 0 | 68 |
| LACB | 409 | 195 | 998 | 976 | 536 | 0 | 110 |
| MTUB | 1030 | 1030 | 1164 | 1072 | 435 | 0 | 32 |
| PRAC | 301 | 0 | 979 | 929 | 594 | 0 | 65 |
| RHSP | 945 | 816 | 1489 | 1416 | 127 | 0 | 49 |
| STMU | 453 | 160 | 1035 | 971 | 483 | 0 | 108 |
| SYEL | 107 | 107 | 1133 | 1067 | 479 | 0 | 57 |
| THAF | 1011 | 968 | 1035 | 972 | 452 | 0 | 89 |
| THRO | 574 | 574 | 813 | 646 | 447 | 0 | 65 |
| THTH | 929 | 776 | 1098 | 876 | 101 | 0 | 16 |
| THYE | 295 | 1 | 501 | 1 | 624 | 0 | 37 |
| Total | 13038 | 10008 | 20850 | 18487 | 9528 | 0 | 1711 |

| **MetaBinX against NRminusGenus for read length ~250 bp** | | | | | | | |
| --- | --- | --- | --- | --- | --- | --- | --- |
| **GENOME** | **TFAMILY** | **CFAMILY** | **TPHYLUM** | **CPHYLUM** | **UNASS** | **NOHITS** | **INTERGENIC** |
| ACFE | 442 | 0 | 753 | 472 | 950 | 0 | 164 |
| AQAE | 1616 | 1515 | 1845 | 1786 | 542 | 0 | 84 |
| ARBU | 388 | 126 | 1397 | 1328 | 1073 | 0 | 64 |
| BOAF | 289 | 127 | 384 | 148 | 1761 | 0 | 47 |
| BSUB | 580 | 367 | 1271 | 1212 | 1078 | 0 | 121 |
| CBOT | 387 | 49 | 770 | 658 | 1168 | 1 | 247 |
| CFP2 | 738 | 738 | 1139 | 1105 | 583 | 0 | 627 |
| CHAB | 1567 | 1550 | 1656 | 1635 | 1148 | 1 | 184 |
| ECOLI | 2178 | 2157 | 2453 | 2443 | 135 | 0 | 268 |
| FLJO | 422 | 217 | 1512 | 1452 | 1066 | 0 | 122 |
| FUNU | 605 | 287 | 802 | 287 | 1156 | 1 | 109 |
| LACB | 473 | 215 | 1212 | 1172 | 1181 | 0 | 191 |
| MTUB | 1352 | 1352 | 1553 | 1409 | 888 | 0 | 82 |
| PRAC | 334 | 0 | 1115 | 1029 | 1181 | 0 | 115 |
| RHSP | 1320 | 1097 | 1996 | 1894 | 376 | 0 | 89 |
| STMU | 549 | 174 | 1248 | 1185 | 1021 | 1 | 162 |
| SYEL | 126 | 126 | 1369 | 1276 | 1078 | 2 | 126 |
| THAF | 1327 | 1250 | 1362 | 1253 | 887 | 0 | 181 |
| THRO | 631 | 631 | 935 | 716 | 1015 | 0 | 127 |
| THTH | 1233 | 946 | 1508 | 1084 | 153 | 0 | 36 |
| THYE | 330 | 4 | 558 | 4 | 1197 | 0 | 76 |
| Total | 16887 | 12928 | 26838 | 23548 | 19637 | 6 | 3222 |

| **MetaBinX against NRminusGenus for read length ~75 bp** | | | | | | | |
| --- | --- | --- | --- | --- | --- | --- | --- |
| **GENOME** | **TFAMILY** | **CFAMILY** | **TPHYLUM** | **CPHYLUM** | **UNASS** | **NOHITS** | **INTERGENIC** |
| ACFE | 1383 | 1 | 2318 | 1315 | 277 | 2919 | 1102 |
| AQAE | 4034 | 3542 | 4579 | 4174 | 234 | 2400 | 674 |
| ARBU | 1412 | 441 | 3837 | 3581 | 148 | 3649 | 688 |
| BOAF | 810 | 283 | 1104 | 344 | 250 | 5510 | 608 |
| BSUB | 2192 | 1187 | 3972 | 3634 | 221 | 2741 | 1167 |
| CBOT | 1337 | 170 | 2319 | 1872 | 230 | 3110 | 1810 |
| CFP2 | 1976 | 1976 | 3241 | 3009 | 110 | 1599 | 2882 |
| CHAB | 4518 | 4375 | 4759 | 4615 | 168 | 2993 | 1169 |
| ECOLI | 7411 | 7349 | 7780 | 7743 | 39 | 260 | 1606 |
| FLJO | 1481 | 714 | 4299 | 4009 | 179 | 3085 | 1239 |
| FUNU | 1821 | 693 | 2388 | 693 | 167 | 3492 | 1036 |
| LACB | 1937 | 691 | 3722 | 3471 | 135 | 3058 | 1522 |
| MTUB | 3785 | 3785 | 4552 | 3955 | 365 | 2395 | 818 |
| PRAC | 1616 | 2 | 3565 | 3034 | 274 | 2981 | 1052 |
| RHSP | 4519 | 3716 | 6341 | 6080 | 200 | 1196 | 1046 |
| STMU | 1982 | 524 | 3778 | 3468 | 174 | 2859 | 1296 |
| SYEL | 706 | 706 | 4357 | 3874 | 203 | 2535 | 1179 |
| THAF | 3531 | 3158 | 3667 | 3162 | 162 | 3145 | 913 |
| THRO | 1664 | 1664 | 2865 | 1889 | 242 | 2575 | 948 |
| THTH | 3660 | 2589 | 4244 | 2934 | 324 | 1152 | 421 |
| THYE | 1216 | 17 | 1945 | 17 | 185 | 3751 | 600 |
| Total | 52991 | 37583 | 79632 | 66873 | 4287 | 57405 | 23776 |

| **MetaBinX against NRminusGenus for read length ~45 bp** | | | | | | | |
| --- | --- | --- | --- | --- | --- | --- | --- |
| **GENOME** | **TFAMILY** | **CFAMILY** | **TPHYLUM** | **CPHYLUM** | **UNASS** | **NOHITS** | **INTERGENIC** |
| ACFE | 336 | 1 | 480 | 225 | 209 | 7759 | 1284 |
| AQAE | 1285 | 1008 | 1415 | 1174 | 453 | 6912 | 858 |
| ARBU | 513 | 155 | 968 | 872 | 199 | 7687 | 904 |
| BOAF | 200 | 39 | 269 | 54 | 255 | 8424 | 792 |
| BSUB | 841 | 365 | 1215 | 1039 | 404 | 6707 | 1348 |
| CBOT | 483 | 38 | 656 | 465 | 212 | 6739 | 2090 |
| CFP2 | 538 | 538 | 946 | 784 | 238 | 5349 | 3051 |
| CHAB | 1269 | 1171 | 1335 | 1251 | 273 | 6825 | 1388 |
| ECOLI | 5638 | 5606 | 6261 | 6238 | 41 | 1340 | 1901 |
| FLJO | 539 | 264 | 1267 | 1157 | 273 | 6839 | 1407 |
| FUNU | 544 | 151 | 661 | 151 | 215 | 7474 | 1303 |
| LACB | 721 | 261 | 1010 | 931 | 205 | 6800 | 1767 |
| MTUB | 1000 | 1000 | 1162 | 1037 | 421 | 7130 | 1096 |
| PRAC | 645 | 0 | 1020 | 858 | 327 | 7138 | 1251 |
| RHSP | 1681 | 1413 | 2215 | 2187 | 374 | 5946 | 1263 |
| STMU | 789 | 163 | 1135 | 994 | 274 | 6656 | 1607 |
| SYEL | 343 | 343 | 1426 | 1220 | 287 | 6529 | 1435 |
| THAF | 952 | 764 | 1013 | 765 | 256 | 7227 | 1208 |
| THRO | 377 | 377 | 783 | 440 | 285 | 7333 | 1232 |
| THTH | 1064 | 676 | 1174 | 777 | 885 | 6900 | 685 |
| THYE | 472 | 7 | 656 | 7 | 249 | 7824 | 867 |
| Total | 20230 | 14340 | 27067 | 22626 | 6335 | 141538 | 28737 |

| **MetaBinT against NRminusGenus for read length ~800 bp** | | | | | | | |
| --- | --- | --- | --- | --- | --- | --- | --- |
| **GENOME** | **TFAMILY** | **CFAMILY** | **TPHYLUM** | **CPHYLUM** | **UNASS** | **NOHITS** | **INTERGENIC** |
| ACFE | 162 | 0 | 302 | 221 | 112 | 315 | 23 |
| AQAE | 691 | 620 | 789 | 744 | 46 | 78 | 9 |
| ARBU | 222 | 72 | 616 | 590 | 64 | 198 | 11 |
| BOAF | 165 | 79 | 218 | 84 | 99 | 386 | 19 |
| BSUB | 366 | 234 | 628 | 592 | 40 | 157 | 36 |
| CBOT | 258 | 42 | 458 | 380 | 55 | 217 | 58 |
| CFP2 | 458 | 458 | 626 | 610 | 36 | 81 | 146 |
| CHAB | 730 | 724 | 752 | 745 | 28 | 155 | 38 |
| ECOLI | 862 | 856 | 914 | 910 | 3 | 2 | 54 |
| FLJO | 242 | 106 | 660 | 627 | 64 | 184 | 36 |
| FUNU | 355 | 160 | 430 | 160 | 67 | 200 | 22 |
| LACB | 346 | 145 | 643 | 620 | 47 | 177 | 43 |
| MTUB | 568 | 568 | 650 | 593 | 108 | 142 | 20 |
| PRAC | 234 | 0 | 594 | 545 | 63 | 184 | 35 |
| RHSP | 628 | 563 | 841 | 835 | 29 | 61 | 29 |
| STMU | 359 | 118 | 641 | 605 | 50 | 140 | 25 |
| SYEL | 93 | 93 | 699 | 654 | 53 | 113 | 18 |
| THAF | 603 | 552 | 612 | 553 | 54 | 168 | 39 |
| THRO | 420 | 420 | 548 | 461 | 73 | 150 | 29 |
| THTH | 338 | 6 | 402 | 200 | 174 | 70 | 6 |
| THYE | 188 | 3 | 327 | 3 | 73 | 191 | 12 |
| 800 | 8288 | 5819 | 12350 | 10732 | 1338 | 3369 | 708 |

| **MetaBinT against NRminusGenus for read length ~400 bp** | | | | | | | |
| --- | --- | --- | --- | --- | --- | --- | --- |
| **GENOME** | **TFAMILY** | **CFAMILY** | **TPHYLUM** | **CPHYLUM** | **UNASS** | **NOHITS** | **INTERGENIC** |
| ACFE | 133 | 0 | 256 | 160 | 173 | 1222 | 83 |
| AQAE | 839 | 753 | 957 | 898 | 176 | 582 | 32 |
| ARBU | 218 | 74 | 645 | 608 | 179 | 912 | 51 |
| BOAF | 125 | 47 | 166 | 56 | 178 | 1324 | 33 |
| BSUB | 324 | 179 | 651 | 597 | 205 | 804 | 61 |
| CBOT | 212 | 25 | 388 | 314 | 148 | 981 | 88 |
| CFP2 | 336 | 336 | 551 | 531 | 106 | 455 | 367 |
| CHAB | 806 | 790 | 849 | 836 | 160 | 804 | 91 |
| ECOLI | 1383 | 1374 | 1537 | 1532 | 64 | 47 | 148 |
| FLJO | 239 | 97 | 743 | 716 | 170 | 897 | 61 |
| FUNU | 302 | 124 | 398 | 124 | 194 | 992 | 68 |
| LACB | 325 | 139 | 650 | 631 | 128 | 856 | 110 |
| MTUB | 646 | 646 | 726 | 673 | 213 | 858 | 32 |
| PRAC | 228 | 0 | 580 | 522 | 209 | 944 | 65 |
| RHSP | 742 | 679 | 1101 | 1097 | 172 | 581 | 49 |
| STMU | 333 | 93 | 660 | 619 | 197 | 792 | 108 |
| SYEL | 96 | 96 | 728 | 684 | 204 | 789 | 57 |
| THAF | 615 | 574 | 636 | 575 | 155 | 803 | 89 |
| THRO | 324 | 324 | 451 | 362 | 167 | 961 | 65 |
| THTH | 265 | 8 | 328 | 131 | 624 | 599 | 16 |
| THYE | 185 | 1 | 307 | 1 | 194 | 1041 | 37 |
| Total | 8676 | 6359 | 13308 | 11667 | 4016 | 17244 | 1711 |

| **MetaBinT against NRminusGenus for read length ~250 bp** | | | | | | | |
| --- | --- | --- | --- | --- | --- | --- | --- |
| **GENOME** | **TFAMILY** | **CFAMILY** | **TPHYLUM** | **CPHYLUM** | **UNASS** | **NOHITS** | **INTERGENIC** |
| ACFE | 178 | 0 | 342 | 217 | 43 | 2074 | 164 |
| AQAE | 1050 | 946 | 1218 | 1137 | 85 | 1228 | 84 |
| ARBU | 299 | 99 | 843 | 796 | 53 | 1807 | 64 |
| BOAF | 178 | 64 | 235 | 80 | 66 | 2330 | 47 |
| BSUB | 456 | 278 | 891 | 836 | 108 | 1567 | 121 |
| CBOT | 289 | 44 | 511 | 418 | 61 | 1796 | 247 |
| CFP2 | 596 | 596 | 940 | 886 | 51 | 1081 | 627 |
| CHAB | 1034 | 999 | 1090 | 1056 | 76 | 1518 | 184 |
| ECOLI | 2075 | 2056 | 2366 | 2355 | 30 | 149 | 268 |
| FLJO | 273 | 116 | 944 | 900 | 85 | 1677 | 122 |
| FUNU | 383 | 158 | 525 | 158 | 58 | 1857 | 109 |
| LACB | 425 | 186 | 811 | 789 | 53 | 1709 | 191 |
| MTUB | 893 | 893 | 998 | 936 | 129 | 1631 | 82 |
| PRAC | 322 | 0 | 776 | 700 | 86 | 1768 | 115 |
| RHSP | 1042 | 934 | 1454 | 1446 | 72 | 1235 | 89 |
| STMU | 441 | 120 | 864 | 801 | 93 | 1549 | 162 |
| SYEL | 124 | 124 | 999 | 927 | 72 | 1524 | 126 |
| THAF | 825 | 762 | 856 | 764 | 58 | 1587 | 181 |
| THRO | 364 | 364 | 548 | 417 | 52 | 1808 | 127 |
| THTH | 435 | 5 | 523 | 189 | 576 | 1304 | 36 |
| THYE | 286 | 4 | 445 | 4 | 70 | 1898 | 76 |
| Total | 11968 | 8748 | 18179 | 15812 | 1977 | 33097 | 3222 |

| **MetaBinT against NRminusGenus for read length ~75 bp** | | | | | | | |
| --- | --- | --- | --- | --- | --- | --- | --- |
| **GENOME** | **TFAMILY** | **CFAMILY** | **TPHYLUM** | **CPHYLUM** | **UNASS** | **NOHITS** | **INTERGENIC** |
| ACFE | 2349 | 1 | 3101 | 1361 | 2254 | 2105 | 1102 |
| AQAE | 3665 | 2682 | 4108 | 3147 | 1052 | 3050 | 674 |
| ARBU | 1793 | 466 | 3432 | 2889 | 983 | 4171 | 688 |
| BOAF | 1257 | 170 | 1617 | 233 | 1131 | 5448 | 608 |
| BSUB | 2430 | 1062 | 3616 | 2965 | 998 | 3292 | 1167 |
| CBOT | 1638 | 163 | 2344 | 1624 | 967 | 3707 | 1810 |
| CFP2 | 1996 | 1996 | 3319 | 2857 | 669 | 2176 | 2882 |
| CHAB | 3550 | 3106 | 3735 | 3267 | 904 | 3728 | 1169 |
| ECOLI | 7423 | 7352 | 7806 | 7764 | 67 | 232 | 1606 |
| FLJO | 1515 | 519 | 3436 | 2871 | 973 | 3852 | 1239 |
| FUNU | 2028 | 566 | 2561 | 566 | 1041 | 4027 | 1036 |
| LACB | 2248 | 653 | 3319 | 2806 | 890 | 3590 | 1522 |
| MTUB | 3054 | 3054 | 4252 | 3190 | 1748 | 2454 | 818 |
| PRAC | 2235 | 0 | 3471 | 2549 | 1384 | 3257 | 1052 |
| RHSP | 4901 | 3430 | 6133 | 5556 | 1386 | 953 | 1046 |
| STMU | 2305 | 482 | 3571 | 2987 | 911 | 3322 | 1296 |
| SYEL | 817 | 817 | 4212 | 3246 | 1124 | 2714 | 1179 |
| THAF | 3152 | 2366 | 3353 | 2373 | 949 | 3773 | 913 |
| THRO | 1148 | 1148 | 3220 | 1343 | 1729 | 2534 | 948 |
| THTH | 3027 | 22 | 3662 | 493 | 2856 | 1043 | 421 |
| THYE | 1652 | 18 | 2318 | 18 | 1040 | 4329 | 600 |
| Total | 54183 | 30073 | 76586 | 54105 | 25056 | 63757 | 23776 |

| **MetaBinT against NRminusGenus for read length ~45 bp** | | | | | | | |
| --- | --- | --- | --- | --- | --- | --- | --- |
| **GENOME** | **TFAMILY** | **CFAMILY** | **TPHYLUM** | **CPHYLUM** | **UNASS** | **NOHITS** | **INTERGENIC** |
| ACFE | 1168 | 1 | 1534 | 666 | 532 | 6058 | 1284 |
| AQAE | 2134 | 1508 | 2369 | 1776 | 312 | 5729 | 858 |
| ARBU | 1030 | 271 | 1908 | 1646 | 120 | 6628 | 904 |
| BOAF | 583 | 75 | 769 | 96 | 281 | 7600 | 792 |
| BSUB | 1564 | 630 | 2239 | 1815 | 258 | 5523 | 1348 |
| CBOT | 1007 | 76 | 1399 | 944 | 148 | 5755 | 2090 |
| CFP2 | 1100 | 1100 | 1918 | 1588 | 88 | 4301 | 3051 |
| CHAB | 2103 | 1833 | 2240 | 1969 | 118 | 5922 | 1388 |
| ECOLI | 6397 | 6339 | 7153 | 7119 | 20 | 430 | 1901 |
| FLJO | 877 | 315 | 2073 | 1792 | 174 | 6004 | 1407 |
| FUNU | 1136 | 290 | 1424 | 290 | 155 | 6430 | 1303 |
| LACB | 1373 | 407 | 1933 | 1669 | 101 | 5753 | 1767 |
| MTUB | 1793 | 1793 | 2386 | 1867 | 473 | 5633 | 1096 |
| PRAC | 1249 | 0 | 1934 | 1473 | 300 | 6037 | 1251 |
| RHSP | 3137 | 2235 | 4034 | 3730 | 442 | 3887 | 1263 |
| STMU | 1417 | 264 | 2061 | 1723 | 197 | 5489 | 1607 |
| SYEL | 565 | 565 | 2521 | 2025 | 203 | 5269 | 1435 |
| THAF | 1795 | 1298 | 1937 | 1301 | 122 | 6121 | 1208 |
| THRO | 603 | 603 | 1666 | 710 | 283 | 6036 | 1232 |
| THTH | 1568 | 24 | 1875 | 260 | 1740 | 4851 | 685 |
| THYE | 1016 | 12 | 1405 | 12 | 124 | 6780 | 867 |
| Total | 33615 | 19639 | 46778 | 34471 | 6191 | 116236 | 28737 |

**Symbols used in the columns**

**TGENUS**: Total reads assigned at genus level; **CGENUS**: Total reads assigned to correct genus; **TFAMILY**: Total reads assigned at family level; **CFAMILY**: Total reads assigned to correct family; **TPHYLUM**: Total reads assigned at phylum level; **CPHYLUM**: Total reads assigned to correct phylum; **UNASS**: Total reads which remained unassigned; **NOHITS**: Total reads for which no hit was found by alignment; **INTERGENIC**: Total reads originating from intergenic regions
